# Organocatalytic Enantioselective Friedel–Crafts Reaction of Phenanthrenequinones and Indoles

**DOI:** 10.3390/molecules30010172

**Published:** 2025-01-04

**Authors:** Yan Jin, Yuhong Sun, Yue Yu, Jiao Zhao, Mingshan Zheng, Liming Wang, Ying Jin

**Affiliations:** 1 Department of Pharmacy, Jilin Medical University, Jilin 132013, China; 2022010078@ybu.edu.cn (Y.J.); sunyuhong6012@163.com (Y.S.); 3496719936@163.com (J.Z.); 2 College of Science, Yanbian University, Yanji 133000, China; 3 School of Pharmaceutical Sciences, Yanbian University, Yanji 133000, China; 16604588858@163.com (Y.Y.); zhengmingshan@ybu.edu.cn (M.Z.)

**Keywords:** enantioselective, Friedel–Crafts reaction, organocatalysis, phenanthrenequinones, indoles, CCK-8 assay

## Abstract

An efficient stereoselective synthesis of 10-hydroxy-10-(1*H*-indol-3-yl)-9-(10*H*)-phenanthrene derivatives was realized through an organocatalyzed Friedel–Crafts reaction of phenanthrenequinones and indoles using a (*S*,*S*)-dimethylaminocyclohexyl-squaramide as the catalyst. Under the optimized conditions, the desired chiral products were obtained in good yields (73–90%) with moderate to high ee values (up to 97% ee). Two pairs of synthesized enantiomers were subjected to evaluation of their antiproliferative activities on four types of human cancer cell lines and one human umbilical vein endothelial cell line using the CCK-8 assay. The results indicated that stereoselectivity had obvious impacts on biological activity. (*S*)-**4g** was found to have optimal cytotoxicity against the A549 cell line and a good safety profile for human normal cells, which was better than the inhibitory activity of the positive control drug (doxorubicin).

## 1. Introduction

As well known, many chiral drugs exhibit different pharmacological activities and safety due to the difference of stereochemistry. For example, (*S*)-Ofloxacin, (*S*)-Finerenone, and (*S*)-Amlodipine show better bioactivity than do their *R*-enantiomers [1,2,3]. Thus, the development of enantioselective reactions to access optically active compounds would be of significance in drug discovery.

9,10-phenanthroquinone is an important skeleton that exists in many bioactive natural products and synthetic compounds [4,5,6,7,8,9,10], and they play an extremely important role in photochemistry, electrochemistry, and organic synthesis [11,12,13,14]. The C9 and C10 dicarbonyl groups of phenanthroquinone are prochiral nucleophilic addition sites, which could undergo asymmetric addition reactions with many nucleophiles to give chiral adducts. However, the enantioselective transformation with 9,10-phenanthroquinone as a substrate has been rarely reported [15,16].

Indole is an important heterocyclic structure found in various natural alkaloids and bioactive pharmaceuticals [17,18,19,20,21,22,23,24,25,26,27]. Due to their wide range of applications in the synthesis field, the direct functionalization of indoles has been extensively investigated, and many methods have been developed in great progress. Moreover, the asymmetric Friedel–Crafts alkylation of indoles with appropriate electrophilic reagents is one of the most important and efficient strategies to produce optically active indoles [28,29,30,31,32,33,34,35,36,37,38,39,40,41,42,43,44].

**Figure 1:**
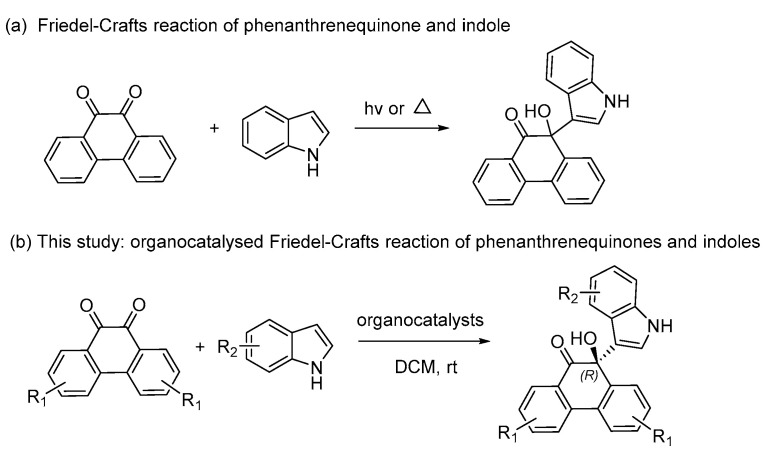
Friedel–Crafts reaction of 9,10-phenanthrene and indole.

In 1998, the Yan group reported one example of photochemical synthesis of 10-hydroxy-10-(1*H*-indol-3-yl)-9-(10*H*)-phenanthrene via Friedel–Crafts reaction of 9,10-phenanthrene and indole. However, the racemic product was obtained because no asymmetric synthesis was involved (Figure 1a) [45].

**Figure 2:**
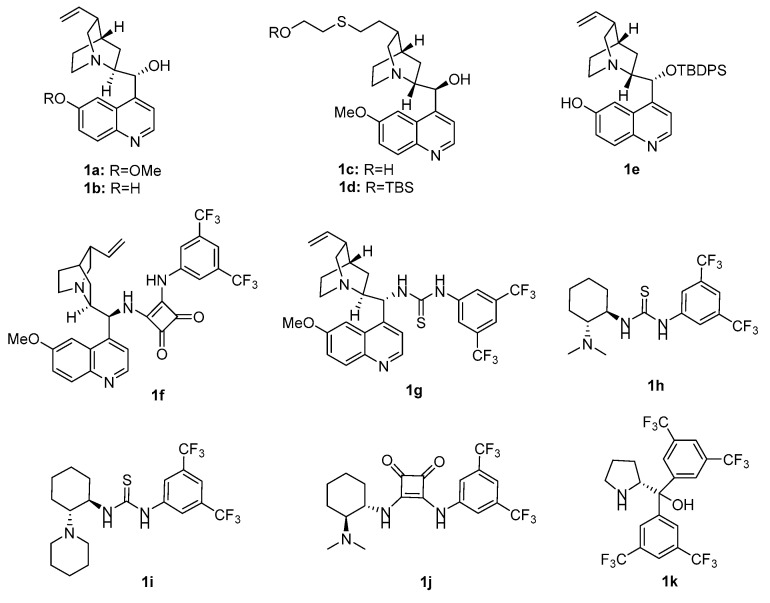
The structure of screened organocatalysts (**1a**–**1k**).

To the best of our knowledge, the asymmetric Friedel–Crafts reaction of phenanthrenequinones and indoles has never been reported to date. We herein first developed this reaction for the enantioselective construction of 10-hydroxy-10-(1*H*-indol-3-yl)-9-(10*H*)-phenanthrene (Figure 1b) by employing organocatalysts **1a**–**k** (Figure 2). In addition, this work aimed to develop an efficient and enantioselective method for synthesizing phenanthrenequinone derivatives, evaluating their biological properties, and exploring the impact of stereochemistry on antitumor activity.

## 2. Results and Discussion

We first applied the catalysts **1a**–**1k** in the Friedel–Crafts reaction of phenanthrenequinone (**2a**) and indole (**3a**) to screen the optimal catalyst. The reaction was carried out with CH_2_Cl_2_ as a solvent in the presence of 10 mol% of catalysts at room temperature for 24 h (Table 1).

All catalysts preceded the reaction smoothly to give the desired product **4a** in low yields (40–45%) with 5–40% ees. Among them, (*S*,*S*)-dimethylaminocyclohexy-squaramide **1j** was optimal in terms of the yield and enantioselectivity (entry 10). By monitoring the reaction (TLC), we found that indole was exhausted while the phenanthrenequinone was left obviously when the amount ratio of phenanthrenequinone (**2a**) to indole (**3a**) was 1:1. This might be the main reason for the low yields (entries 1–11). Therefore, we tried increasing the amount of indole by 3 to 10 equivalents in the reaction to improve the yield (entries 12–17). The results showed both yield and enantioselectivity were increased, and the optimal equivalent ratio was determined to be 1:7 of phenanthrenequinone to indole (entry 15), which would be further applied to the subsequent study.

To improve the enantioselectivity of the transformation, we investigated a variety of different reaction conditions (Table 2). The survey of solvents showed that CH_2_Cl_2_ was optimal in terms of the yield and enantioselectivity (entry 1), aligning with previous studies that CH_2_Cl_2_ was found to enhance reactivity and enantioselectivity in Friedel–Crafts reactions [39,40]. The screening of catalyst loading exhibited that 10 mol% of **1j** was optimal. Reducing the catalyst loading to 5 mol% led to an obvious decrease in enantioselectivity and yield (entry 9 vs. entry 1), and 20 mol% loading was proven not beneficial for the catalysis performance (entry 10 vs. entry 1). When the reaction temperature was lowered from rt to 0 °C, both enantioselectivity and yield of product were decreased (entry 11 vs. entry 1). Furthermore, diluting the reaction concentration by half was detrimental for yield and enantiocontrol (entry 12 vs. entry 1). Additives such as 4 Å molecular sieves or benzoic acid offered no improvement in the asymmetric induction (entry 14). Based on these experiments, the optimized conditions were determined to be CH_2_Cl_2_ as the solvent with a 10 mol% loading of catalyst **1j** at rt.

Firstly, the reaction of different substituted indoles **3** with 9,10-phenanthrenequinone (**2a**) was investigated. As Table 3 shows, most of the screened indoles were tolerated to give the corresponding products in good yields with 48–97% ees. When various substituents (R_2_) on the phenyl ring (C4, C5, C6, and C7) of indole were investigated, it was found that screened substituents at the C4 position showed a detrimental impact on the performance of the catalyst, resulting in a failure of the reaction (entries 2, 3). Next, the electron-donating groups at C5 (Me and OMe) were found to be favorable for enantioselectivity (entries 6, 7); especially, 5-OMe indole as a substrate gave the best enantiomeric excess of 97%. However, the reaction using 5-NO_2_ indole as a nucleophile failed to proceed even by prolonging the reaction time to 48 h (entry 8). In addition, the electron-withdrawing or electron-donating group at the C6 position showed a similar effect on the stereoselectivity to give moderate ee values (entries 9–12). It is worth mentioning that substituents at the C7 position had a great influence on the action of the catalyst. The reaction of 7-Me and 7-OMe indole proceeded smoothly to afford products with moderate enantioselectivities (entries 15, 16), while halogen groups at the C7 position of indole caused the reaction not to proceed even by prolonging the reaction time (entries 13, 14). Furthermore, when 2,7-dibromo- or 3,6-dibromo-substituted phenanthrenequinones were used as substrates in the reaction, similar ee values were obtained compared to the results from the reaction of phenanthrenequinone **2a** (entries 15–23), and the optimal enantioselectivities were observed in the reaction of 5-OMe indole (entries 19 and 23). Based on the above experimental results, the enantioselectivity was obviously affected by the substituted groups and their position. Among them, 5-OMe-substituted indole as a substrate gave the best enantiomeric excess (entries 7, 19, and 23).

**Figure 3:**
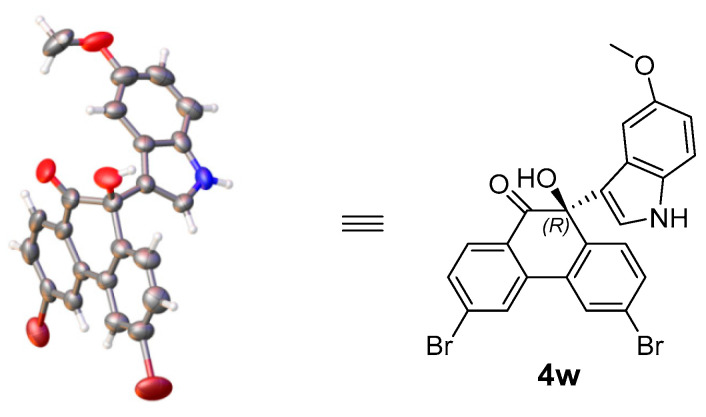
X-ray crystal structure of **4w** (CCDC 2375435).

The absolute configuration of 10-hydroxy-10-(1*H*-indol-3-yl)-9-(10*H*)-phenanthrene products **4** were unambiguously assigned as *R* according to the X-ray crystal structure analysis of **4w** (Figure 3) [46].

**Figure 4:**
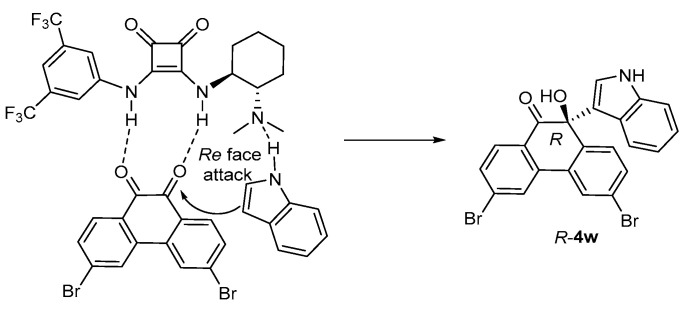
Proposed transition state.

A transition state model can be proposed according to the literature (Figure 4) [39,47]. As a bifunctional catalyst, the squaramide moiety of **1j** activates and orients phenanthrenequinone through double H-bonding, while the tertiary amine of **1j** activates indole through H-bonding. Then, *Re* face addition of indole to the activated phenanthrenequinone via favorable interaction leads to the formation of the (*R*)-product.

**Figure 5:**
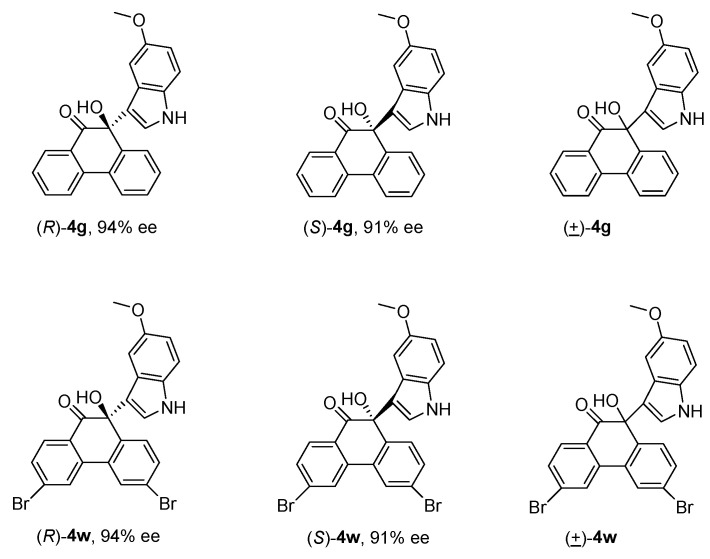
The structures of three pairs of enantiomers.

Next, we plan to study the antiproliferative activities of the obtained products. Moreover, to investigate the impact of the chiral center and electrical effect on activity, we chose products (*R*)-**4g** and (*R*)-**4w** with high ee values for the activity test. Their enantiomers of (*S*)-**4g** and (*S*)-**4w** were prepared by using the enantiomer of catalyst **1j**. The structures of enantiomers and their ee values were shown in Figure 5, and all tested compounds were determined to have a purity of >95%.

The antiproliferative activities of selected compounds and the positive control drug (doxorubicin) were evaluated on four types of human cancer cell lines and one human normal cell, human hepatocellular carcinoma cells (HepG-2), human non-small cell lung cancer cells (A549), human breast cancer cells (MCF-7), human gastric adenocarcinoma cells (SGC-7901), and human umbilical vein endothelial cells (HUVEC) using the CCK-8 assay (Table 4).

The results showed that most of the tested compounds had cytotoxic activity against human cancer cell lines in a concentration-dependent manner (see Supporting Information). The stereoselectivity showed an obvious impact on activity. Generally, *S*-configuration compounds were found to be more effective than *R*-enantiomers or racemic products. Among them, (*S*)-**4g** exhibited superior cytotoxic activity against the A549 (IC_50_: 0.38 ± 0.26 μM), which was obviously better than its enantiomer (*R*)-**4g** and racemate (±)-**4g**. Moreover, it is worth mentioning that the IC_50_ value of (*S*)-**4g** against the A549 was significantly lower than the value of the positive control (doxorubicin, IC_50_: 2.06 ± 0.14 μM). Specifically, (*S*)-**4g** showed substantially less cytotoxicity to HUVEC (IC_50_: 28.97 ± 1.22 μM) than that of doxorubicin (IC_50_: 0.44 ± 0.02 μM).

## 3. Materials and Methods

### 3.1. Experimental

^1^H NMR and ^13^C NMR spectra were recorded on a Brucker spectrometer (Karlsurhe, Germany) at 500 or 400 and 125 or 100 MHz, respectively. using CDCl_3_ and DMSO-*d*_6_ as a solvent. The chemical shifts were reported in ppm, and the residual nondeuterated solvent was used as an internal standard (CDCl_3_, 7.26 and 77.0 ppm; DMSO, 2.5 and 39.5 ppm, respectively). The splitting patterns of the signals were reported as s, singlet; d, doublet; t, triplet; q, quartet; dd, doublet of doublets; and m, multiplet. High-resolution mass spectra (HRMS) were measured on a triple TOF 5600+ mass spectrometer (SCIEX, Concord, ON, Canada) equipped with an electrospray ionization (ESI) source in the negative-ion mode. Single-crystal structure was determined on Bruker D8 Venture (Karlsurhe, Germany). The enantiomeric excess (ee) values of the products were determined by chiral HPLC, using Daicel Chiralcel OD-H, Chiralpak AD-H, and Chiralpak AS-H columns (4.6 mm × 250 mm). The reactions were monitored by thin-layer chromatography (TLC). Purifications by column chromatography were conducted over silica gel (200–300 mesh). The organocatalysts **1a**, **1b**, and **1f**–**1k** were purchased from Daicel Chiral Technologies (Shanghai, China), and the catalysts **1c**–**1e** were synthesized according to the literature [48].

### 3.2. General Procedure for the Enantioselective Friedel–Crafts Reaction of Phenanthrenequinones and Indoles

To a tube, a mixture of phenanthrenequinones (0.1 mmol), indoles (0.7 mmol) and organocatalyst **1j** (0.01 mmol), CH_2_Cl_2_ (1.0 mL) was added. The resulting mixture was stirred at 0 °C for 24 h. After the reaction was finished (monitored by TLC), the reaction was directly poured into a column chromatography on silica gel with hexane/EtOAc (4:1) as eluent to afford the new compounds **4a**–**y**. The enantiomeric ratio was determined by HPLC analysis on a chiral Chiralcel OD-H, AD-H, AS-H column. Experimental data can be found in Supplementary Materials.

(*R*)-**4a**: yellow solid, mp: 95.2–96.1 °C; ^1^H NMR (500 MHz, Chloroform-*d*) δ 7.98–7.93 (m, 1H), 7.92–7.84 (m, 3H), 7.79 (ddd, *J* = 7.5, 1.5, 0.5 Hz, 1H), 7.75–7.71 (m, 1H), 7.56 (ddd, *J* = 8.0, 7.5, 1.5 Hz, 1H), 7.53–7.46 (m, 2H), 7.27–7.23 (m, 1H), 7.22–7.19 (m, 1H), 7.16–7.09 (m, 2H), 6.36 (dd, *J* = 3.0, 2.0 Hz, 1H), 4.80 (s, 1H); ^13^C NMR (125 MHz, Chloroform-d) δ 200.9, 139.7, 137.0, 136.6, 134.5, 130.0, 129.4, 128.8, 128.6, 128.4, 127.8, 127.7, 125.3, 124.2, 123.7, 122.7, 122.4, 120.4, 117.7, 111.3; HRMS (ESI) *m*/*z*: [M + Na]^+^ calcd for C_22_H_15_NO_2_Na: 348.1000; found 348.1005; [α]_D_^25^ = 25.7 (c 0.50, MeOH) (70% ee); HPLC (Chiralcel OD-H, hexane:*^i^*PrOH = 82:18, 1.0 mL/min, 254 nm), t_R_ = 21.3 min (minor), 30.5 min (major).

(*R*)-**4d**: light yellow solid, mp: 174.1–175.0 °C; ^1^H NMR (500 MHz, Chloroform-*d*) δ 7.99–7.95 (m, 1H), 7.95–7.87 (m, 3H), 7.84 (ddd, *J* = 7.5, 1.5, 0.5 Hz, 1H), 7.60 (ddd, *J* = 8.0, 7.5, 1.5 Hz, 1H), 7.56–7.50 (m, 2H), 7.39 (ddt, *J* = 10.0, 2.5, 0.5 Hz, 1H), 7.31 (td, *J* = 7.5, 1.0 Hz, 1H), 7.16 (ddd, *J* =9.0, 4.5, 0.5 Hz, 1H), 6.91 (td, *J* = 9.0, 2.5 Hz, 1H), 6.46 (dd, *J* = 3.0, 1.0 Hz, 1H), 4.77 (s, 1H);^13^C NMR (125 MHz, Chloroform-*d*) δ 200.7, 158.0 (d, *J* = 247.5 Hz), 139.5, 137.0, 134.6, 133.1, 130.0, 129.4, 128.7, 128.6, 128.4, 127.8, 127.6, 125.8, 123.8, 122.8, 117.9, 111.9 (d, *J* = 15.0 Hz), 111.0 (d, *J* = 26.2 Hz), 105.6 (d, *J* = 25.0 Hz); HRMS (ESI) *m*/*z*: [M + Na]^+^ calcd for C_22_H_14_FNO_2_Na: 366.0906; found 366.0901; [α]_D_^25^ = +52.0 (c 0.51, MeOH) (48% ee); HPLC (Chiralcel OD-H, hexane:*^i^*PrOH = 75:25, 1.0 mL/min, 254 nm), t_R_ = 13.4 min (minor), 22.3 min (major).

(*R*)-**4e:** light yellow solid, mp: 191.5–192.4 °C; ^1^H NMR (500 MHz, Chloroform-*d*) δ 7.96–7.85 (m, 4H), 7.82 (ddd, *J* = 7.5, 1.5, 0.5 Hz, 1H), 7.71 (d, *J* = 2.0 Hz, 1H), 7.58 (ddd, *J* = 8.0, 7.5, 1.5 Hz, 1H), 7.53–7.46 (m, 2H), 7.28 (td, *J* = 7.5, 1.0 Hz, 1H), 7.12 (dd, *J* = 8.5, 0.5 Hz, 1H), 7.08 (dd, *J* = 8.5, 2.0 Hz, 1H), 6.39 (t, *J* = 2.5 Hz, 1H), 4.76 (s, 1H); ^13^C NMR (125 MHz, Chloroform-*d*) δ 200.7, 139.4, 137.0, 135.0, 134.7, 129.9, 129.5, 128.8, 128.6, 128.5, 127.9, 127.6, 126.2, 125.4, 123.8, 122.9, 122.8, 120.1, 117.6, 112.3; HRMS (ESI) *m*/*z*: [M + Na]^+^ calcd for C_22_H_14_ClNO_2_Na: 382.0611; found 382.0617; [α]_D_^25^ = +42.3 (c 0.58, MeOH) (48% ee); HPLC (Chiralcel OD-H, hexane:*^i^*PrOH = 75:25, 1.0 mL/min, 254 nm), t_R_ = 12.4 min (minor), 20.8 min (major).

(*R*)-**4f:** yellow solid, mp: 96.2–97.2 °C; ^1^H NMR (500 MHz, Chloroform-*d*) δ 7.96–7.91 (m, 1H), 7.91–7.88 (m, 1H), 7.88–7.85 (m, 1H), 7.80 (dd, *J* = 7.5, 1.5 Hz, 2H), 7.59–7.52 (m, 2H), 7.52–7.46 (m, 2H), 7.25 (td, *J* = 7.5, 1.0 Hz, 1H), 7.08 (d, *J* = 8.5 Hz, 1H), 6.95 (dd, *J* = 8.5, 1.5 Hz, 1H), 6.28 (d, *J* = 2.5 Hz, 1H), 4.79 (s, 1H), 2.45 (s, 3H);^13^C NMR (125 MHz, Chloroform-*d*) δ 200.8, 139.8, 137.0, 135.0, 134.4, 130.0, 129.7, 129.4, 128.8, 128.5, 128.3, 127.9, 127.7, 125.5, 124.3, 124.1, 123.6, 122.7, 120.0, 117.0, 111.0, 21.6; HRMS (ESI) *m*/*z*: [M + Na]^+^ calcd for C_23_H_17_NO_2_Na: 362.1157; found: 362.1151; [α]_D_^25^ = 28.9 (c 0.57, MeOH) (80% ee); HPLC (Chiralcel OD-H, hexane:*^i^*PrOH = 75:25, 1.0 mL/min, 254 nm), t_R_ = 11.2 min (minor), 16.8 min (major).

(*R*)-**4g**: yellow solid, mp: 200.8–201.8 °C; ^1^H NMR (500 MHz, Chloroform-*d*) δ 8.01–7.96 (m, 1H), 7.95–7.91 (m, 1H), 7.90 (ddt, *J* = 8.0, 1.0, 0.5 Hz, 1H), 7.84 (ddd, *J* = 7.5, 1.5, 0.5 Hz, 2H), 7.59 (ddd, *J* = 8.0, 7.5, 1.5 Hz, 1H), 7.54–7.49 (m, 2H), 7.29 (td, *J* = 7.5, 1.0 Hz, 1H), 7.16–7.10 (m, 2H), 6.81 (ddd, *J* = 9.0, 2.5, 0.5 Hz, 1H), 6.40 (dd, *J* = 3.0, 0.5 Hz, 1H), 4.77 (s, 1H), 3.88 (s, 3H); ^13^C NMR (125 MHz, Chloroform-*d*) δ 200.7, 154.5, 139.7, 137.0, 134.5, 131.7, 130.0, 129.4, 128.8, 128.6, 128.4, 127.8, 127.7, 125.7, 124.9, 123.6, 122.8, 117.1, 112.9, 112.0, 101.9, 55.8; HRMS (ESI) *m*/*z*: [M + Na]^+^ calcd for C_23_H_17_NO_3_Na: 378.1106; found: 378.1101; [α]_D_^25^= 35.6 (c 0.47, MeOH) (97% ee); HPLC (Chiralcel OD-H, hexane:*^i^*PrOH = 75:25, 1.0 mL/min, 254 nm), t_R_= 14.2 min (minor), 20.6 min (major).

(*R*)-**4i**: white solid, mp: 105.4–106.2 °C; ^1^H NMR (500 MHz, Chloroform-*d*) δ 7.99–7.93 (m, 1H), 7.92–7.83 (m, 3H), 7.79 (ddd, *J* = 7.7, 1.5, 0.5 Hz, 1H), 7.65 (ddt, *J* = 8.7, 5.3, 0.7 Hz, 1H), 7.58 (ddd, *J* = 8.0, 7.4, 1.5 Hz, 1H), 7.52–7.46 (m, 2H), 7.31–7.26 (m, 1H), 6.93–6.85 (m, 2H), 6.35 (dd, *J* = 2.7, 1.5 Hz, 1H), 4.75 (s, 1H);^13^C NMR (125 MHz, Chloroform-*d*) δ 201.0, 160.0 (d, *J* = 237.5 Hz), 139.5, 137.1, 136.7, 134.6, 130.0, 129.4, 128.7, 128.6, 128.4, 127.8, 127.6, 124.5, 123.7, 122.8, 121.9, 121.5 (d, *J* = 10.0 Hz), 118.0, 109.2 (d, *J* = 25.0 Hz), 97.6 (d, *J* = 25.0 Hz); HRMS (ESI) *m*/*z*: [M + Na]^+^ calcd for C_22_H_14_FNO_2_Na: 366.0906; found 366.0910; [α]_D_^25^ = 18.6 (c 0.52, MeOH) (54% ee); HPLC (Chiralcel OD-H, hexane:*^i^*PrOH = 75:25, 1.0 mL/min, 254 nm), t_R_ = 10.4 min (minor), 15.3 min (major).

(*R*)-**4j**: white solid, mp: 106.4–107.3 °C; ^1^H NMR (500 MHz, Chloroform-*d*) δ 7.98–7.85 (m, 4H), 7.78 (ddd, *J* = 7.5, 1.5, 0.5 Hz, 1H), 7.65–7.60 (m, 1H), 7.58 (ddd, *J* = 8.0, 7.5, 1.5 Hz, 1H), 7.53–7.46 (m, 2H), 7.30–7.26 (m, 1H), 7.20 (ddt, *J* = 2.0, 1.5, 0.5 Hz, 1H), 7.08 (dd, *J* = 8.5, 2.0 Hz, 1H), 6.58–6.13 (m, 1H), 4.76 (s, 1H);^13^C NMR (125 MHz, Chloroform-*d*) δ 200.9, 139.4, 137.0, 134.7, 130.0, 129.5, 128.8, 128.6, 128.5, 127.8, 127.6, 124.8, 123.9, 123.8, 122.8, 121.4, 121.2, 118.1, 111.2; HRMS (ESI) *m*/*z*: [M + Na]^+^ calcd for C_22_H_14_ClNO_2_Na: 382.0611; found 382.0615; [α]_D_^25^ = 36.4 (c 0.48, MeOH) (60% ee); HPLC (Chiralcel OD-H, hexane:*^i^*PrOH = 75:25, 1.0 mL/min, 254 nm), t_R_ = 10.4 min (minor), 15.1 min (major).

(*R*)-**4k**: white solid, mp: 181.9– 182.7 °C;^1^H NMR (500 MHz, Chloroform-*d*) δ 8.03–7.95 (m, 1H), 7.94–7.90 (m, 1H), 7.90–7.86 (m, 1H), 7.85–7.75 (m, 2H), 7.60 (d, *J* = 8.0 Hz, 1H), 7.57 (ddd, *J* = 8.0, 7.5, 1.5 Hz, 1H), 7.54–7.48 (m, 2H), 7.25 (td, *J* = 7.5, 1.0 Hz, 1H), 7.04–6.93 (m, 2H), 6.30 (d, *J* = 2.7 Hz, 1H), 4.80 (s, 1H), 2.41 (s, 3H);^13^C NMR (125 MHz, Chloroform-*d*) δ 200.9, 139.7, 137.1, 137.0, 134.4, 132.2, 130.0, 129.3, 128.8, 128.5, 128.3, 127.8, 127.6, 123.6, 123.1, 122.7, 122.2, 120.0, 117.5, 111.2, 21.5; HRMS (ESI) *m*/*z*: [M + Na]^+^ calcd for C_23_H_17_NO_2_Na: 362.1157; found: 362.1153; [α]_D_^25^ = 74.6 (c 0.60, MeOH) (56% ee); HPLC (Chiralcel OD-H, hexane:*^i^*PrOH = 75:25, 1.0 mL/min, 254 nm), t_R_ = 13.6 min (minor), 16.6 min (major).

(*R*)-**4l**: yellow solid, mp: 101.0–102.0 °C; ^1^H NMR (500 MHz, Chloroform-*d*) δ 7.98–7.91 (m, 1H), 7.91–7.87 (m, 1H), 7.85 (d, *J* = 8.0 Hz, 1H), 7.82–7.73 (m, 2H), 7.60–7.52 (m, 2H), 7.52–7.44 (m, 2H), 7.25 (td, *J* = 7.5, 1.0 Hz, 1H), 6.77 (dd, *J* = 9.0, 2.5 Hz, 1H), 6.65 (d, *J* = 2.5 Hz, 1H), 6.23 (d, *J* = 2.5 Hz, 1H), 4.76 (s, 1H), 3.75 (s, 3H); ^13^C NMR (126 MHz, Chloroform-*d*) δ 200.9, 156.5, 139.6, 137.5, 137.0, 134.5, 130.0, 129.3, 128.7, 128.6, 128.3, 127.8, 127.6, 123.6, 123.2, 122.7, 121.0, 119.6, 117.7, 110.3, 94.8, 55.5; HRMS (ESI) *m*/*z*: [M + Na]^+^ calcd for C_23_H_17_NO_3_Na: 378.1106; found: 378.1111; [α]_D_^25^ = 53.4 (c 0.50, MeOH) (54% ee); HPLC (Chiralcel OD-H, hexane:*^i^*PrOH = 75:25, 1.0 mL/min, 254 nm), t_R_ = 17.2 min (minor), 24.6 min (major).

(*R*)-**4o**: yellow solid, mp: 140.6–141.7 °C; ^1^H NMR (500 MHz, Chloroform-*d*) δ 7.98–7.93 (m, 1H), 7.92–7.88 (m, 1H), 7.87 (dd, *J* = 8.0, 1.0 Hz, 1H), 7.81–7.72 (m, 2H), 7.59–7.53 (m, 2H), 7.53–7.46 (m, 2H), 7.25 (td, *J* = 7.5, 1.0 Hz, 1H), 7.03 (dd, *J* = 8.0, 7.0 Hz, 1H), 6.94 (dt, *J* = 7.0, 1.0 Hz, 1H), 6.37 (dd, *J* = 6.0, 2.5 Hz, 1H), 4.79 (s, 1H), 2.32 (s, 3H);^13^C NMR (125 MHz, Chloroform-*d*) δ 200.8, 139.7, 137.0, 136.2, 134.5, 130.0, 129.4, 128.8, 128.6, 128.4, 127.8, 127.7, 124.8, 123.9, 123.7, 123.0, 122.7, 120.6, 120.4, 118.3, 118.1, 16.4; HRMS (ESI) *m*/*z*: [M + Na]^+^ calcd for C_23_H_17_NO_2_Nal: 362.1157; found: 362.1152; [α]_D_^25^ = 49.1 (c 0.53, MeOH) (52% ee); HPLC (Chiralcel OD-H, hexane:*^i^*PrOH = 75:25, 1.0 mL/min, 254 nm), t_R_ = 12.9 min (minor), 17.8 min (major).

(*R*)-**4p**: yellow solid, mp: 104.5–105.4 °C; ^1^H NMR (500 MHz, Chloroform-*d*) δ 8.12 (s, 1H), 8.03–7.97 (m, 1H), 7.95–7.91 (m, 1H), 7.91–7.86 (m, 1H), 7.82 (dd, *J* = 7.5, 1.5 Hz, 1H), 7.58 (ddd, *J* = 8.0, 7.5, 1.5 Hz, 1H), 7.55–7.49 (m, 2H), 7.32 (dt, *J* = 8.0, 1.0 Hz, 1H), 7.27 (td, *J* = 7.5, 1.0 Hz,1H), 7.05 (t, *J* = 8.0 Hz, 1H), 6.61 (dd, *J* = 8.0, 0.5 Hz, 1H), 6.38 (d, *J* = 2.5 Hz, 1H), 4.81 (s, 1H), 3.88 (s, 3H);^13^C NMR (125 MHz, Chloroform-*d*) δ 200.8, 146.1, 139.7, 137.0, 134.46, 130.0, 129.4, 128.8, 128.6, 128.4, 127.8, 127.7, 127.3, 126.6, 123.7, 122.8, 120.9, 118.2, 113.0, 102.2, 55.2; HRMS (ESI) *m*/*z*: [M + Na]^+^ calcd for C_23_H_17_NO_3_Na: 378.1106; found: 378.1103; [α]_D_^25^ = 31.4 (c 0.55, MeOH) (56% ee); HPLC (Chiralcel OD-H, hexane:*^i^*PrOH = 75:25, 1.0 mL/min, 254 nm), t_R_ = 24.9 min (minor), 28.1 min (major).

(*R*)-**4q**: orange solid, mp: 118.9–120.0 °C; ^1^H NMR (500 MHz, DMSO-*d*_6_) δ 10.97 (d, *J* = 3.0 Hz, 1H), 8.07 (dd, *J* = 8.5, 4.5 Hz, 2H), 7.97 (d, *J* = 2.0 Hz, 1H), 7.81 (dd, *J* = 8.5, 2.5 Hz, 1H), 7.74 (dd, *J* = 8.5, 2.0 Hz, 1H), 7.70 (d, *J* = 2.0 Hz, 1H), 7.66–7.62 (m, 1H), 7.29 (dt, *J* = 8.0, 1.0 Hz, 1H), 7.05 (ddd, *J* = 8.0, 7.0, 1.5 Hz, 1H), 6.98 (ddd, *J* = 8.0, 7.0, 1.0 Hz, 1H),6.53 (s, 1H), 6.39 (d, *J* = 2.5 Hz, 1H);^13^C NMR (125 MHz, DMSO-*d*_6_) δ 196.2, 143.9, 136.8, 136.6, 134.2, 131.4, 130.8, 130.2, 129.2, 128.4, 126.4, 126.0, 125.0, 124.6, 122.9, 121.8, 121.5, 120.6, 119.2, 114.7, 111.8, 77.5; HRMS (ESI) *m*/*z*: [M + Na]^+^ calcd for C_22_H_13_Br_2_NO_2_Na: 503.9211; found: 503.9215; [α]_D_^25^ = 58.0 (c 0.62, MeOH) (68% ee); HPLC (Chiralcel OD-H, hexane:*^i^*PrOH = 85:15, 1.0 mL/min, 254 nm), t_R_ = 13.6 min (major), 16.2 min (minor).

(*R*)-**4r**: orange solid, mp: 103.2–104.2 °C; ^1^H NMR (500 MHz, DMSO-*d*_6_) δ 10.83 (d, *J* = 3.0 Hz, 1H), 8.06 (dd, *J* = 8.5, 3.5 Hz, 2H), 7.95 (d, *J* = 2.0 Hz, 1H), 7.81 (dd, *J* = 8.5, 2.5 Hz, 1H), 7.73 (dd, *J* = 8.5, 2.0 Hz, 1H), 7.71 (d, *J* = 2.0 Hz, 1H), 7.47 (dd, *J* = 2.0, 1.0 Hz, 1H), 7.17 (dd, *J* = 8.5, 1.0 Hz, 1H), 6.88 (dd, *J* = 8.5, 1.5 Hz, 1H), 6.48 (s, 1H), 6.29 (d, *J* = 2.5 Hz, 1H), 2.35 (s, 3H); ^13^C NMR (125 MHz, DMSO-*d*_6_) δ 196.0, 144.0, 136.8, 135.0, 134.2, 131.4, 131.0, 130.8, 130.3, 129.2, 128.4, 127.6, 126.9, 126.3, 126.0, 125.3, 124.6, 123.2, 122.9, 121.8, 120.2, 114.2, 111.5, 77.6, 21.4; HRMS (ESI) *m*/*z*: [M + Na]^+^ calcd for C_23_H_15_Br_2_NO_2_Na: 517.9367; found: 517.9361; [α]_D_^25^ = 80.4 (c 0.59, MeOH) (63% ee); HPLC (Chiralcel OD-H, hexane:*^i^*PrOH = 93:7, 1.0 mL/min, 254 nm), t_R_ = 30.2 min (major), 33.1 min (minor).

(*R*)-**4s**: orange solid, mp: 101.5–102.7 °C; ^1^H NMR (500 MHz, DMSO-*d*_6_) δ 10.85 (d, *J* = 3.0 Hz, 1H), 8.06 (dd, *J* = 8.5, 5.0 Hz, 2H), 7.98 (d, *J* = 2.0 Hz, 1H), 7.81 (dd, *J* = 8.5, 2.5 Hz, 1H), 7.76–7.71 (m, 2H), 7.18 (d, *J* = 9.0 Hz, 1H), 7.04 (d, *J* = 2.5 Hz, 1H), 6.70 (dd, *J* = 9.0, 2.5 Hz, 1H), 6.51 (s, 1H), 6.38 (d, *J* = 3.0 Hz, 1H), 3.72 (s, 3H); ^13^C NMR (126 MHz, DMSO-*d*_6_) δ 195.9, 153.2, 143.8, 136.8, 134.2, 131.7, 131.4, 130.9, 130.3, 129.2, 128.4, 126.4, 126.0, 125.4, 125.3, 122.9, 121.9, 114.1, 112.5, 111.6, 102.3, 77.5, 55.2; HRMS (ESI) *m*/*z*: [M + Na]^+^ calcd for C_23_H_15_Br_2_NO_3_Na: 533.9316; found: 533.9319; [α]_D_^25^ = 23.4 (c 0.46, MeOH) (94% ee); HPLC (Chiralcel OD-H, hexane:*^i^*PrOH = 85:15, 1.0 mL/min, 254 nm), t_R_ = 17.3 min (major), 33.7 min (minor).

(*R*)-**4t**: orange solid, mp: 194.1–195.2 °C; ^1^H NMR (500 MHz, DMSO-*d*_6_) δ 11.07 (d, *J* = 2.5 Hz, 1H), 8.25 (d, *J* = 8.5 Hz, 1H), 8.13–8.06 (m, 3H), 7.96 (dd, *J* = 12.0, 2.5 Hz, 2H), 7.84 (dd, *J* = 8.5, 2.5 Hz, 1H), 7.75 (dd, *J* = 8.5, 2.0 Hz, 1H), 7.70 (d, *J* = 2.5 Hz, 1H), 7.66 (d, *J* = 8.5 Hz, 1H), 7.38–7.32 (m, 1H), 7.02 (dd, *J* = 8.5, 2.0 Hz, 1H), 6.58 (s, 1H), 6.42 (d, *J* = 2.5 Hz, 1H); ^13^C NMR (125 MHz, DMSO-*d*_6_) δ 196.1, 176.8, 137.4, 137.0, 136.9, 134.2, 133.5, 133.0, 131.5, 131.0, 130.5, 130.2, 129.2, 128.3, 126.8, 126.3, 126.0, 125.6, 123.7, 122.9, 122.7, 121.9, 121.8, 119.5, 115.1, 111.4, 77.3; HRMS (ESI) *m*/*z*: [M + Na]^+^ calcd for C_22_H_12_Br_2_ClNO_2_Na: 537.8821; found: 537.8826; [α]_D_^25^ = 73.1 (c 0.57, MeOH) (60% ee); HPLC (Chiralcel OD-H, hexane:*^i^*PrOH = 90:10, 1.0 mL/min, 254 nm), t_R_ = 17.8 min (minor), 20.1 min (major).

(*R*)-**4u**: orange solid, mp: 71.9–72.8 °C; ^1^H NMR (500 MHz, DMSO-*d*_6_) δ 10.74 (d, *J* = 2.5 Hz, 1H), 8.05 (dd, *J* = 8.5, 2.5 Hz, 2H), 7.96 (d, *J* = 2.0 Hz, 1H), 7.81 (dd, *J* = 8.5, 2.5 Hz, 1H), 7.72 (dd, *J* = 8.5, 2.0 Hz, 1H), 7.69 (d, *J* = 2.5 Hz, 1H), 7.49 (d, *J* = 9.0 Hz, 1H), 6.77 (d, *J* = 2.5 Hz, 1H), 6.65 (dd, *J* = 9.0, 2.5 Hz, 1H), 6.49 (s, 1H), 6.23 (d, *J* = 2.5 Hz, 1H), 3.71 (s, 3H); ^13^C NMR (125 MHz, DMSO-*d*_6_) δ 196.3, 155.7, 143.9, 137.4, 136.8, 134.3, 131.4, 130.8, 130.2, 129.2, 128.4, 126.3, 126.0, 123.4, 122.9, 121.8, 121.2, 119.4, 114.8, 109.5, 94.8, 77.6, 55.1; HRMS (ESI) *m*/*z*: [M + Na]^+^ calcd for C_23_H_15_Br_2_NO_3_Na: 533.9316; found: 533.9312; [α]_D_^25^ = 23.7 (c 0.49, MeOH) (52% ee); HPLC (Chiralcel OD-H, hexane:*^i^*PrOH = 85:15, 1.0 mL/min, 254 nm), t_R_ = 17.8 min (minor), 19.9 min (major).

(*R*)-**4v**: orange solid, mp: 213.9–214.5 °C; ^1^H NMR (500 MHz, DMSO-*d*_6_) δ 10.79 (d, *J* = 3.0 Hz, 1H), 8.43 (dd, *J* = 6.0, 2.0 Hz, 2H), 7.79–7.71 (m, 2H), 7.61–7.51 (m, 2H), 7.51–7.44 (m, 1H), 7.21–7.09 (m, 1H), 6.87 (dd, *J* = 8.5, 1.5 Hz, 1H), 6.36 (s, 1H), 6.27 (d, *J* = 2.5 Hz, 1H), 2.39 (s, 3H); ^13^C NMR (125 MHz, DMSO-*d*_6_) δ 196.9, 141.3, 136.7, 135.0, 132.4, 132.1, 131.0, 129.8, 129.1, 128.6, 128.4, 127.4, 127.0, 126.6, 125.4, 124.5, 123.0, 122.1, 120.3, 114.6, 111.4, 77.5, 21.4; HRMS (ESI) *m*/*z*: [M + Na]^+^ calcd for C_23_H_15_Br_2_NO_2_Na: 517.9367; found: 517.9363; [α]_D_^25^ = 84.18 (c 0.55, MeOH) (60% ee); HPLC (Chiralcel OD-H, hexane:*^i^*PrOH = 90:10, 1.0 mL/min, 254 nm), t_R_ = 22.7 min (minor), 25.0 min (major).

(*R*)-**4w**: yellow solid, mp: 244.2–245.1 °C; ^1^H NMR (500 MHz, DMSO-*d*_6_) δ 10.80 (d, *J* = 3.0 Hz, 1H), 8.44 (dd, *J* = 5.0, 1.5 Hz, 2H), 7.80 (d, *J* = 8.5 Hz, 1H), 7.76–7.72 (m, 1H), 7.60 (d, *J* = 8.0 Hz, 1H), 7.54 (dd, *J* = 8.0, 1.5 Hz, 1H), 7.16 (d, *J* = 9.0 Hz, 1H), 7.03 (d, *J* = 2.5 Hz, 1H), 6.69 (dd, *J* = 9.0, 2.5 Hz, 1H), 6.38 (dd, *J* = 11.0, 2.5 Hz, 2H), 3.72 (s, 3H); ^13^C NMR (125 MHz, DMSO-*d*_6_) δ 196.8, 153.2, 141.1, 136.7, 132.4, 132.1, 131.7, 131.0, 129.9, 129.1, 128.7, 128.4, 127.0, 126.6, 125.5, 125.2, 122.1, 114.6, 112.4, 111.4, 102.4, 77.3, 55.2; HRMS (ESI) *m*/*z*: [M + Na]^+^ calcd for C_23_H_15_Br_2_NO_3_Na: 533.9316; found: 533.9311; [α]_D_^25^ = 581.8 (c 0.61, MeOH) (97% ee); HPLC (Chiralcel OD-H, hexane:*^i^*PrOH = 85:15, 1.0 mL/min, 254 nm), t_R_ = 19.5 min (minor), 23.2 min (major).

(*R*)-**4x**: yellow solid, mp: 216.3–217.4 °C; ^1^H NMR (500 MHz, DMSO-*d*_6_) δ 11.04 (d, *J* = 2.5 Hz, 1H), 8.64 (d, *J* = 2.0 Hz, 2H), 8.44 (dd, *J* = 6.0, 2.0 Hz, 2H), 7.93 (d, *J* = 8.5 Hz, 2H), 7.78–7.72 (m, 4H), 7.68 (d, *J* = 8.5 Hz, 1H), 7.61–7.51 (m, 2H), 7.32 (d, *J* = 2.0 Hz, 1H), 7.01 (dd, *J* = 8.5, 2.0 Hz, 1H), 6.48 (s, 1H), 6.41 (d, *J* = 2.5 Hz, 1H); ^13^C NMR (125 MHz, DMSO-*d*_6_) δ 197.0, 177.7, 140.9, 137.0, 136.7, 135.9, 132.7, 132.5, 132.2, 130.9, 130.8, 130.7, 130.0, 129.8, 129.1, 128.9, 128.1, 127.7, 127.1, 126.7, 126.3, 125.6, 123.8, 122.2, 122.1, 119.5, 115.6, 111.4, 77.2; HRMS (ESI) *m*/*z*: [M + Na]^+^ calcd for C_22_H_12_Br_2_ClNO_2_Na: 537.8821; found: 537.8825; [α]_D_^25^ =82.0 (c 0.49, MeOH) (58% ee); HPLC (Chiralpak AD-H, hexane:*^i^*PrOH = 70:30, 1.0 mL/min, 254 nm), t_R_ = 15.9 min (major), 24.3 min (minor).

(*R*)-**4y**: yellow solid, mp: 122.7–123.3 °C; ^1^H NMR (500 MHz, DMSO-*d*_6_) δ 10.71 (d, *J* = 2.5 Hz, 1H), 8.43 (dd, *J* = 8.0, 2.0 Hz, 2H), 7.78 (d, *J* = 8.5 Hz, 1H), 7.73 (dd, *J* = 8.5, 2.0 Hz, 1H), 7.58–7.53 (m, 2H), 7.52–7.48 (m, 1H), 6.76 (dd, *J* = 2.5, 0.5 Hz, 1H), 6.63 (dd, *J* = 9.0, 2.5 Hz, 1H), 6.37 (s, 1H), 6.21 (d, *J* = 2.5 Hz, 1H), 3.70 (s, 3H); ^13^C NMR (125 MHz, DMSO-*d*_6_) δ 197.1, 155.6, 141.2, 137.4, 136.7, 132.4, 132.1, 130.9, 129.8, 129.0, 128.7, 128.4, 127.0, 126.6, 123.3, 122.0, 121.2, 119.4, 115.3, 109.3, 94.7, 77.4, 55.1; HRMS (ESI) *m*/*z*: [M + Na]^+^ calcd for C_23_H_15_Br_2_NO_3_Na: 533.9316; found: 533.9312; [α]_D_^25^ = 82.8 (c 0.52, MeOH) (56% ee); HPLC (Chiralpak AS-H, hexane:*^i^*PrOH = 80:20, 1.0 mL/min, 254 nm), t_R_ = 17.4 min (minor), 26.4 min (major).

(*S*)-**4g**: yellow solid, mp: 200.5–201.3 °C; ^1^H NMR (400 MHz, DMSO-*d*_6_) δ 10.83–10.69 (m, 1H), 8.11 (dd, *J* = 8.8, 4.0 Hz, 2H), 7.93–7.82 (m, 1H), 7.68 (d, *J* = 7.7 Hz, 1H), 7.63 (t, *J* = 7.6 Hz, 1H), 7.58–7.47 (m, 2H), 7.32 (t, *J* = 7.5 Hz, 1H), 7.15 (d, *J* = 8.8 Hz, 1H), 7.06 (d, *J* = 2.5 Hz, 1H), 6.67 (dd, *J* = 8.9, 2.5 Hz, 1H), 6.31 (d, *J* = 2.7 Hz, 1H), 6.24 (s, 1H), 3.70 (s, 3H); ^13^C NMR (101 MHz, DMSO) δ 198.6, 153.0, 141.5, 136.1, 134.2, 131.6, 129.8, 129.4, 129.0, 128.4, 128.4, 127.6, 127.0, 125.6, 125.0, 123.8, 123.2, 115.6, 112.2, 111.3, 102.5, 776, 55.2; HRMS (ESI) *m*/*z*: [M + Na]^+^ calcd for C_23_H_17_NO_3_Na: 378.1106; found: 378.1102; [α]_D_^25^ = 34.0 (c 0.50, MeOH) (91% ee); HPLC (Chiralcel OD-H, hexane:*^i^*PrOH = 75:25, 1.0 mL/min, 254 nm), t_R_ = 13.9 min (major), 21.3 min (minor).

(*S*)-**4w**: yellow solid, mp: 243.8–244.5 °C; ^1^H NMR (400 MHz, DMSO-*d*_6_) δ 10.86 (d, *J* = 2.9 Hz, 1H), 8.46 (dd, *J* = 3.4, 1.8 Hz, 2H), 7.82 (d, *J* = 8.4 Hz, 1H), 7.75 (dd, *J* = 8.4, 1.9 Hz, 1H), 7.61 (d, *J* = 8.2 Hz, 1H), 7.54 (dd, *J* = 8.2, 1.8 Hz, 1H), 7.18 (d, *J* = 8.8 Hz, 1H), 7.07 (d, *J* = 2.4 Hz, 1H), 6.70 (dd, *J* = 8.8, 2.5 Hz, 1H), 6.43 (s, 1H), 6.38 (d, *J* = 2.6 Hz, 1H); ^13^C NMR (101 MHz, DMF) δ 196.8, 153.2, 141.1, 136.7, 132.4, 132.1, 131.7, 131.0, 129.9, 129.1, 128.7, 128.4, 127.0, 126.6, 125.5, 125.2, 122.1, 114.6, 112.4, 111.5, 102.4, 77.4, 55.2; HRMS (ESI) *m*/*z*: [M + Na]^+^ calcd for C_23_H_15_Br_2_NO_3_Na: 533.9316; found: 533.9313; [α]_D_^25^ = 536.9 (c 0.51, MeOH) (91% ee); HPLC (Chiralcel OD-H, hexane:*^i^*PrOH = 85:15, 1.0 mL/min, 254 nm), t_R_ = 19.0 min (major), 23.3 min (minor).

## 4. Conclusions

In summary, we have described the first enantioselective Friedel–Crafts reaction of phenanthrenequinones and indoles organocatalyzed by (*S*,*S*)-squaramide to synthesize 10-hydroxy-10-(1*H*-indol-3-yl)-9-(10*H*)-phenanthrene derivatives in good yield with up to 97% enantioselectivity. Moreover, we used our optimized conditions to expand upon the substrate scope of this reaction. In addition, two pairs of synthesized enantiomers were subjected to evaluation of their cytotoxic properties against different human cancer cell lines and one human umbilical vein endothelial cell. Compared to doxorubicin, (*S*)-**4g** was found to be better not only for activity but also for safety. Therefore, 10-hydroxy-10-(1*H*-indol-3-yl)-9-(10*H*)-phenanthrene derivatives might be developed as antitumor candidate compounds after further research.

## Figures and Tables

**Table 1:** Asymmetric F–C reaction of phenanthroquinone and indole catalyzed by **1a**–**k** ^a^.

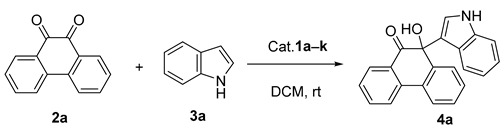				
Entry	Catalyst	2a:3a	Yield (%) ^b^	%ee ^c^
1	**1a**	1:1	43	20
2	**1b**	1:1	45	5
3	**1c**	1:1	40	10
4	**1d**	1:1	42	22
5	**1e**	1:1	40	21
6	**1f**	1:1	45	30
7	**1g**	1:1	42	26
8	**1h**	1:1	40	15
9	**1i**	1:1	41	24
10	**1j**	1:1	45	40
11	**1k**	1:1	40	20
12	**1j**	1:3	53	42
13	**1j**	1:5	78	48
14	**1j**	1:6	85	65
15	**1j**	1:7	90	70
16	**1j**	1:8	88	66
17	**1j**	1:10	90	60

^a^ Reaction condition: phenanthrenequinone (0.10 mmol), indole (0.10–1.00 mmol) and catalysts (0.01 mmol) in CH_2_Cl_2_ (1.0 mL) at rt. ^b^ isolated yield. ^c^ Determined by HPLC analysis (Chiralcel OD-H).

**Table 2:** Screening of reaction conditions for the asymmetric F–C reaction catalyzed by **1j** ^a^.

Entry	Solvent	Cat. Loading (% mmol)	Yield (%) ^b^	%ee ^c^
1	CH_2_Cl_2_	10	90	70
2	CHCl_3_	10	86	54
3	(CH_2_)_2_Cl_2_	10	79	46
4	Et_2_O	10	-	-
5	THF	10	-	-
6	PhMe	10	76	14
7	EA	10	75	12
8	ACN	10	83	41
9	CH_2_Cl_2_	5	77	50
10	CH_2_Cl_2_	20	88	61
11 ^d^	CH_2_Cl_2_	10	83	58
12 ^e^	CH_2_Cl_2_	10	80	62
13 ^f^	CH_2_Cl_2_	10	82	56
14 ^g^	CH_2_Cl_2_	10	78	38

^a^ Reaction condition: phenanthrenequinone (0.10 mmol), indole (0.70 mmol), and catalyst **1j** in solvent. ^b^ isolated yield. ^c^ Determined by HPLC analysis (Chiralcel OD-H). ^d^ 0 °C. ^e^ 2 mL of solvent. ^f^ 4 Å MS (about 200 mg). ^g^ Benzoic acid (0.01 mmol).

**Table 3:** Generality of the enantioselective F–C reaction of phenanthrenequinones and indoles ^a^.

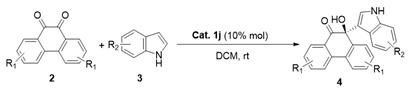					
Entry	R_1_	R_2_	Product	Yield (%) ^b^	%ee ^c^
1	H, H	H	**4a**	90	70
2	H, H	4-Cl	**4b**	-	-
3	H, H	4-Me	**4c**	-	-
4	H, H	5-F	**4d**	88	48
5	H, H	5-Cl	**4e**	85	48
6	H, H	5-Me	**4f**	87	80
7	H, H	5-OMe	**4g**	90	97
8	H, H	5-NO_2_	**4h**	-	-
9	H, H	6-F	**4i**	84	54
10	H, H	6-Cl	**4j**	82	60
11	H, H	6-Me	**4k**	75	56
12	H, H	6-OMe	**4l**	80	54
13	H, H	7-F	**4m**	-	-
14	H, H	7-Cl	**4n**	-	-
15	H, H	7-Me	**4o**	78	52
16	H, H	7-OMe	**4p**	83	56
17	2-Br, 7-Br	H	**4q**	81	68
18	2-Br, 7-Br	5-Me	**4r**	82	63
19	2-Br, 7-Br	5-OMe	**4s**	85	94
20	2-Br, 7-Br	6-Cl	**4t**	80	60
21	2-Br, 7-Br	6-OMe	**4u**	78	52
22	3-Br, 6-Br	5-Me	**4v**	83	60
23	3-Br, 6-Br	5-OMe	**4w**	90	97
24	3-Br, 6-Br	6-Cl	**4x**	75	58
25	3-Br, 6-Br	6-OMe	**4y**	73	56

^a^ Reaction condition: phenanthrenequinone (0.10 mmol), indole (0.70 mmol), and catalyst **1j** (0.01 mmol) in CH_2_Cl_2_ (1 mL) at rt. ^b^ Isolated yield. ^c^ Determined by HPLC analysis (Chiralcel OD-H, Chiralpak AD-H, AS-H).

**Table 4:** In vitro cytotoxicity of 10-hydroxy-10-(1*H*-indol-3-yl)-9-(10*H*)-phenanthrene derivatives against HepG-2, A549, MCF-7, SGC-7901, and HUVEC by CCK-8 assay (n = 3).

Entry	Compounds	IC_50_ Values ^a^ (μM)				
		HepG-2	A549	MCF-7	SGC-7901	HUVEC
1	(*R*)-**4g**	81.8 ± 0.96	36.17 ± 0.69	59.95 ± 3.43	59.89 ± 0.68	61.3 ± 3.42
2	(*S*)-**4g**	36.65 ± 3.76	0.38 ± 0.26	34.15 ± 1.37	29.88 ± 0.45	28.97 ± 1.22
3	(Racemic)-**4g**	- ^b^	2.5 ± 0.76	- ^b^	- ^b^	- ^b^
4	(*R*)-**4w**	23.50 ± 0.41	16.58 ± 0.27	18.62 ± 0.95	17.07 ± 0.03	22.53 ± 0.32
5	(*S*)-**4w**	13.44 ± 0.51	12.79 ± 0.31	5.89 ± 0.57	16.69 ± 0.17	20.03 ± 0.20
6	(Racemic)-**4w**	14.15 ± 0.22	15.09 ± 0.28	15.18 ± 0.41	17.41 ± 0.08	16.06 ± 0.11
7 ^c^	Doxorubicin	0.12 ± 0.01	2.06 ± 0.14	1.05 ± 0.04	0.88 ± 0.09	0.44 ± 0.02

^a^ IC_50_ is defined as the concentration, which results in a 50% decrease in cell number as compared with that of the control cultures in the absence of an inhibitor and were calculated using the respective regression analysis. ^b^ - = no biological activity. ^c^ Doxorubicin was employed as positive control.

## Data Availability

Data are contained within the article and Supplementary Materials.

## Supplementary Materials

The following supporting information can be downloaded at: https://www.mdpi.com/article/10.3390/molecules30010172/s1, Page S3–S24: ^1^H NMR and ^13^C NMR spectra; Page S25–S46: HPLC trace; Page S47–S48: X-Ray crystal data of compound **4w**; Page S49–S50: In vitro cytotoxicity assay.
