# Supplementary material for: Organocatalytic Enantioselective Friedel–Crafts Reaction of Phenanthrenequinones and Indoles
Source: Molecules. 2025 Jan 4;30(1):172. doi: 10.3390/molecules30010172 (PMC11721978; doi:10.3390/molecules30010172)
Supplement: Supplementary file 1 [file molecules-30-00172-s001.zip › molecules-3383158-supplementary.pdf]

Supplementary Information

Organocatalytic Enantioselective Friedel-Crafts Reaction of Phenanthrenequinones and Indoles

Yan Jin [1,2,†], Yuhong Sun [1,3,†], Yue Yu [3], Jiao Zhao [1], Mingshan Zheng [3], Liming Wang [1,*] and Ying Jin [1,2,3,*]

1 Department of Pharmacy, Jilin Medical University, Jilin 132013, China; 2022010078@ybu.edu.cn (Y. J.); sunyuhong6012@163.com (Y.S.); 3496719936@163.com (J.Z.)
2 College of Science, Yanbian University, Yanji 133000, China
3 School of Pharmaceutical Sciences, Yanbian University, Yanji 133000, China; 16604588858@163.com (Y.Y.); zhengmingshan@ybu.edu.cn (M.Z.)
* Correspondence: 13630635312@163.com (L.W.); jinying2288@163.com (Y.J.)
† These authors contributed equally to this work.

Table of Contents

1. General Information S1

2. ^{1}H NMR and ^{13}C NMR spectra S3

3.HPLC trace S25

4.X-Ray crystal data of comopound **4w** S47

5. In vitro cytotoxicity assay S49

1. General Information

The ^1H NMR spectra were recorded on a 500 MHz for ^1H and at 125 MHz for ^{13}C NMR, using $CDCl_3$ and DMSO-d_6 as a solvent. The chemical shifts were reported in ppm, and the residual nondeuterated solvent as internal standard ($CDCl_3$, 7.26 and 77.0 ppm, DMSO, 2.5 and 39.5 ppm, respectively). The splitting patterns of the signals were reported as s, singlet; d, doublet; t, triplet; q, quartet; dd, doublet of doublets; and m, multiplet. High-resolution mass spectra (HRMS) were measured on a triple TOF 5600+ mass spectrometer equipped with electrospray ionization (ESI) source in the negative-ion mode. Single-crystal structure was determined on Bruker D8 Venture. The enantiomeric excess (ee) values of the products were determined by chiral HPLC, using Daicel Chiralcel OD-H, Chiralpak AD-H and Chiralpak AS-H columns (4.6 mm*250 mm). The reactions were monitored by thin layer chromatography (TLC). Purifications by column chromatography were conducted over silica gel (200–300 mesh). The organocatalysts **1a**, **1b**, **1f–1k** were purchased from Daicel chiral technologies (China) company, and the catalysts **1c-1e** were synthesized according to the literature [1]. The information of materials and reagents used in the study were listed in Table S1.

Table S1. The information of materials and reagents used in the experiment.

Drugs and reagents	Chemical Formula	Purity (%)	Manufacturer
Indole	C_8H_7N	>98.5	Aladdine
4-Chloroindoline	$C_8H_4ClNO_2$	97.0	Bidepharm
4-Methylindoline	C_9H_9N	97.0	Bidepharm
5-Fluoroindoline	C_8H_6FNO	98.0	Bidepharm
5-Chloroindoline	$C_8H_4ClNO_2$	98.0	Bidepharm
5-Methylindoline	C_9H_9N	98.0	Bidepharm
5-Methoxyindole	C_9H_9NO	99.0	Bidepharm
6-Fluoroindoline	C_8H_6FNO	98.0	Bidepharm
6-Chloroindoline	$C_8H_4ClNO_2$	98.0	Bidepharm
6-Methylindoline	C_9H_9N	98.0	Bidepharm
6-Methoxyindole	C_9H_9NO	98.0	Bidepharm
7-Fluoroindoline	C_8H_6FNO	98.0	Bidepharm
7-Chloroindoline	$C_8H_4ClNO_2$	98.0	Bidepharm
7-Methylindoline	C_9H_9N	98.0	Bidepharm
7-Methoxyindole	C_9H_9NO	97.0	Bidepharm
9, 10- Phenanthrenequinone	$C_{14}H_8O_2$	98.0	Bidepharm
2, 7-Dibromo-9, 10-phenanthrenequinone	$C_{14}H_6Br_2O_2$	97.0	Bidepharm
3, 6-Dibromo-9, 10-phenanthrenequinone	$C_{14}H_6Br_2O_2$	97.0	Bidepharm
Triethylenediamine	$C_6H_{12}N_2$	98.0	Shanghai Darui Fine Chemicals Co., Ltd
Dichloromethane	CH_2Cl_2	≥99.0	Tianjin Damao Chemical Reagent Factory
Chloroform	$CHCl_3$	≥99.0	Tianjin Damao Chemical Reagent Factory
1,2-Dichloroethane	$(CH_2)_2Cl_2$	≥99.0	Tianjin Beichen Fangzheng Reagent Factory
Ethyl ether	$C_4H_{10}O$	≥99.5	Tianjin Damao Chemical Reagent Factory
Tetrahydrofuran	C_4H_8O	≥99.0	Tianjin Damao Chemical Reagent Factory
Acetonitrile	CH_3CN	99.0	Tianjin Damao Chemical Reagent Factory

Toluene	C_7H_8	≥99.5	Tianjin Damao Chemical Reagent Factory
n-Hexane	C_6H_{14}	99.0	Tianjin Damao Chemical Reagent Factory
Ethyl Acetate	$C_4H_8O_2$	99.0	Tianjin Damao Chemical Reagent Factory
HPLC Grade n-Hexane	C_6H_{14}	≥97.0	Beijing Mairuida Technology Co., Ltd
HPLC Grade Isopropanol	C_3H_8O	99.9	Beijing Mairuida Technology Co., Ltd
HPLC Grade Methanol	CH_3OH	99.9	Fisher Chemical
Chloroform-d	$CDCl_3$	99.8	Beijing Mairuida Technology Co., Ltd
Dimethyl sulfoxide-d6	C_2D_6SO	99.8	Beijing Mairuida Technology Co., Ltd

Reference

[1] Zhang TY, He W, Zhao XY, Jin Y, (2013) Asymmetric oxaziridination catalyzed by cinchona alkaloid derivatives containing sulfide. Tetrahedron 69: 7416-7422

2. ^{1}H NMR and ^{13}C NMR spectra

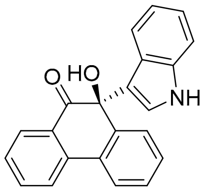

(*R*)-4a

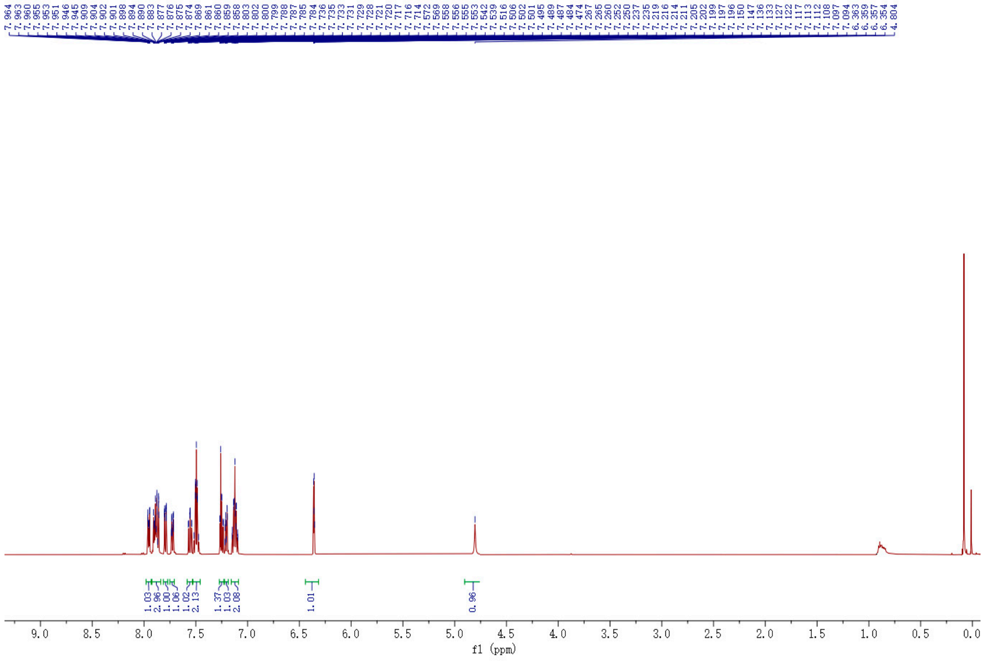

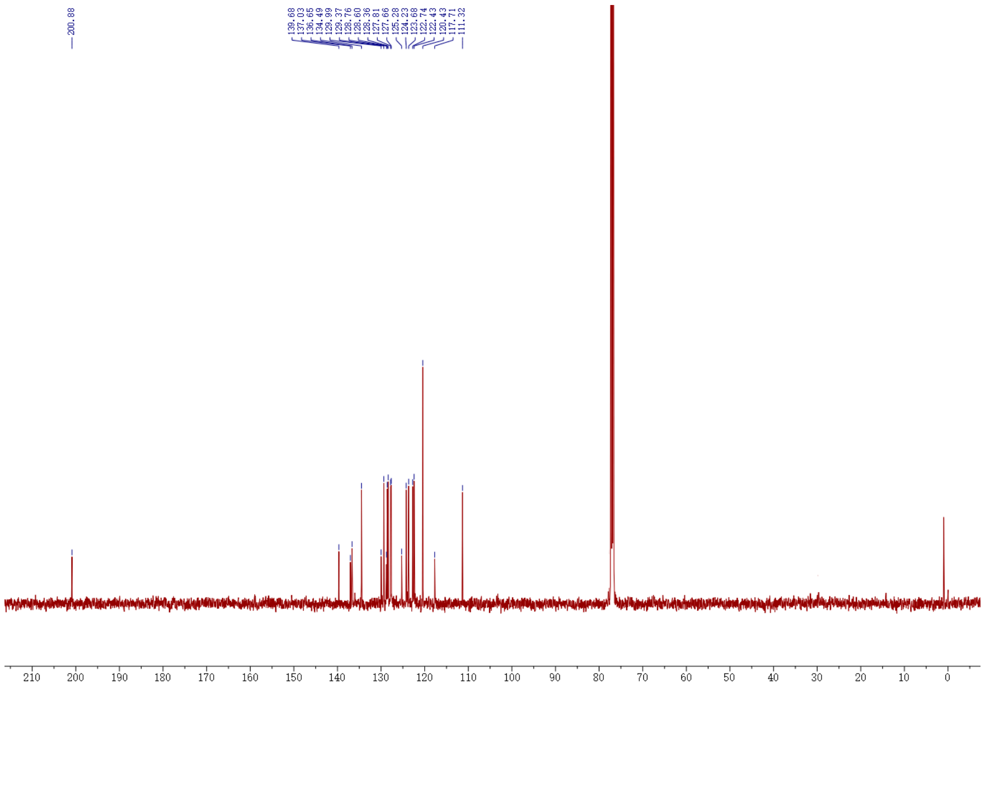

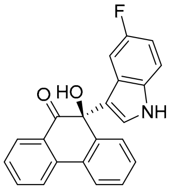

(*R*)-4d

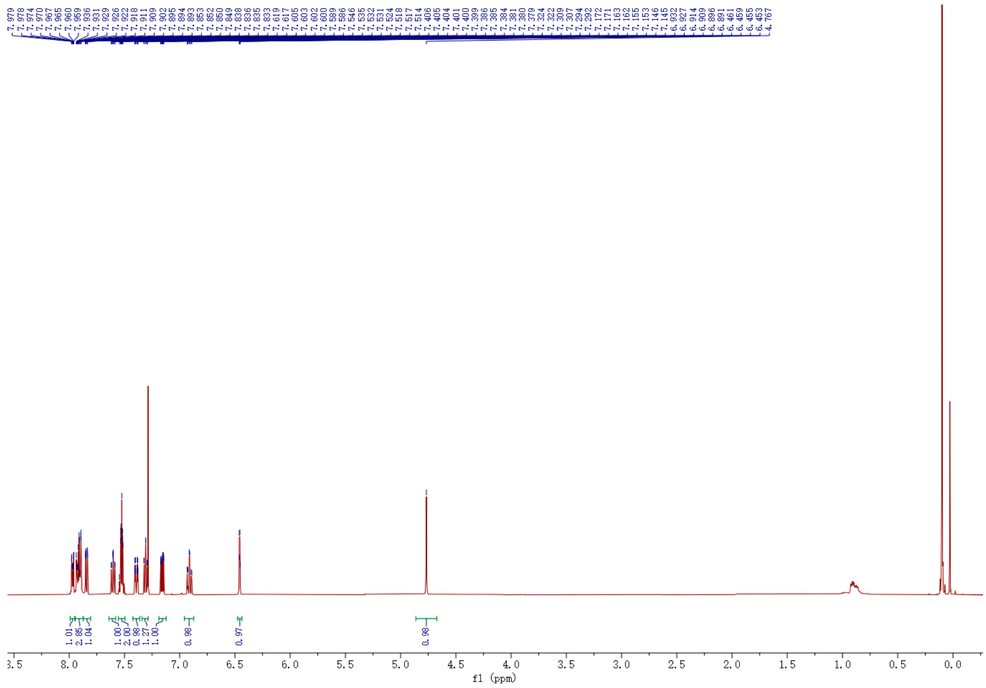

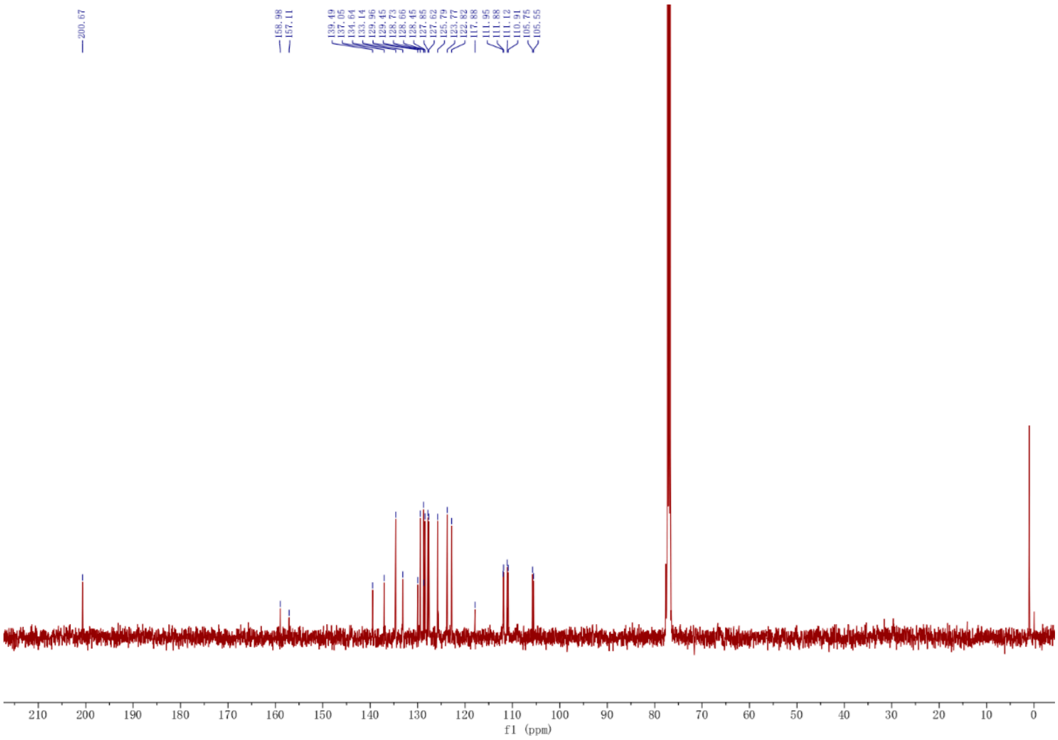

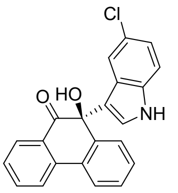
Cl
O
HO
NH

(*R*)-4e

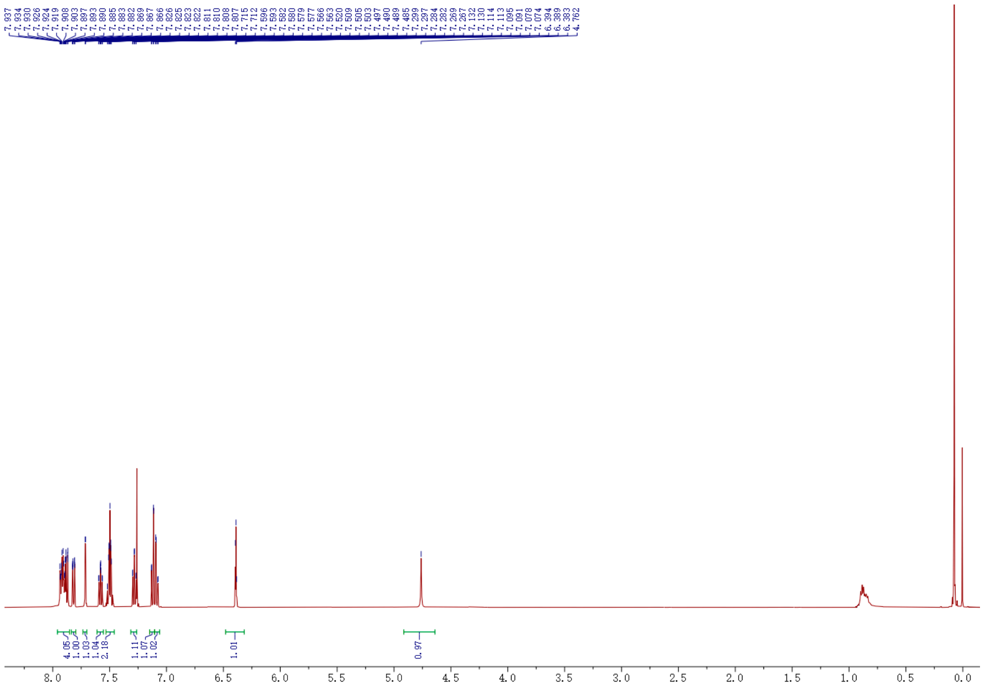
7.937 7.934 7.930 7.926 7.924 7.919 7.908 7.903 7.897 7.893 7.890 7.885 7.883 7.882 7.869 7.867 7.866 7.826 7.825 7.823 7.822 7.811 7.810 7.808 7.807 7.715 7.712 7.596 7.593 7.582 7.580 7.579 7.577 7.566 7.563 7.520 7.509 7.506 7.503 7.497 7.490 7.489 7.485 7.299 7.297 7.284 7.282 7.269 7.267 7.132 7.130 7.114 7.113 7.096 7.091 7.078 7.074 6.394 6.389 6.383 4.762
4.05 1.00 1.03 1.04 2.18 1.11 1.07 1.02 1.01 0.97
8.0 7.5 7.0 6.5 6.0 5.5 5.0 4.5 4.0 3.5 3.0 2.5 2.0 1.5 1.0 0.5 0.0

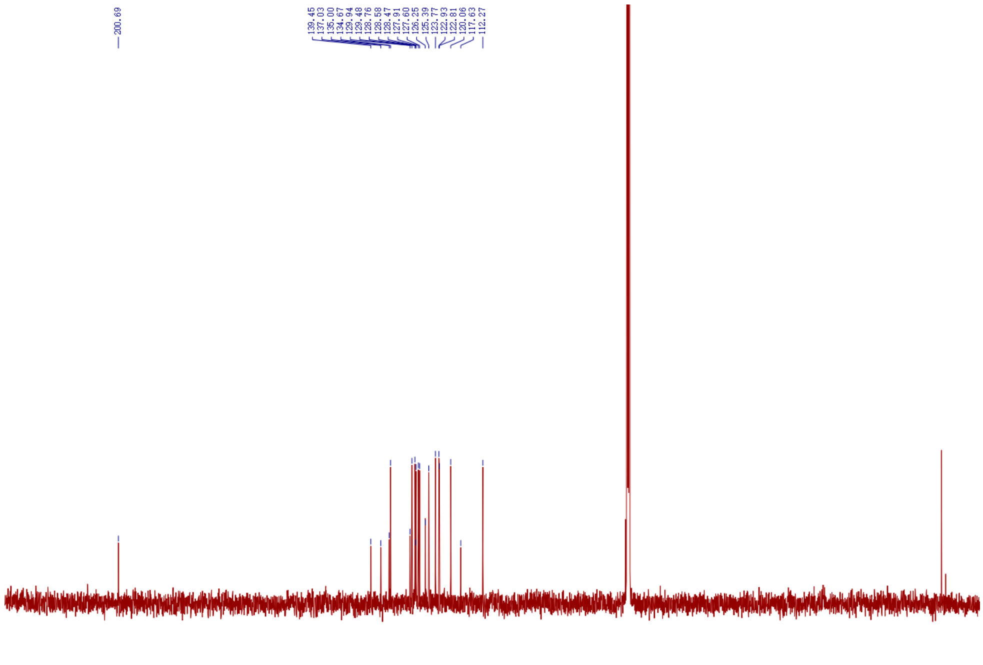
200.69
139.45 137.03 135.00 134.67 129.94 129.48 128.76 128.58 128.47 127.91 127.60 126.25 125.39 123.77 122.93 122.81 120.06 117.63 112.27
220 210 200 190 180 170 160 150 140 130 120 110 100 90 80 70 60 50 40 30 20 10 0

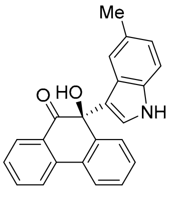
Me
O
HO
NH

(*R*)-4f

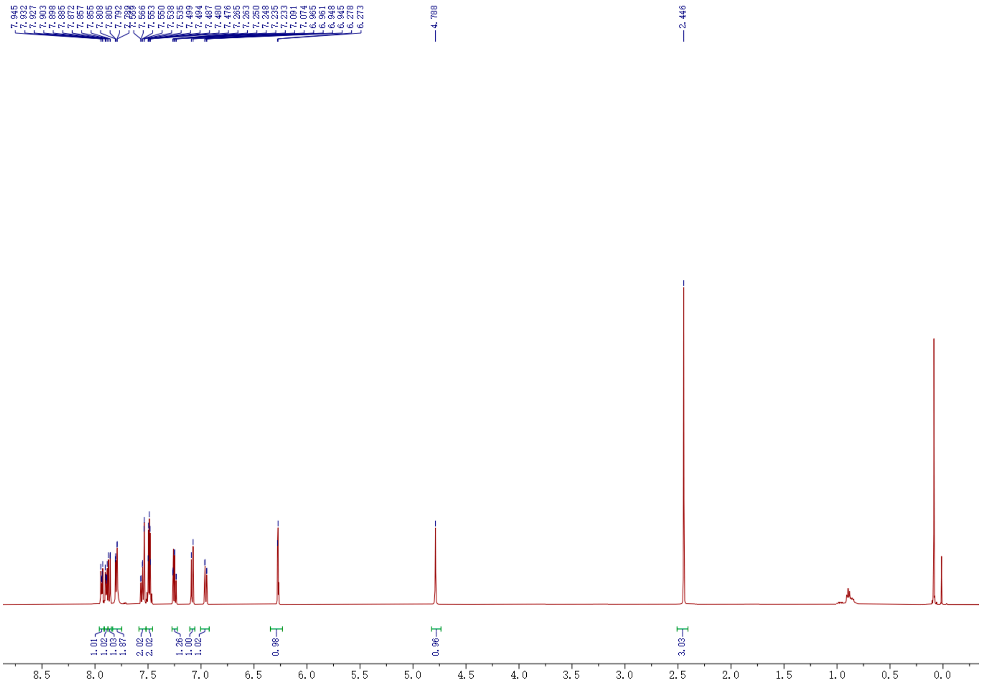
7.945
7.932
7.927
7.903
7.898
7.885
7.872
7.857
7.855
7.808
7.805
7.792
7.566
7.553
7.550
7.538
7.535
7.499
7.494
7.487
7.480
7.476
7.265
7.263
7.250
7.248
7.235
7.233
7.091
7.074
6.965
6.961
6.948
6.945
6.278
6.273
4.788
2.446
1.01
1.02
1.03
1.87
2.02
2.02
1.26
1.00
1.02
0.98
0.96
3.03
8.5
8.0
7.5
7.0
6.5
6.0
5.5
5.0
4.5
4.0
3.5
3.0
2.5
2.0
1.5
1.0
0.5
0.0

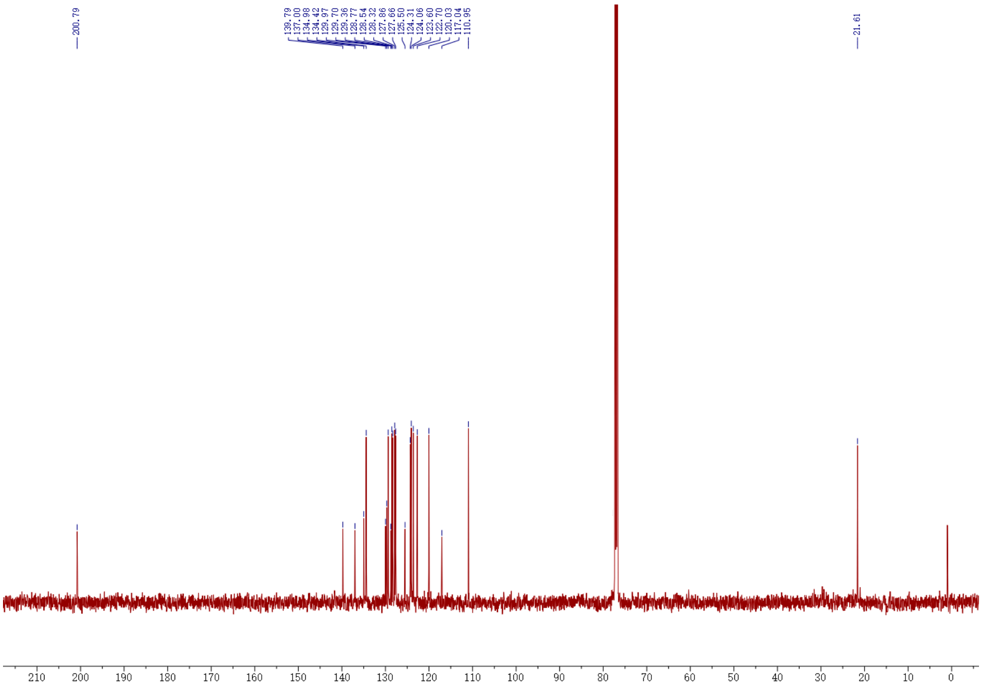
200.79
139.79
137.00
134.98
134.42
129.97
129.70
129.36
128.77
128.54
128.32
127.86
127.66
125.50
124.31
124.06
123.60
122.70
120.03
117.04
110.95
21.61
210
200
190
180
170
160
150
140
130
120
110
100
90
80
70
60
50
40
30
20
10
0

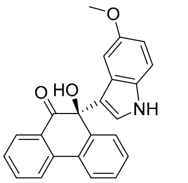

(*R*)-4g

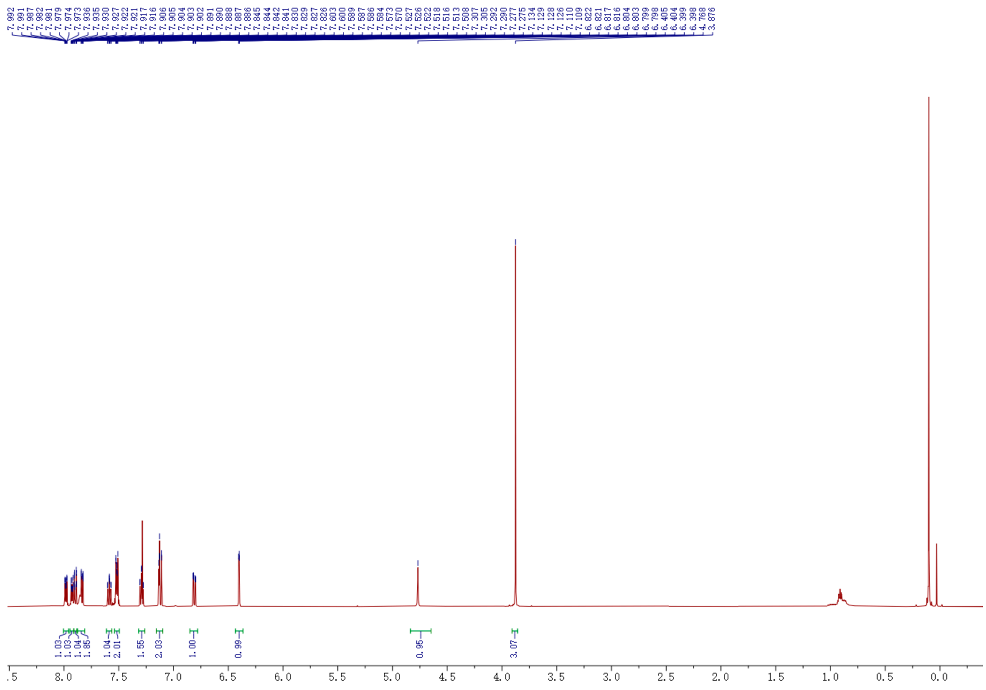
7.992 7.991 7.987 7.982 7.981 7.979 7.974 7.973 7.936 7.935 7.930 7.927 7.922 7.921 7.917 7.916 7.906 7.905 7.904 7.903 7.902 7.891 7.890 7.888 7.887 7.886 7.845 7.844 7.842 7.841 7.830 7.829 7.827 7.826 7.603 7.600 7.589 7.587 7.586 7.584 7.573 7.570 7.527 7.526 7.522 7.518 7.516 7.513 7.508 7.307 7.305 7.292 7.290 7.277 7.275 7.134 7.129 7.128 7.126 7.110 7.109 6.822 6.821 6.817 6.816 6.804 6.803 6.799 6.798 6.405 6.404 6.399 6.398 4.768 3.876
1.03
1.03
1.04
1.85
1.04
2.01
1.55
2.03
1.00
0.99
0.95
3.07
.5 8.0 7.5 7.0 6.5 6.0 5.5 5.0 4.5 4.0 3.5 3.0 2.5 2.0 1.5 1.0 0.5 0.0

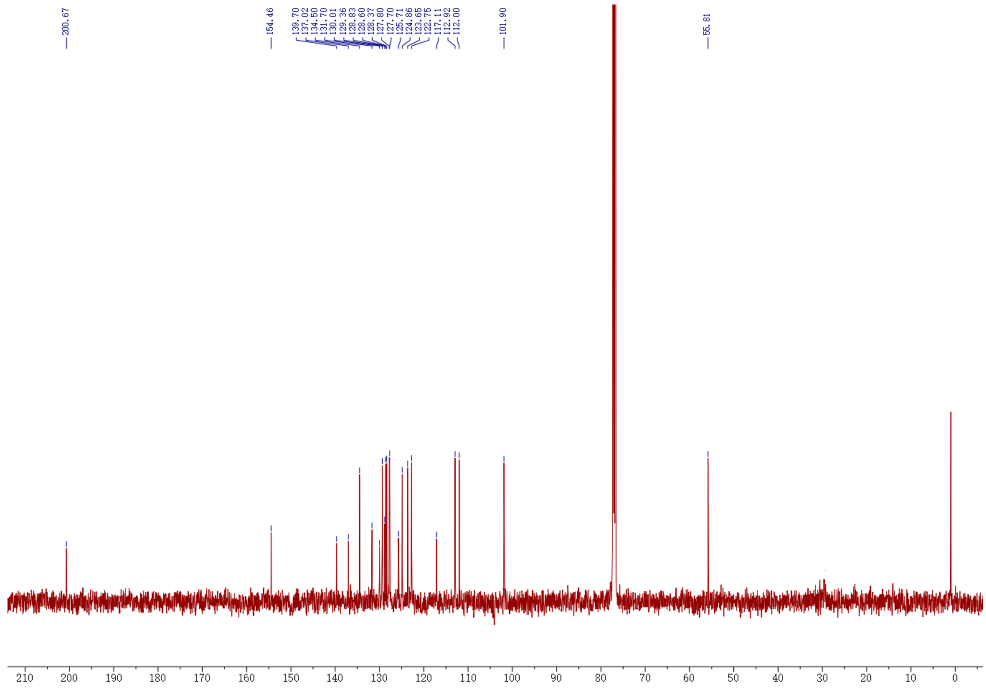
200.67
154.46
139.70 137.02 134.50 131.70 130.01 129.36 128.83 128.60 128.37 127.80 127.70 125.71 124.86 123.65 122.75 117.11 112.92 112.00
101.90
55.81
210 200 190 180 170 160 150 140 130 120 110 100 90 80 70 60 50 40 30 20 10 0

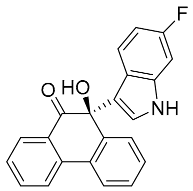
F
O
HO
NH

(*R*)-4i

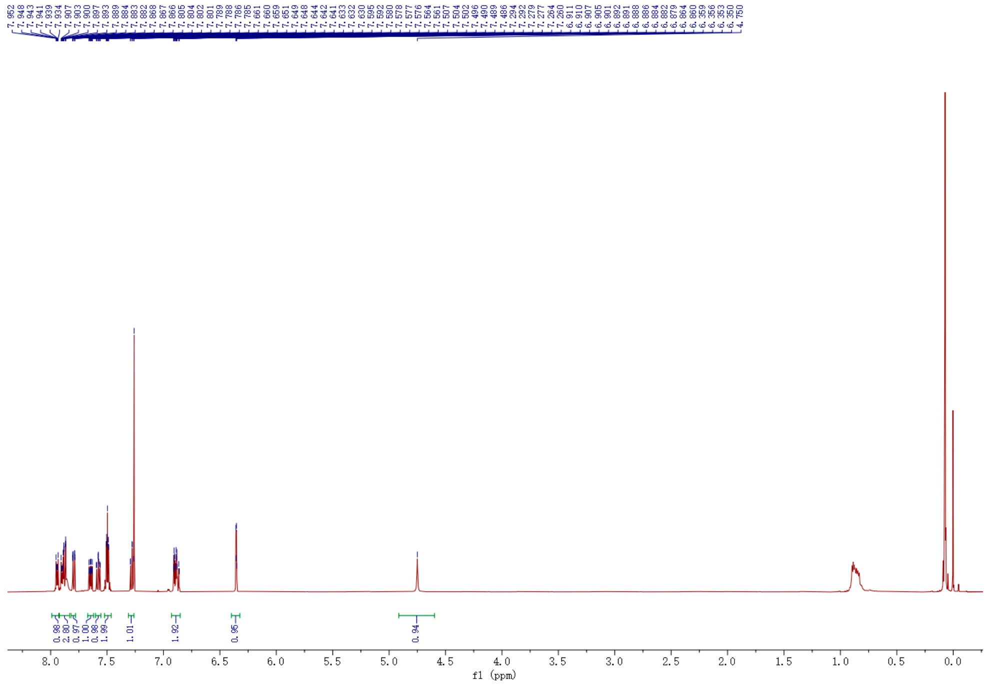
7.952
7.948
7.943
7.941
7.939
7.934
7.907
7.903
7.900
7.897
7.893
7.889
7.884
7.883
7.882
7.868
7.867
7.866
7.805
7.804
7.802
7.801
7.789
7.788
7.786
7.785
7.661
7.660
7.659
7.651
7.649
7.648
7.644
7.642
7.641
7.633
7.632
7.630
7.595
7.592
7.580
7.578
7.577
7.576
7.564
7.561
7.507
7.504
7.502
7.496
7.490
7.489
7.486
7.294
7.292
7.279
7.277
7.264
7.260
6.911
6.910
6.907
6.905
6.901
6.892
6.891
6.888
6.886
6.884
6.882
6.879
6.864
6.860
6.359
6.356
6.353
6.350
4.750
0.98
2.80
0.97
1.00
0.98
1.99
1.01
1.92
0.95
0.94
8.0
7.5
7.0
6.5
6.0
5.5
5.0
4.5
4.0
3.5
3.0
2.5
2.0
1.5
1.0
0.5
0.0
f1 (ppm)

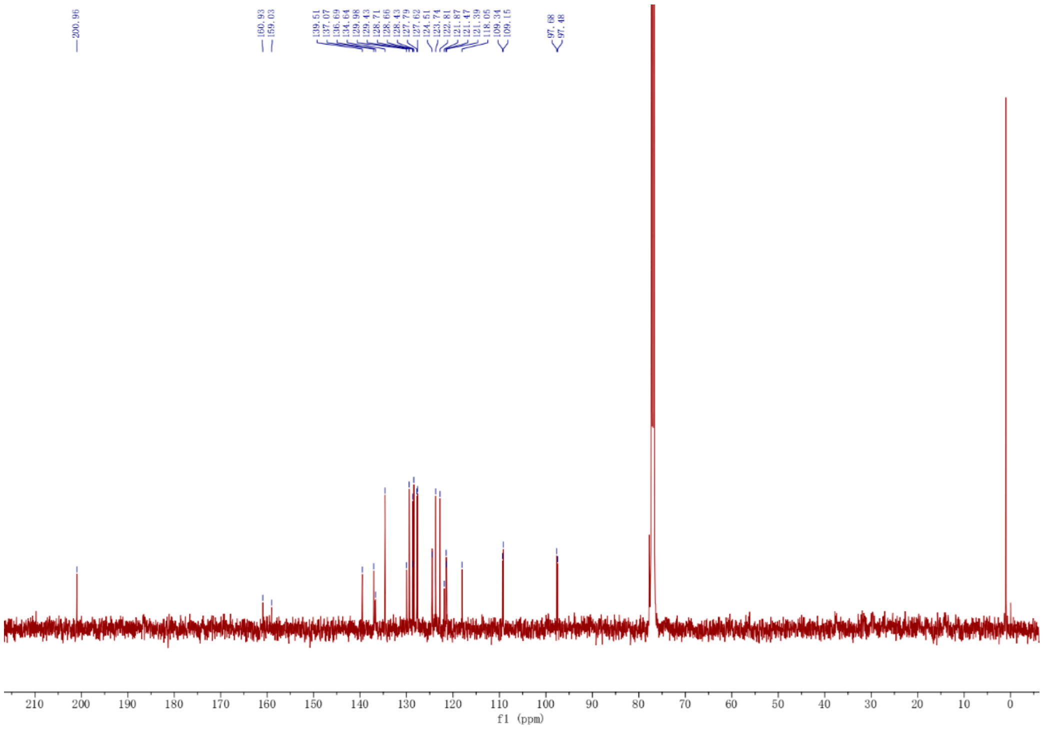
200.96
160.93
159.03
139.51
137.07
136.69
134.64
129.98
129.43
128.71
128.66
128.43
127.79
127.62
124.51
123.74
122.81
121.87
121.47
121.39
118.05
109.34
109.15
97.68
97.48
210
200
190
180
170
160
150
140
130
120
110
100
90
80
70
60
50
40
30
20
10
0
f1 (ppm)

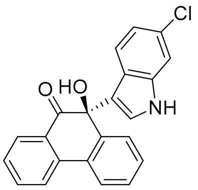
Cl
O
HO
NH

(*R*)-4j

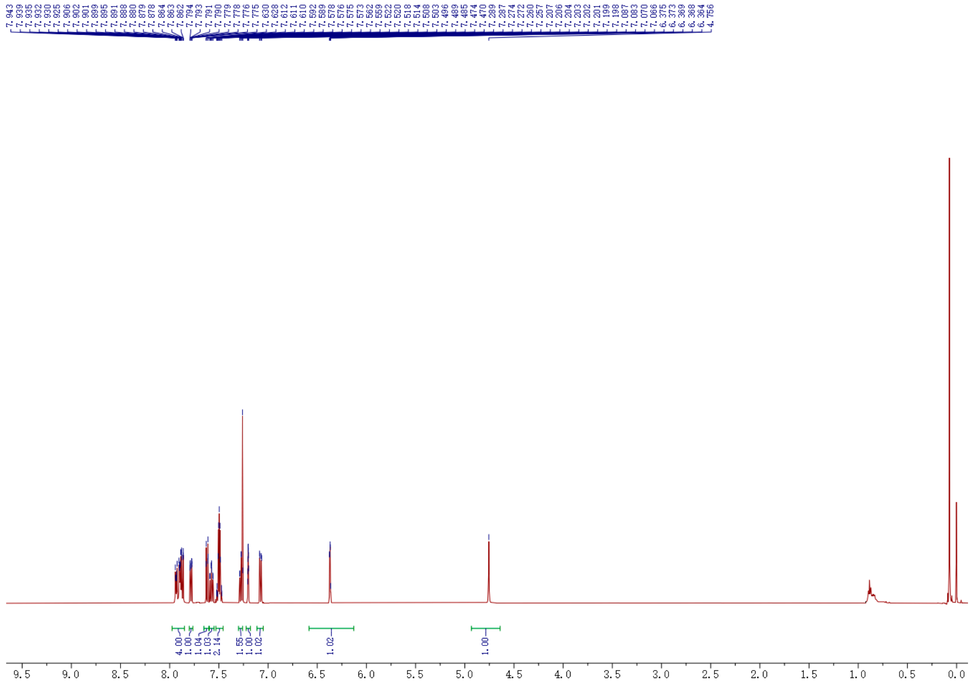
7.943 7.939 7.935 7.932 7.930 7.925 7.906 7.902 7.901 7.899 7.895 7.891 7.888 7.880 7.879 7.878 7.864 7.863 7.862 7.794 7.793 7.791 7.790 7.779 7.778 7.776 7.775 7.630 7.628 7.612 7.611 7.610 7.592 7.589 7.578 7.576 7.575 7.573 7.562 7.559 7.522 7.520 7.518 7.514 7.508 7.503 7.496 7.489 7.485 7.474 7.470 7.289 7.287 7.274 7.272 7.260 7.257 7.237 7.206 7.204 7.203 7.202 7.201 7.199 7.198 7.087 7.083 7.070 7.066 6.375 6.373 6.369 6.368 6.364 4.756
4.00
1.00
1.04
1.03
2.14
1.55
1.00
1.02
1.02
1.00
9.5
9.0
8.5
8.0
7.5
7.0
6.5
6.0
5.5
5.0
4.5
4.0
3.5
3.0
2.5
2.0
1.5
1.0
0.5
0.0

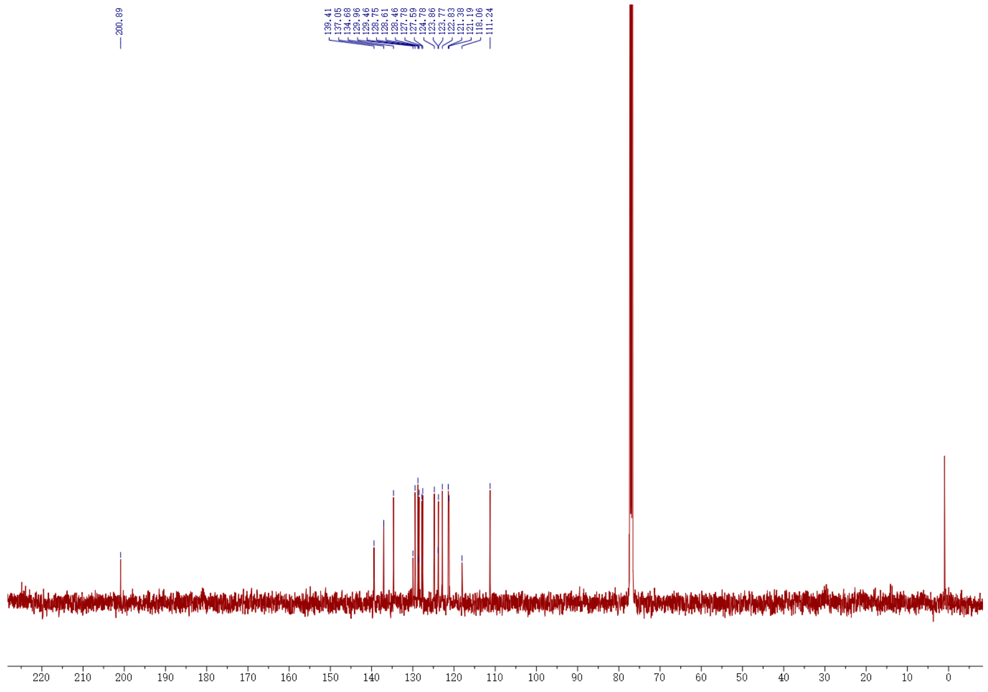
200.89
139.41 137.05 134.68 129.96 129.46 128.75 128.61 128.46 127.78 127.59 124.78 123.86 123.77 122.83 121.38 121.19 118.06 111.24
220
210
200
190
180
170
160
150
140
130
120
110
100
90
80
70
60
50
40
30
20
10
0

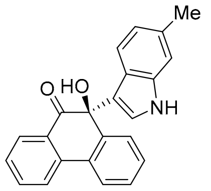

(*R*)-4k

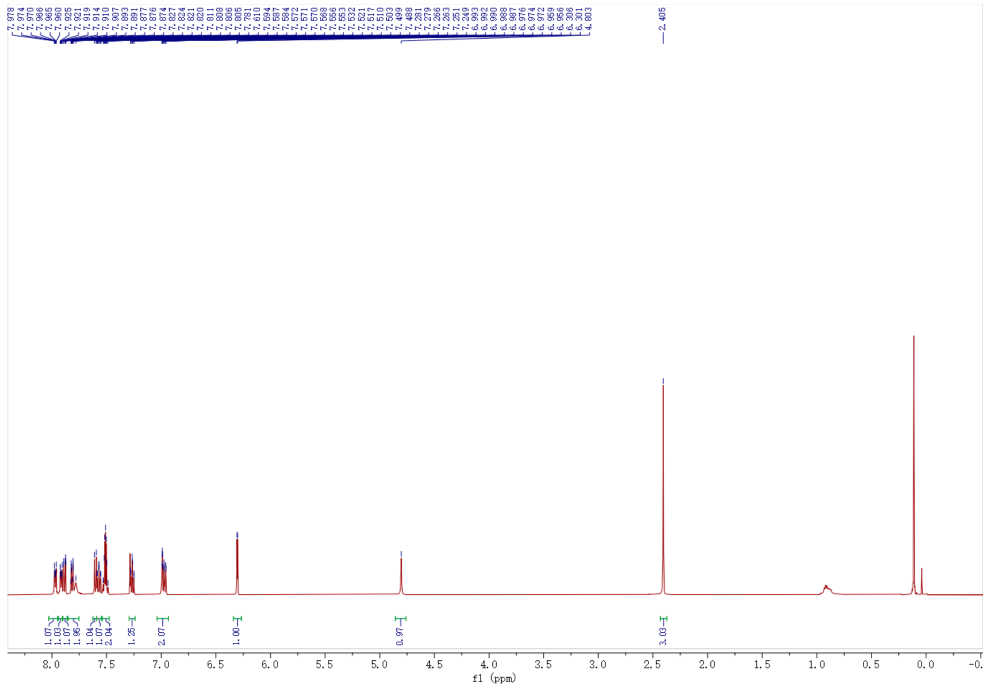

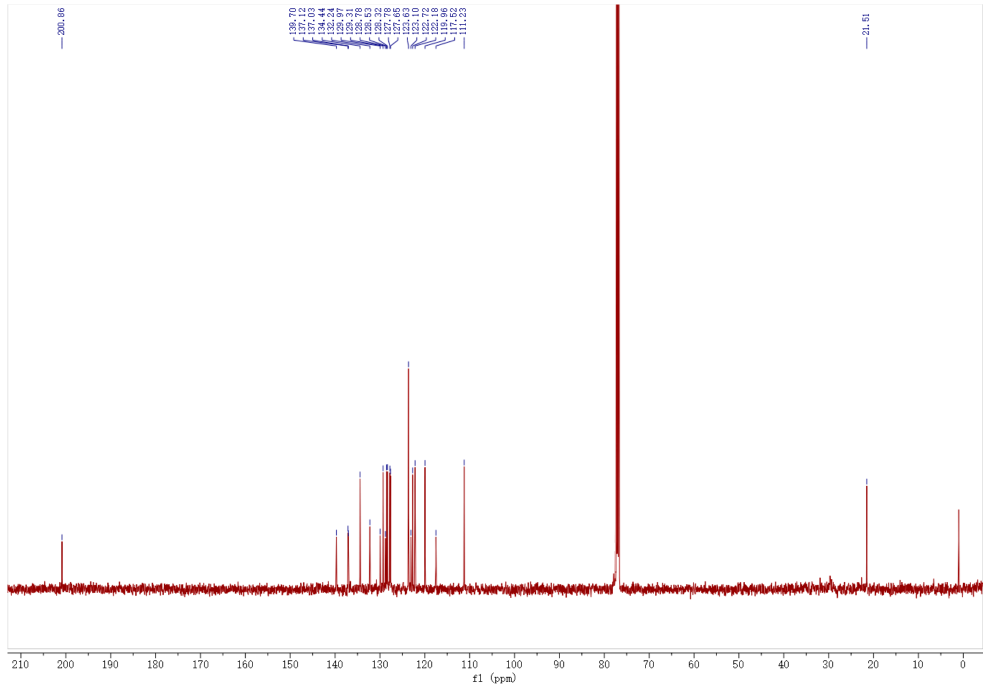

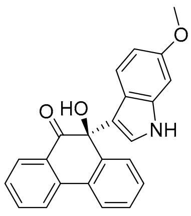
O
HO
NH

(*R*)-4l

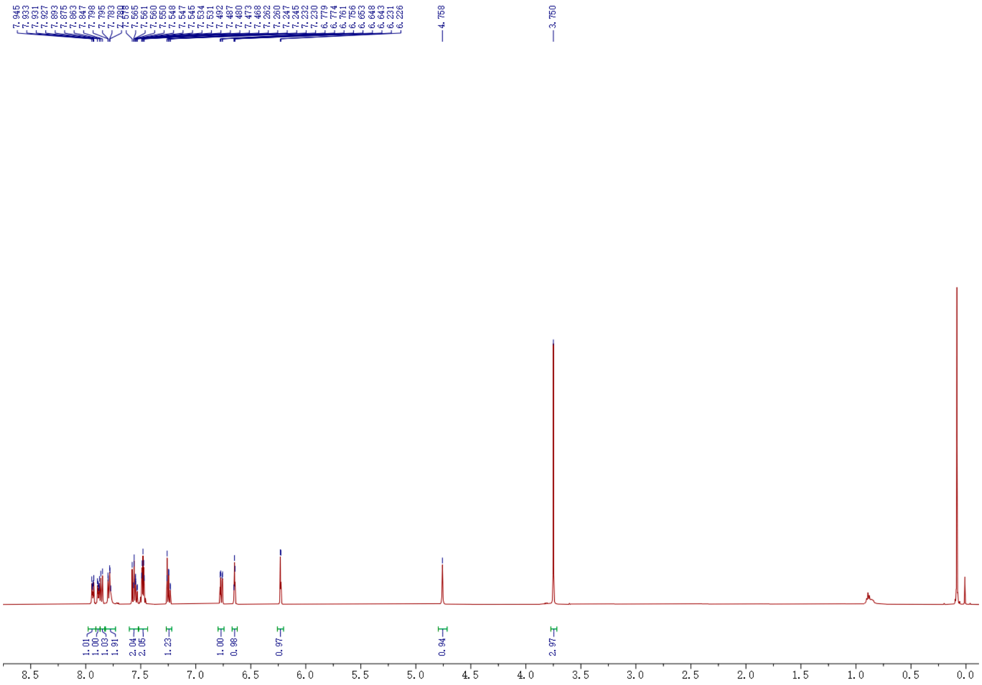
4.758
3.750

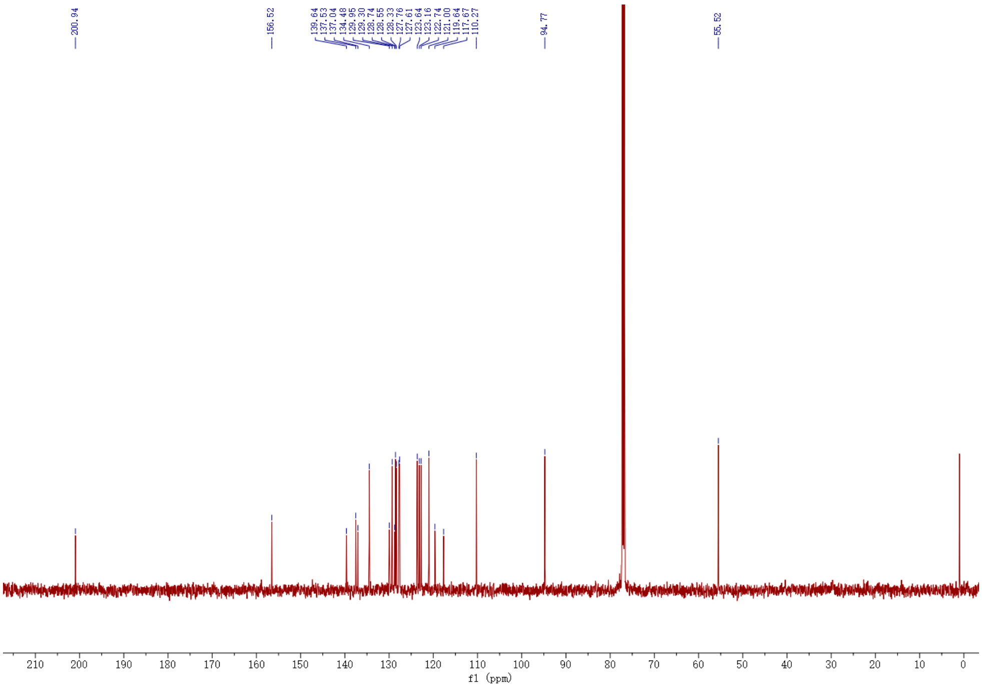
200.94
156.52
139.64
137.53
137.04
134.48
129.95
129.30
128.74
128.55
128.33
127.76
127.61
123.64
123.16
122.74
121.00
119.64
117.67
110.27
94.77
55.52
f1 (ppm)

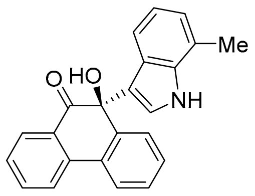

(*R*)-4o

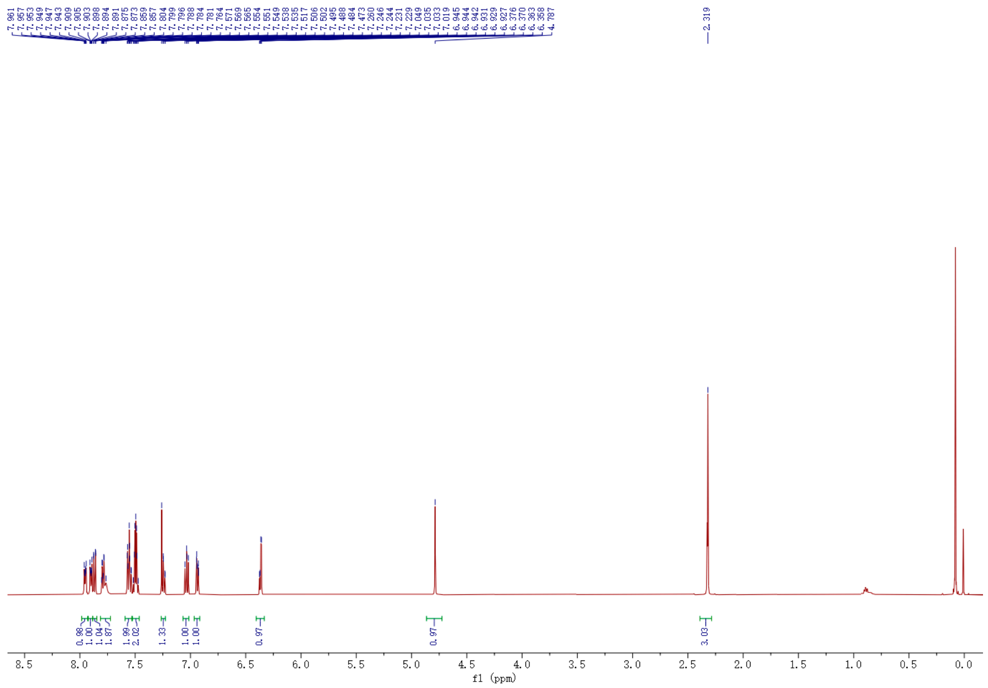

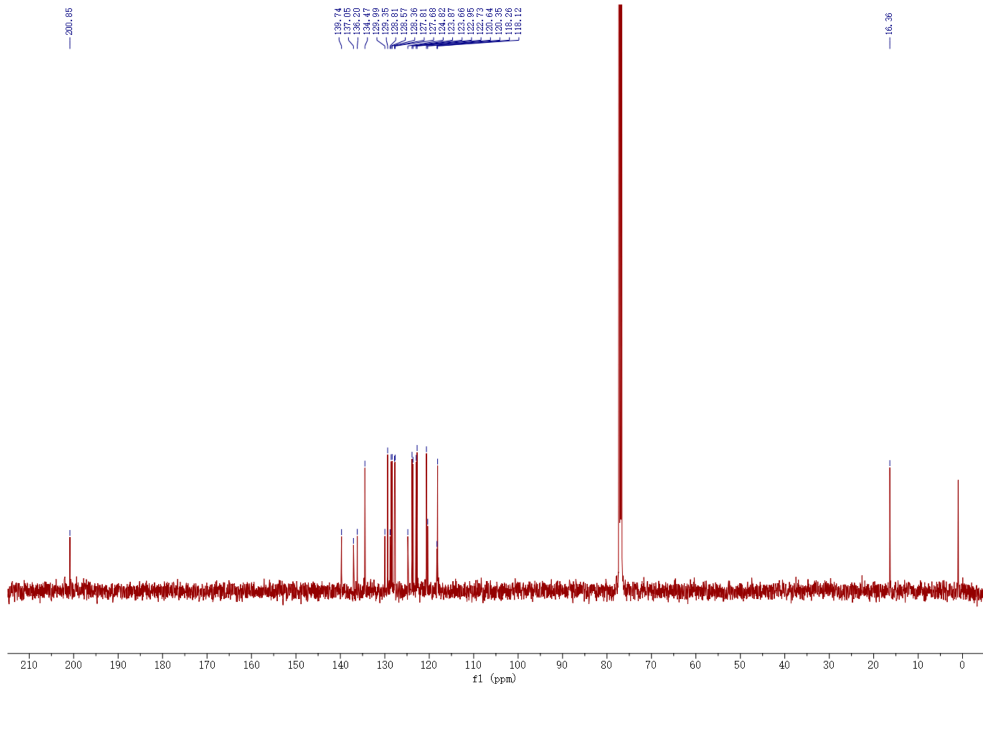

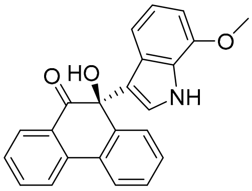

(*R*)-4p

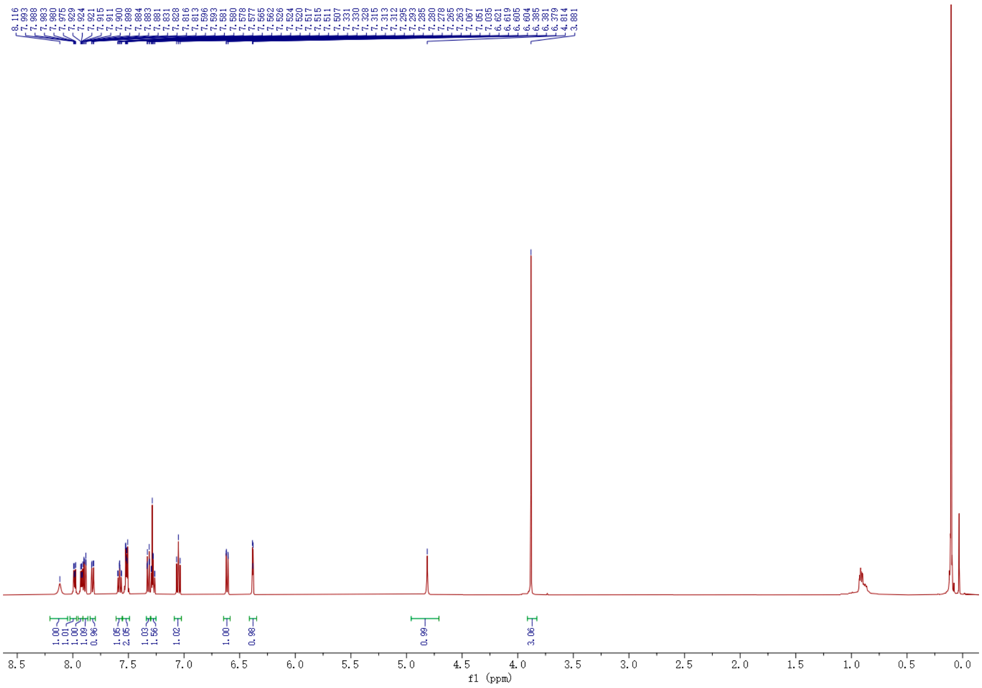
f1 (ppm)

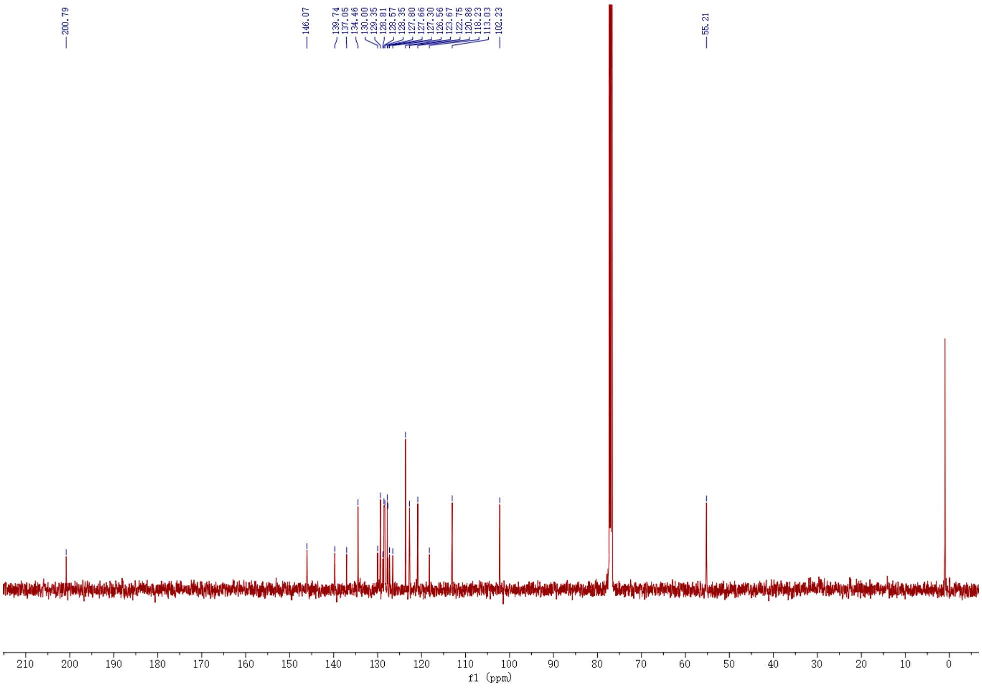
200.79
146.07
139.74
137.05
134.46
130.00
129.35
128.81
128.57
128.35
127.80
127.66
127.30
126.56
123.67
122.75
120.86
118.23
113.03
102.23
55.21
f1 (ppm)

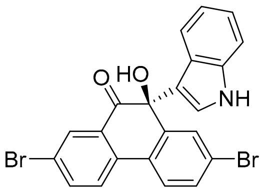
O
HO
NH
Br
Br

(*R*)-4q

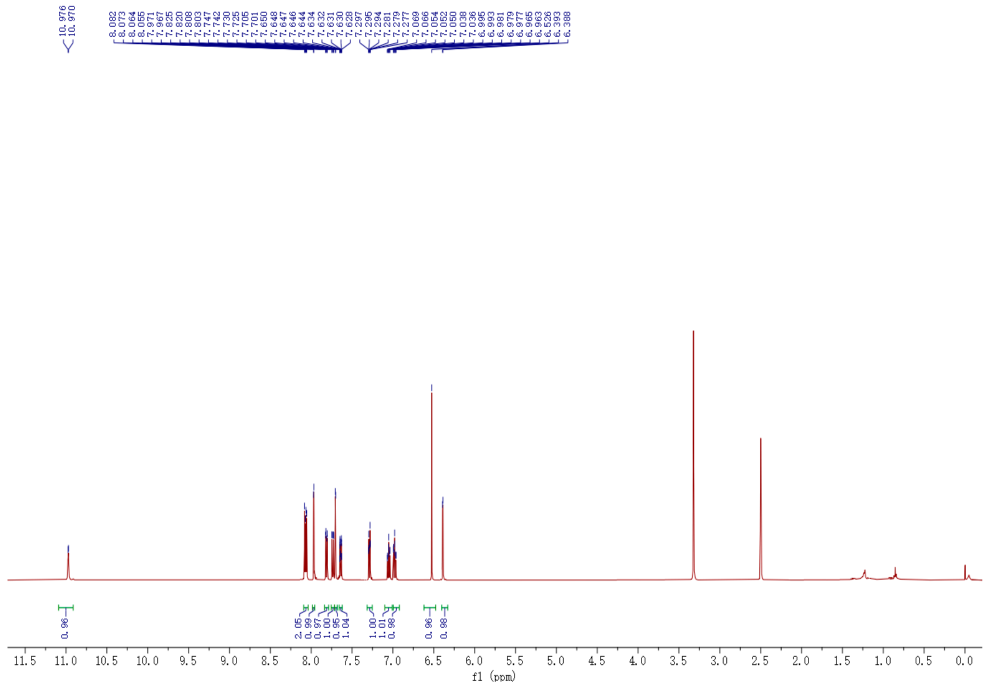
10.976
10.970
8.082
8.073
8.064
8.055
7.971
7.967
7.825
7.820
7.808
7.803
7.747
7.742
7.730
7.725
7.705
7.701
7.650
7.648
7.647
7.646
7.644
7.634
7.632
7.631
7.630
7.628
7.297
7.295
7.294
7.281
7.279
7.277
7.069
7.066
7.054
7.052
7.050
7.038
7.036
6.995
6.993
6.981
6.979
6.977
6.965
6.963
6.526
6.393
6.388
0.96
2.05
0.99
0.97
1.00
0.95
1.04
1.00
1.01
0.98
0.96
0.98
11.5
11.0
10.5
10.0
9.5
9.0
8.5
8.0
7.5
7.0
6.5
6.0
5.5
5.0
4.5
4.0
3.5
3.0
2.5
2.0
1.5
1.0
0.5
0.0
f1 (ppm)

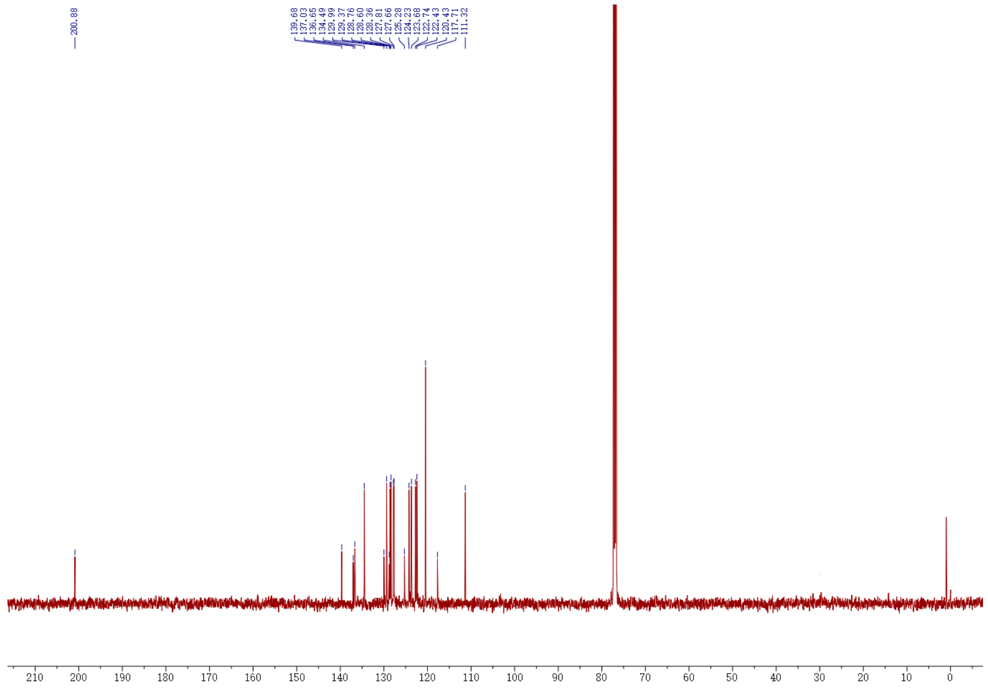
200.88
139.68
137.03
136.65
134.49
129.99
129.37
128.76
128.60
128.36
127.81
127.66
125.28
124.23
123.88
122.74
122.43
120.43
117.71
111.32
210
200
190
180
170
160
150
140
130
120
110
100
90
80
70
60
50
40
30
20
10
0

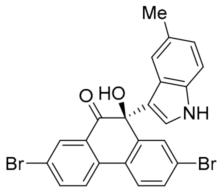
Me
O
HO
NH
Br
Br

(*R*)-4r

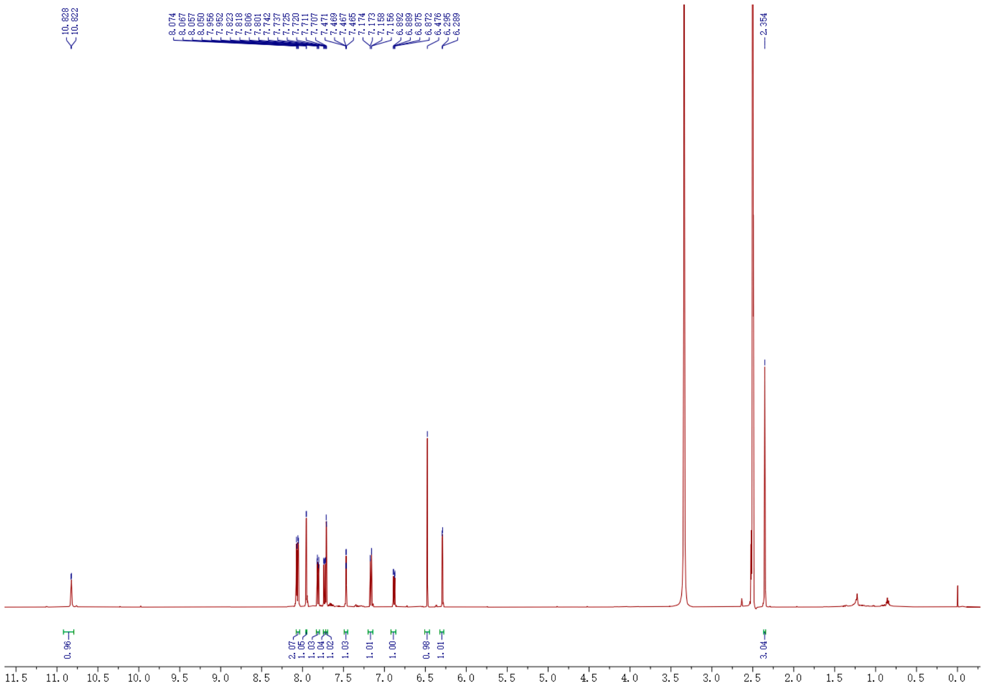
10.828
10.822
8.074
8.067
8.057
8.050
7.956
7.952
7.823
7.818
7.806
7.801
7.742
7.737
7.725
7.720
7.711
7.707
7.471
7.469
7.467
7.465
7.174
7.173
7.158
7.156
6.892
6.889
6.875
6.872
6.476
6.295
6.289
2.354
0.96
2.07
1.05
1.03
1.04
1.02
1.03
1.01
1.00
0.98
1.01
3.04
11.5
11.0
10.5
10.0
9.5
9.0
8.5
8.0
7.5
7.0
6.5
6.0
5.5
5.0
4.5
4.0
3.5
3.0
2.5
2.0
1.5
1.0
0.5
0.0

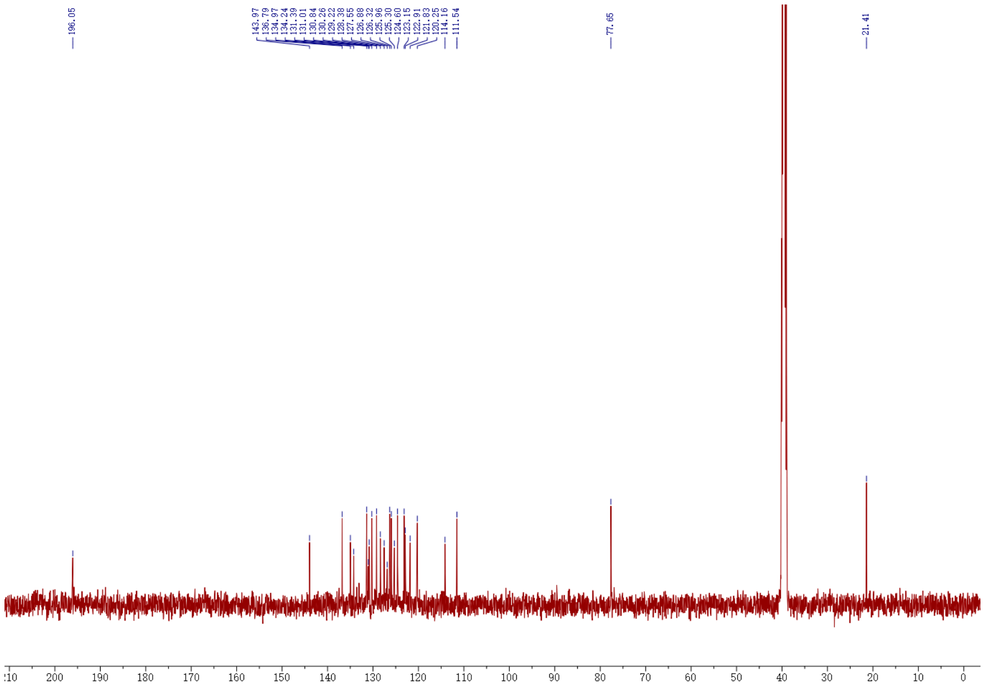
196.05
143.97
136.79
134.97
134.24
131.39
131.01
130.84
130.26
129.22
128.38
127.55
126.88
126.32
125.96
125.30
124.60
123.15
122.91
121.83
120.25
114.16
111.54
77.65
21.41
200
190
180
170
160
150
140
130
120
110
100
90
80
70
60
50
40
30
20
10
0

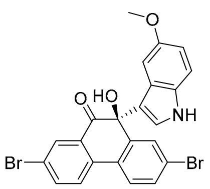
O
HO
NH
Br
Br

(*R*)-4s

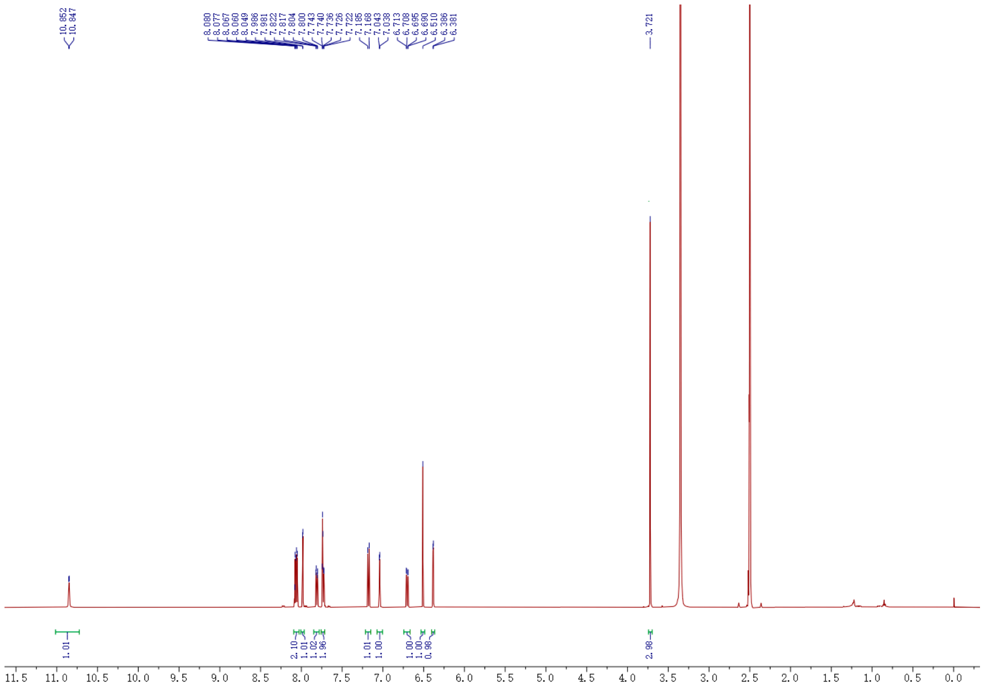
10.852
10.847
8.080
8.077
8.067
8.060
8.049
7.986
7.981
7.822
7.817
7.804
7.800
7.743
7.740
7.736
7.726
7.722
7.185
7.168
7.043
7.038
6.713
6.708
6.695
6.690
6.510
6.386
6.381
3.721
1.01
2.10
1.01
1.02
1.96
1.01
1.00
1.00
1.00
0.98
2.98
11.5 11.0 10.5 10.0 9.5 9.0 8.5 8.0 7.5 7.0 6.5 6.0 5.5 5.0 4.5 4.0 3.5 3.0 2.5 2.0 1.5 1.0 0.5 0.0

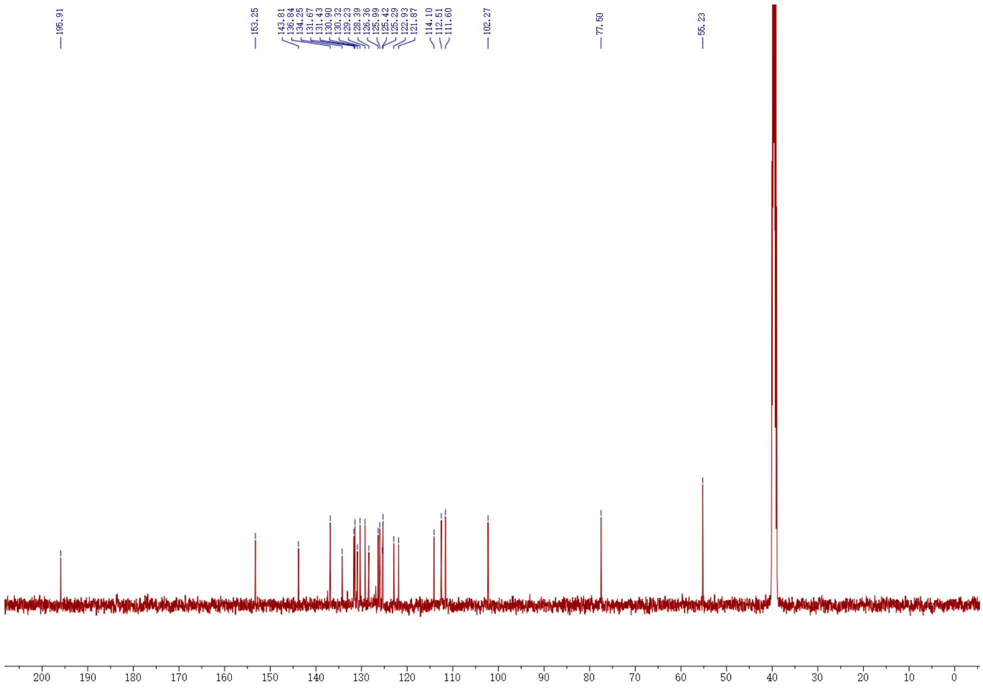
195.91
153.25
143.81
136.84
134.25
131.67
131.43
130.90
130.32
129.23
128.39
126.36
125.99
125.42
125.29
122.93
121.87
114.10
112.51
111.60
102.27
77.50
55.23
200 190 180 170 160 150 140 130 120 110 100 90 80 70 60 50 40 30 20 10 0

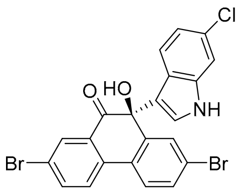

(*R*)-4t

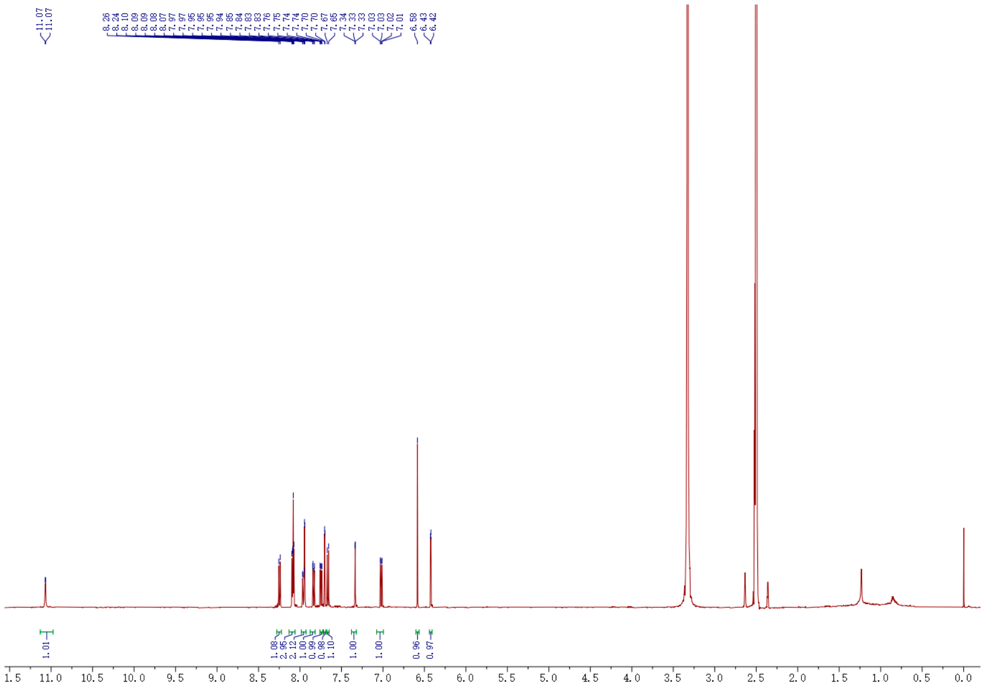

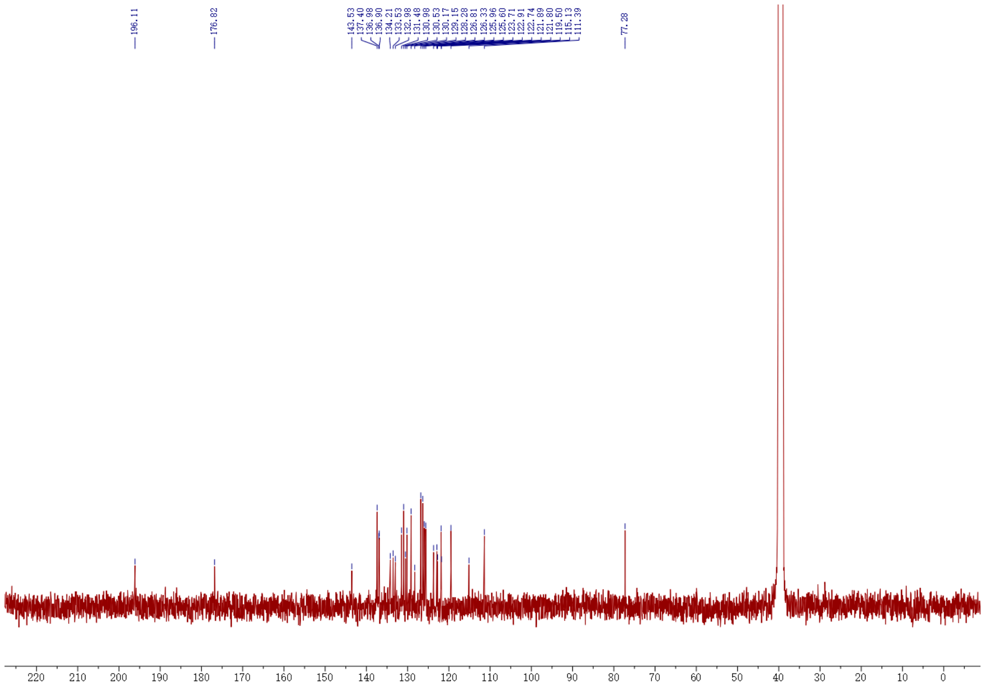

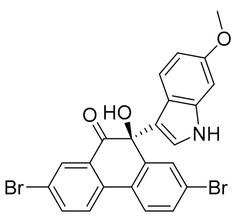
O
HO
NH
Br
Br

(*R*)-4u

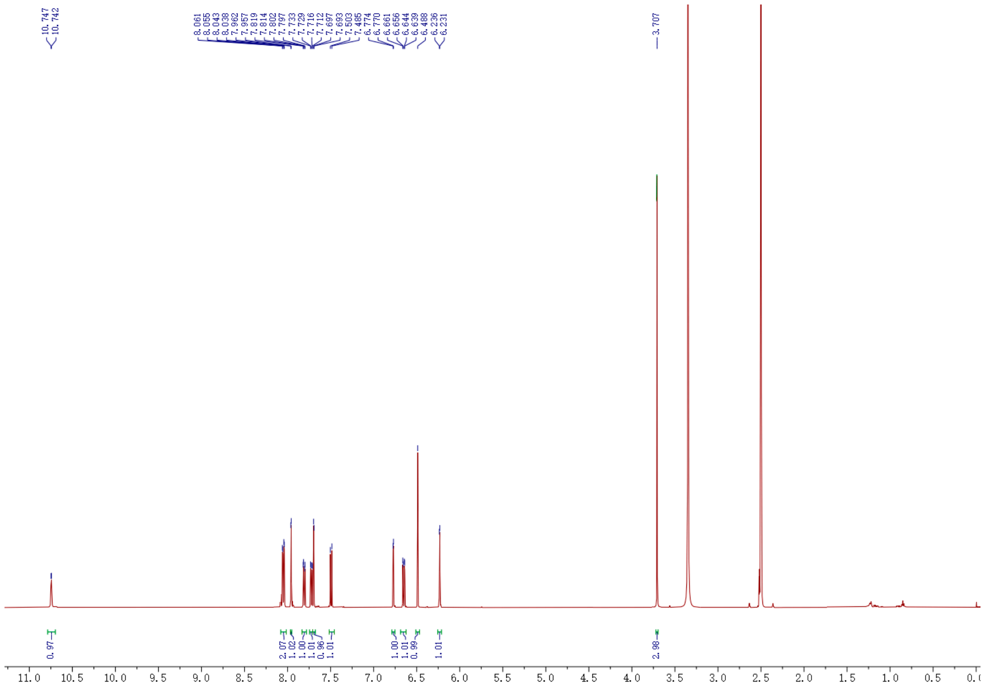
10.747
10.742
8.061
8.055
8.043
8.038
7.962
7.957
7.819
7.814
7.802
7.797
7.733
7.729
7.716
7.712
7.697
7.693
7.503
7.485
6.774
6.770
6.661
6.656
6.644
6.639
6.488
6.236
6.231
3.707
0.97
2.07
1.02
1.00
1.01
0.96
1.01
1.00
1.01
0.99
1.01
2.98
11.0 10.5 10.0 9.5 9.0 8.5 8.0 7.5 7.0 6.5 6.0 5.5 5.0 4.5 4.0 3.5 3.0 2.5 2.0 1.5 1.0 0.5

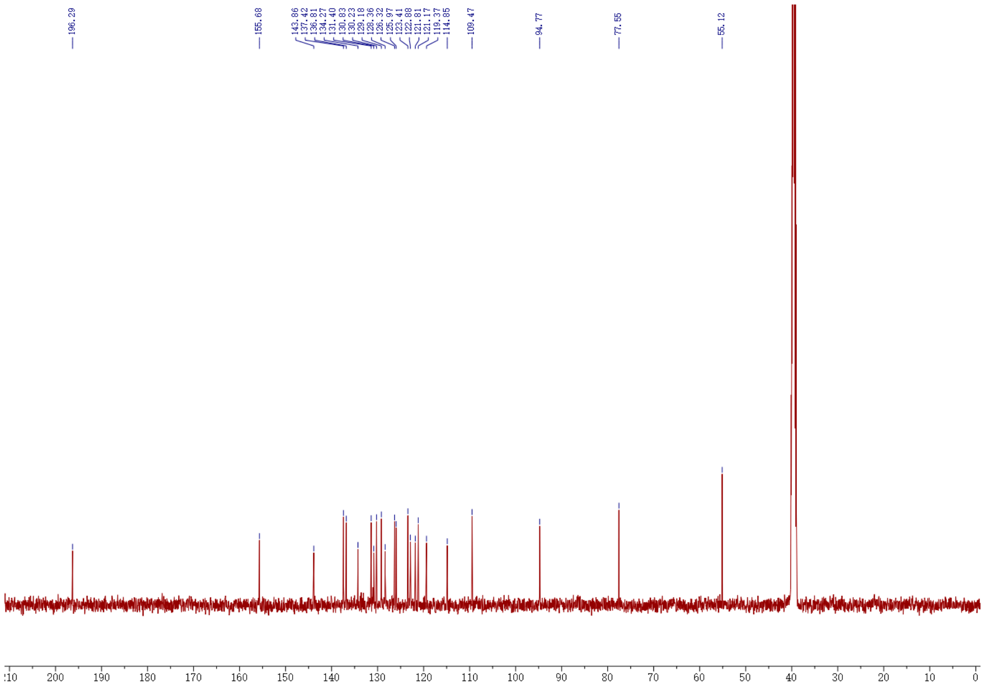
196.29
155.68
143.86
137.42
136.81
134.27
131.40
130.83
130.23
129.18
128.36
126.32
125.97
123.41
122.88
121.81
121.17
119.37
114.85
109.47
94.77
77.55
55.12
200 190 180 170 160 150 140 130 120 110 100 90 80 70 60 50 40 30 20 10 0

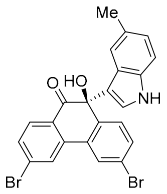
Me
O
HO
NH
Br
Br

(*R*)-4v

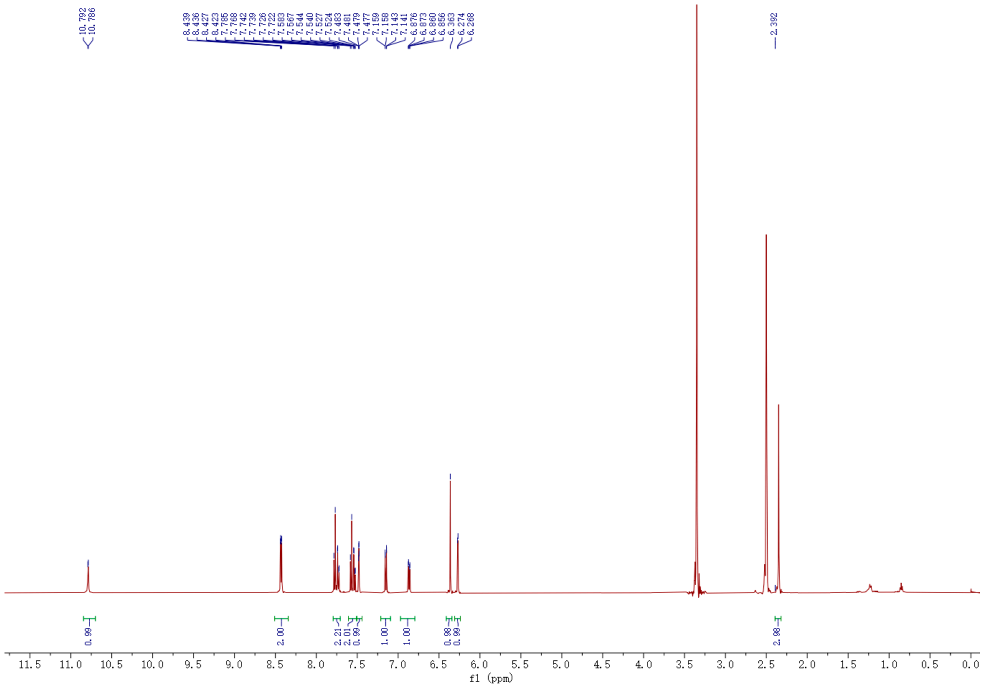
10.792
10.786
8.439
8.436
8.427
8.423
7.785
7.768
7.742
7.739
7.726
7.722
7.583
7.567
7.544
7.540
7.527
7.524
7.483
7.481
7.479
7.477
7.159
7.158
7.143
7.141
6.876
6.873
6.860
6.856
6.353
6.274
6.268
2.392
0.99
2.00
2.21
2.01
0.99
1.00
1.00
0.98
0.99
2.98
11.5 11.0 10.5 10.0 9.5 9.0 8.5 8.0 7.5 7.0 6.5 6.0 5.5 5.0 4.5 4.0 3.5 3.0 2.5 2.0 1.5 1.0 0.5 0.0
f1 (ppm)

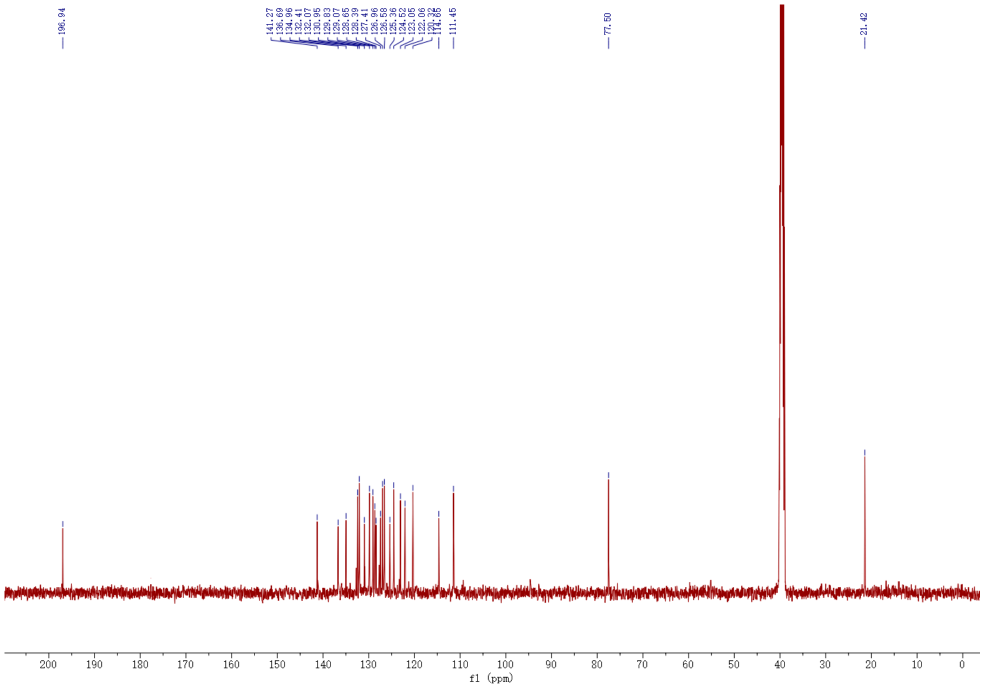
196.94
141.27
136.69
134.96
132.41
132.07
130.95
129.83
129.07
128.65
128.39
127.41
126.96
126.58
125.36
124.52
123.05
122.06
120.32
114.45
111.45
77.50
21.42
200 190 180 170 160 150 140 130 120 110 100 90 80 70 60 50 40 30 20 10 0
f1 (ppm)

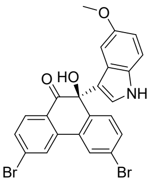
O
HO
NH
Br
Br

(*R*)-4w

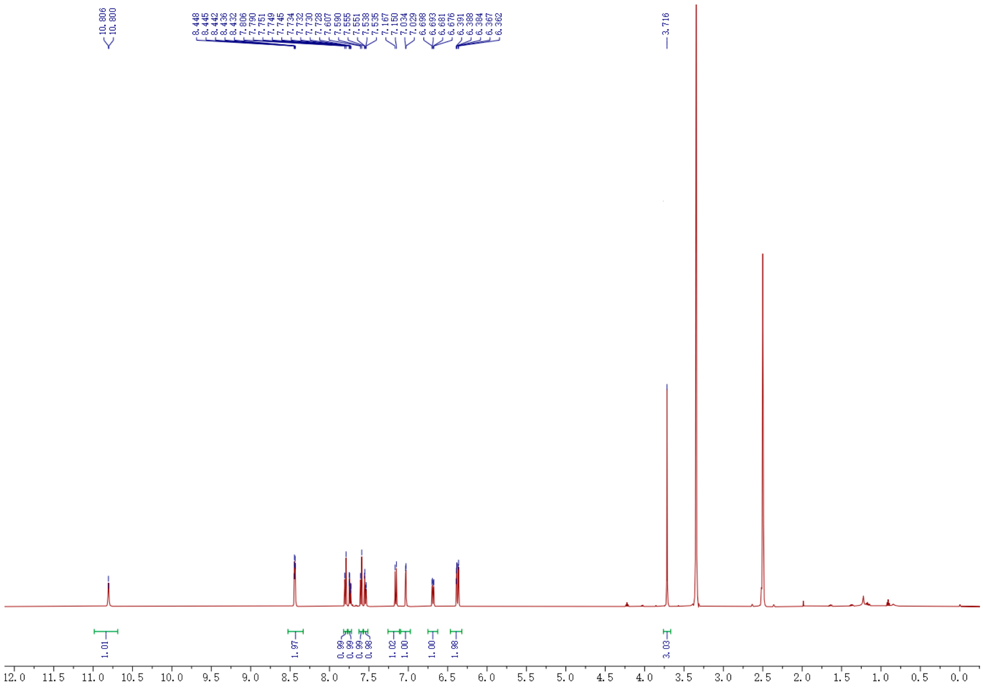
10.806
10.800
8.448
8.445
8.442
8.436
8.432
7.806
7.790
7.751
7.749
7.745
7.734
7.732
7.730
7.728
7.607
7.590
7.555
7.551
7.538
7.535
7.167
7.150
7.034
7.029
6.698
6.693
6.681
6.676
6.391
6.388
6.384
6.367
6.362
3.716
1.01
1.97
0.99
0.99
0.99
0.98
1.02
1.00
1.00
1.98
3.03
12.0 11.5 11.0 10.5 10.0 9.5 9.0 8.5 8.0 7.5 7.0 6.5 6.0 5.5 5.0 4.5 4.0 3.5 3.0 2.5 2.0 1.5 1.0 0.5 0.0

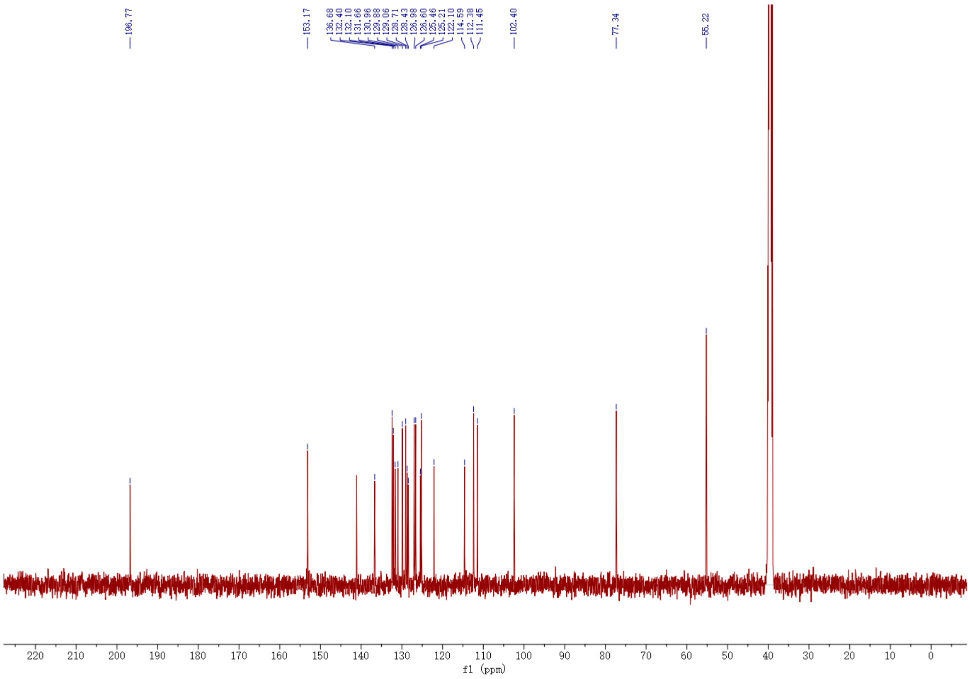
196.77
153.17
136.68
132.40
132.10
131.66
130.86
129.88
129.06
128.71
128.43
126.98
126.60
125.46
125.21
122.10
114.59
112.38
111.45
102.40
77.34
55.22
220 210 200 190 180 170 160 150 140 130 120 110 100 90 80 70 60 50 40 30 20 10 0
f1 (ppm)

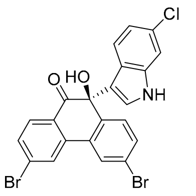
Cl
O
HO
NH
Br
Br

(*R*)-4x

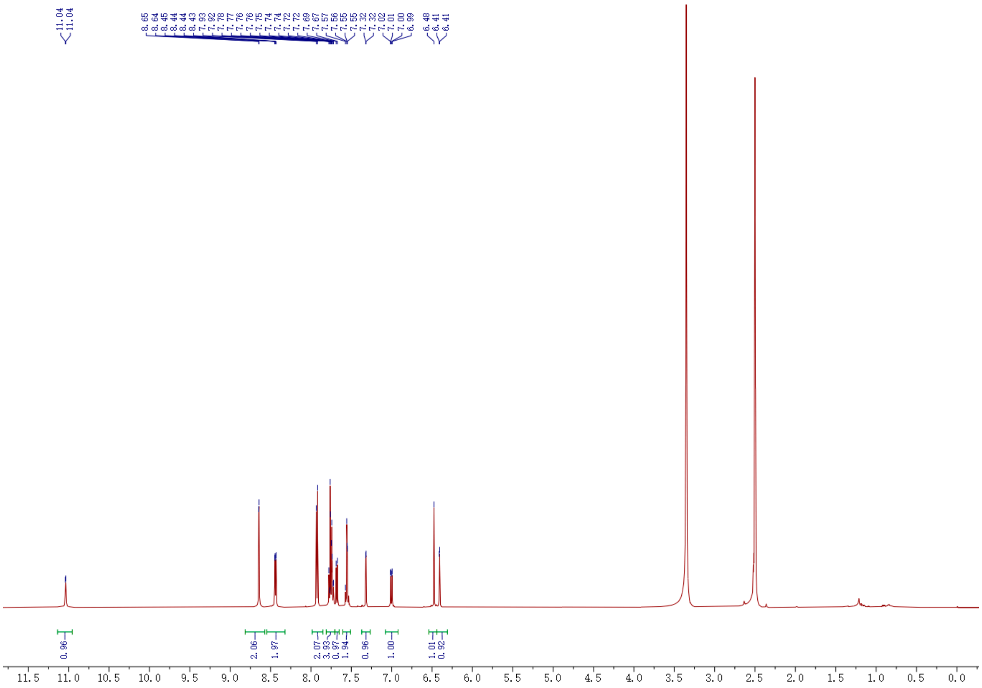
11.04
11.04
8.65
8.64
8.45
8.44
8.44
8.43
7.93
7.92
7.78
7.77
7.76
7.76
7.75
7.74
7.74
7.72
7.72
7.69
7.67
7.67
7.56
7.55
7.55
7.32
7.32
7.02
7.01
7.00
6.99
6.48
6.41
6.41
0.96
2.06
1.97
2.07
3.93
0.97
1.94
0.96
1.00
1.01
0.92
11.5
11.0
10.5
10.0
9.5
9.0
8.5
8.0
7.5
7.0
6.5
6.0
5.5
5.0
4.5
4.0
3.5
3.0
2.5
2.0
1.5
1.0
0.5
0.0

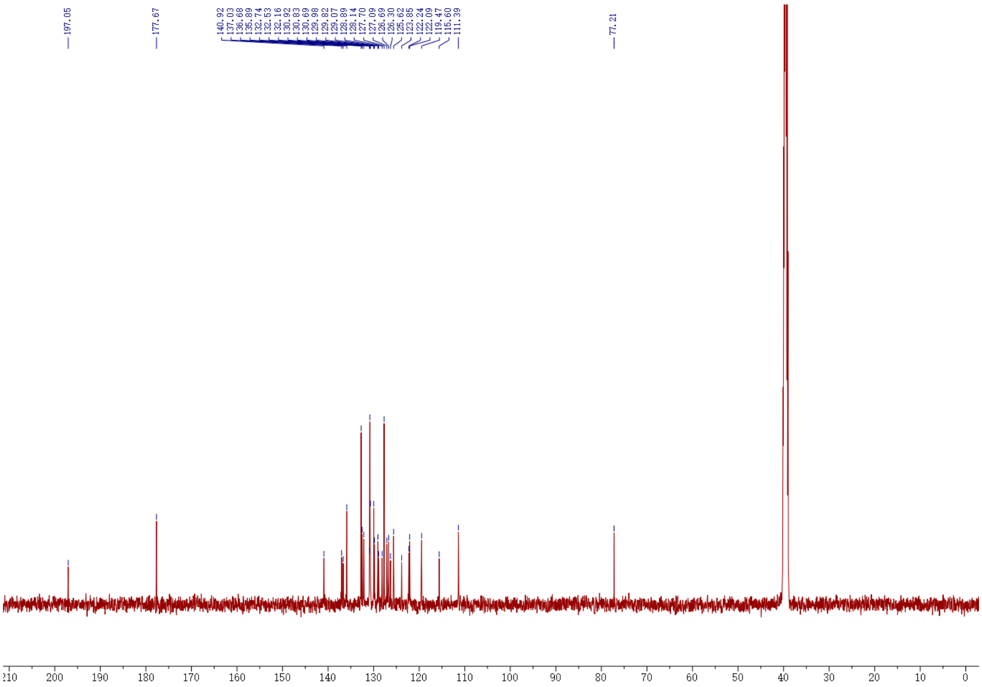
197.05
177.67
140.92
137.03
136.68
135.89
132.74
132.53
132.16
130.92
130.83
130.69
129.98
129.82
129.07
128.89
128.14
127.70
127.09
126.69
126.30
125.62
123.85
122.24
122.09
119.47
115.60
111.39
77.21
210
200
190
180
170
160
150
140
130
120
110
100
90
80
70
60
50
40
30
20
10
0

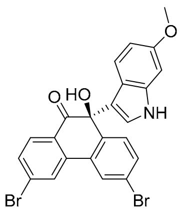
O
HO
O
NH
Br
Br

(*R*)-4y

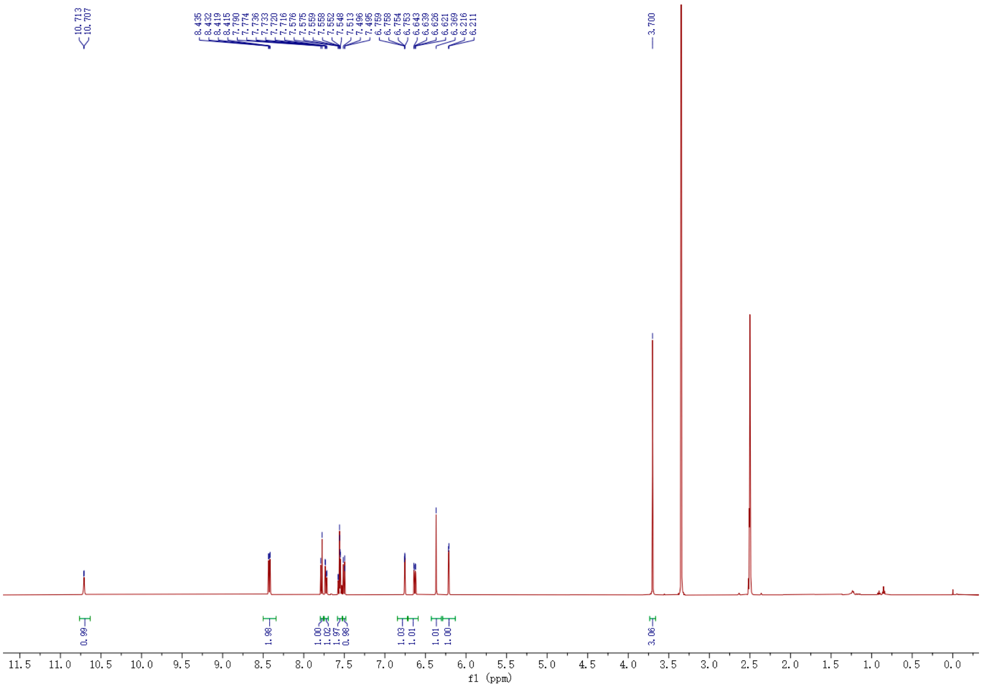
10.713
10.707
8.435
8.432
8.419
8.415
7.790
7.774
7.736
7.733
7.720
7.716
7.576
7.575
7.559
7.558
7.552
7.548
7.513
7.496
7.495
6.759
6.758
6.754
6.753
6.643
6.639
6.626
6.621
6.369
6.216
6.211
3.700
0.99
1.98
1.00
1.02
1.97
0.98
1.03
1.01
1.01
1.00
3.06
11.5 11.0 10.5 10.0 9.5 9.0 8.5 8.0 7.5 7.0 6.5 6.0 5.5 5.0 4.5 4.0 3.5 3.0 2.5 2.0 1.5 1.0 0.5 0.0
f1 (ppm)

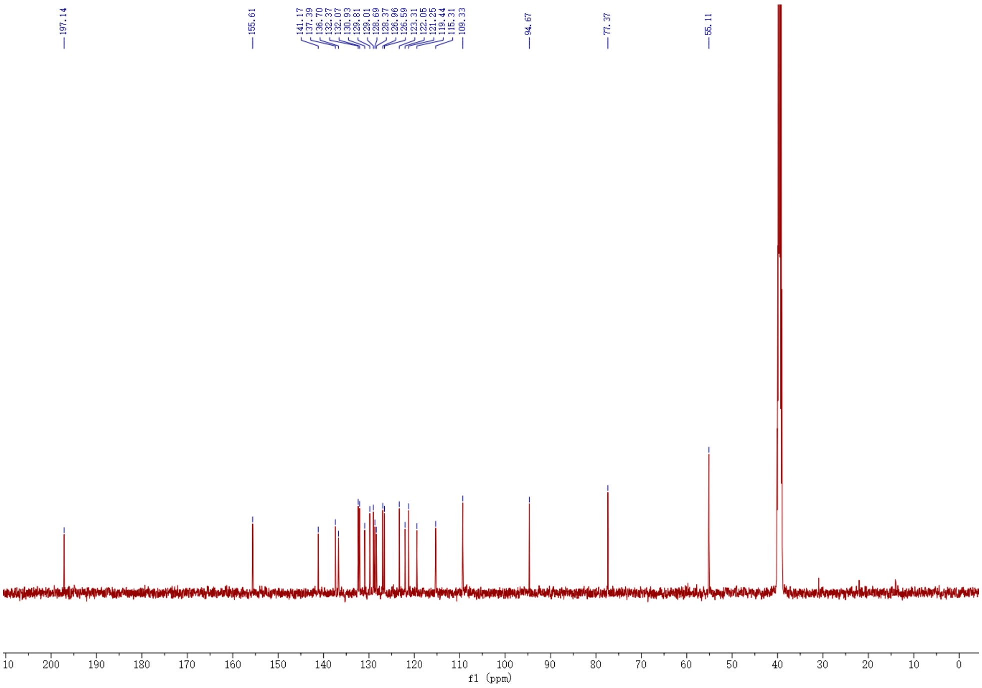
197.14
155.61
141.17
137.39
136.70
132.37
132.07
130.93
129.81
129.01
128.69
128.37
126.96
126.59
123.31
122.05
121.25
119.44
115.31
109.33
94.67
77.37
55.11
10 200 190 180 170 160 150 140 130 120 110 100 90 80 70 60 50 40 30 20 10 0
f1 (ppm)

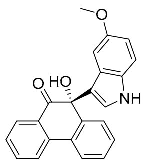

HO
O
NH

(*S*)-4g

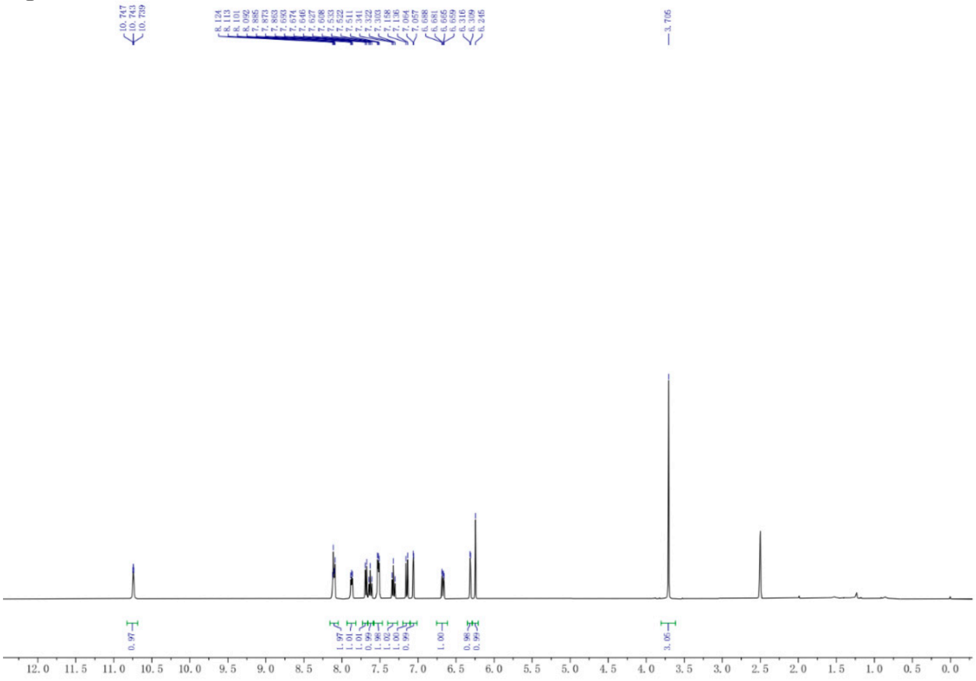

10.747
10.743
10.739
8.124
8.113
8.101
8.092
7.885
7.873
7.863
7.693
7.674
7.646
7.627
7.608
7.533
7.522
7.511
7.341
7.322
7.303
7.158
7.136
7.064
7.057
6.688
6.681
6.665
6.659
6.316
6.309
6.245
3.705
0.97
1.97
1.01
1.01
0.99
1.98
1.02
1.00
0.99
1.00
0.98
0.99
3.05
12.0 11.5 11.0 10.5 10.0 9.5 9.0 8.5 8.0 7.5 7.0 6.5 6.0 5.5 5.0 4.5 4.0 3.5 3.0 2.5 2.0 1.5 1.0 0.5 0.0

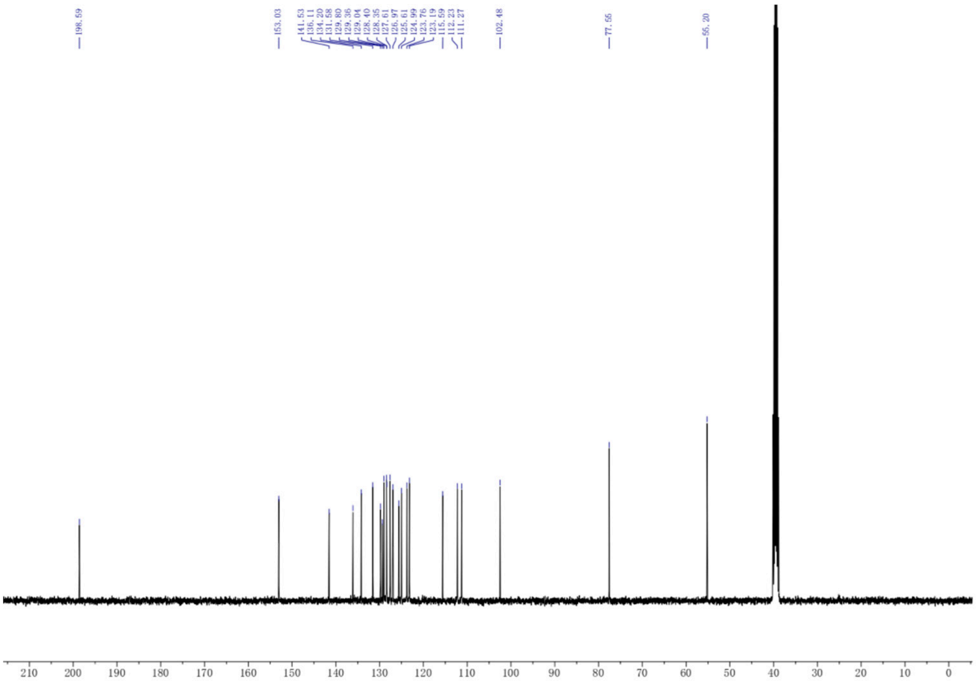

198.59
153.03
141.53
136.11
134.20
131.58
129.80
129.36
129.04
128.40
128.35
127.61
126.97
125.61
124.99
123.76
123.19
115.59
112.23
111.27
102.48
77.55
55.20
210 200 190 180 170 160 150 140 130 120 110 100 90 80 70 60 50 40 30 20 10 0

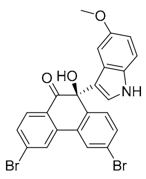

(*S*)-4w

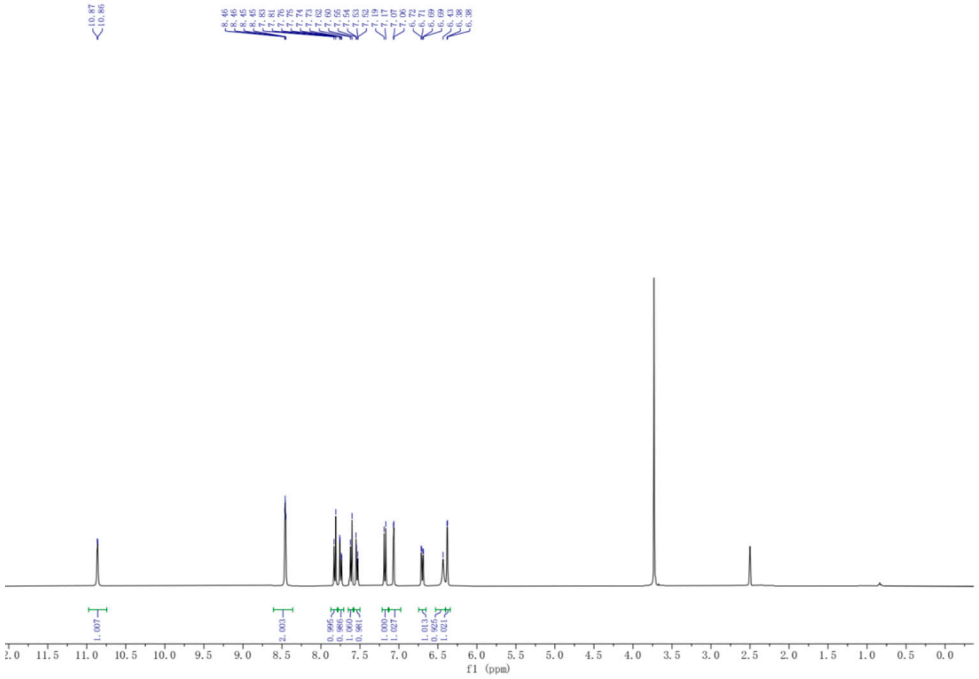

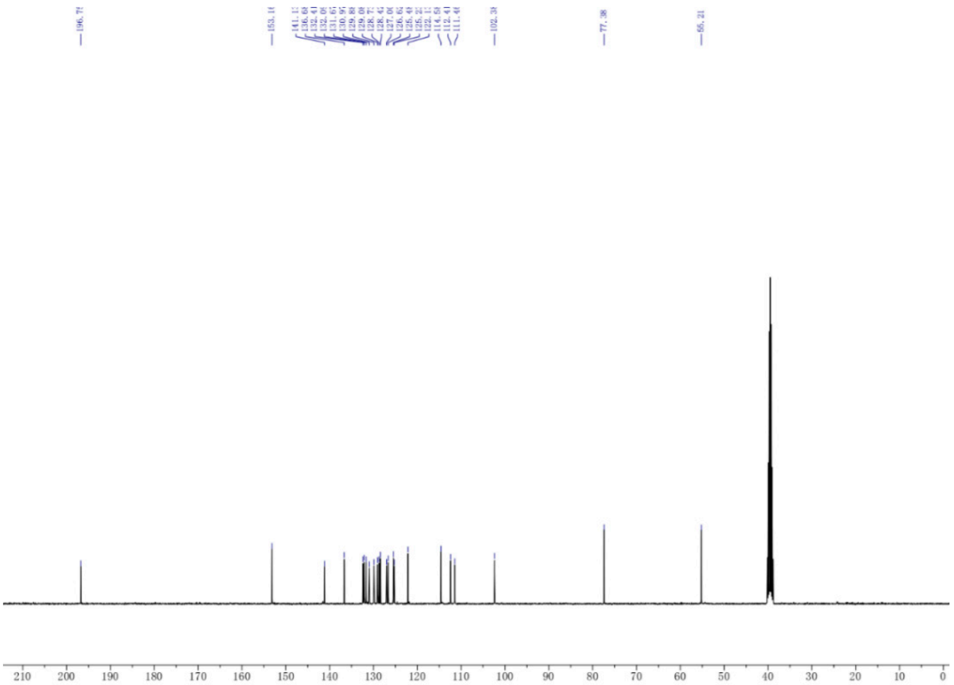

3.HPLC trace

4a

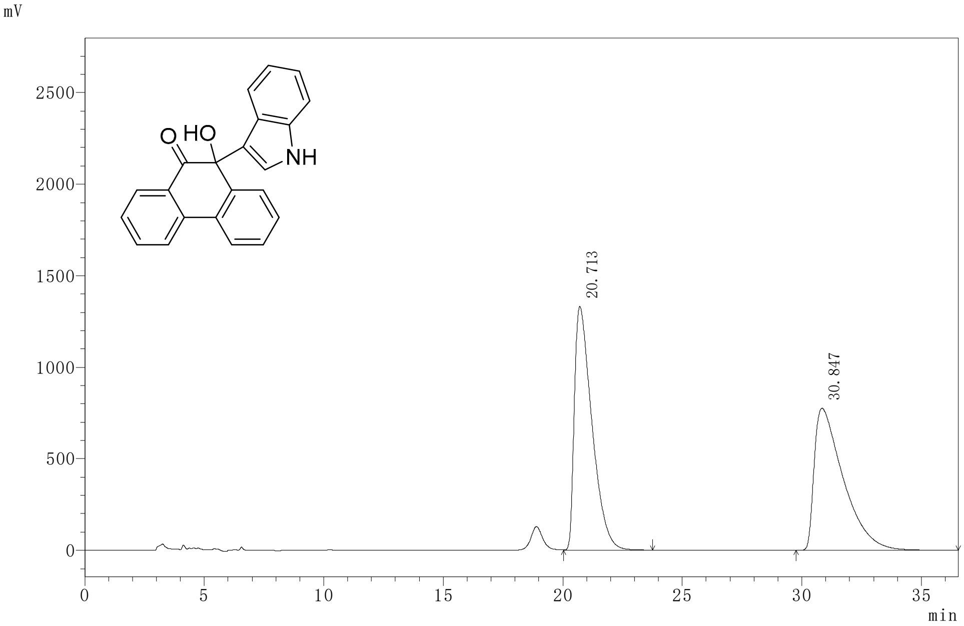

	Retention Time	Area	Height	Area%
1	20.713	68078113	1330352	50.910
2	30.847	65643789	775700	49.090
Total		133721902	2106052	100.000

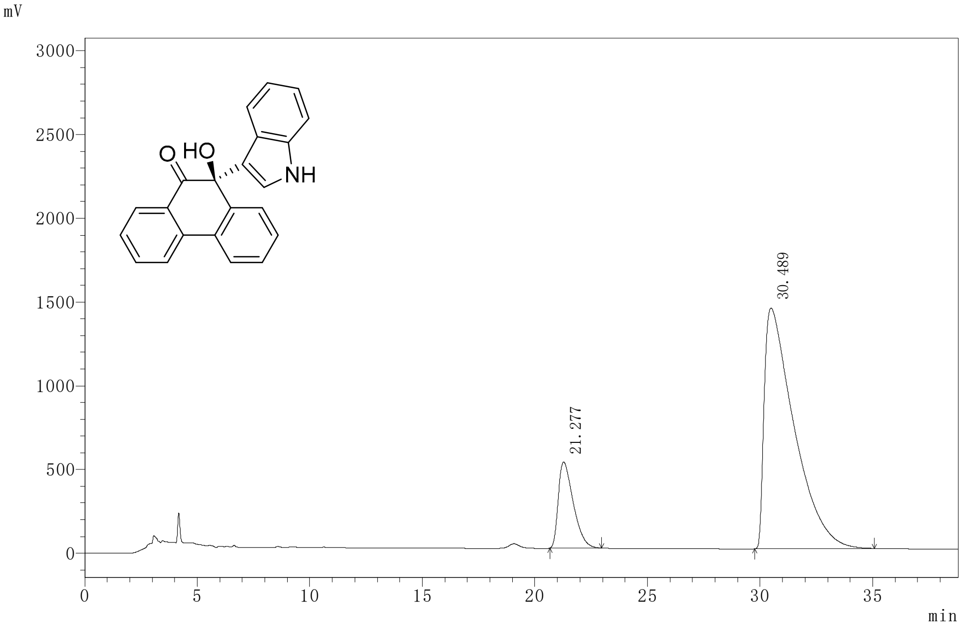

	Retention Time	Area	Height	Area%
1	21.277	23783398	513423	15.239
2	30.489	132286352	1437029	84.761
Total		156069750	1950452	100.000

4d

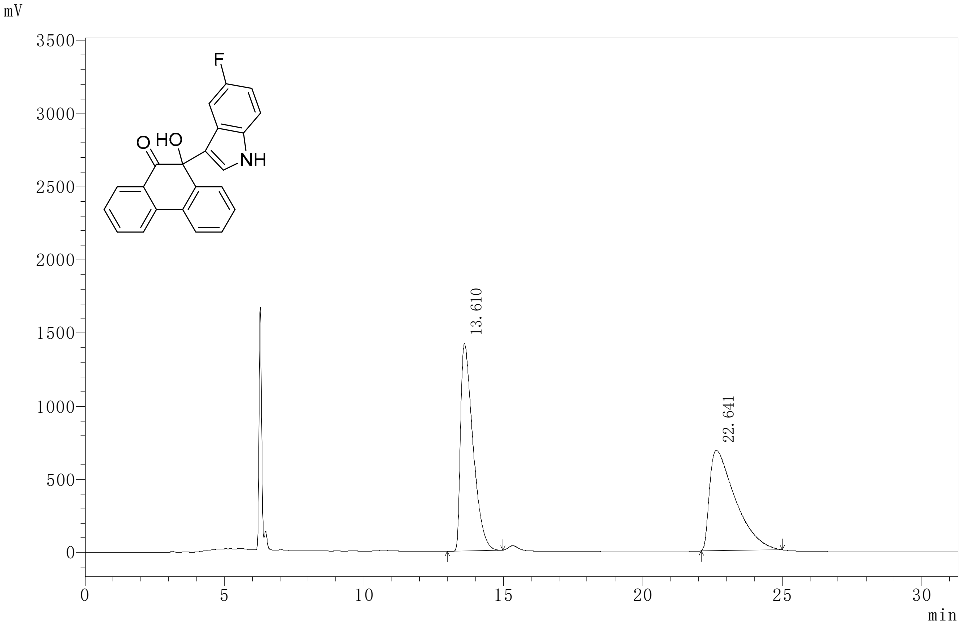

	Retention Time	Area	Height	Area%
1	13.610	43704798	1417303	49.761
2	22.641	44123854	685768	50.239
Total		87828653	2103071	100.000

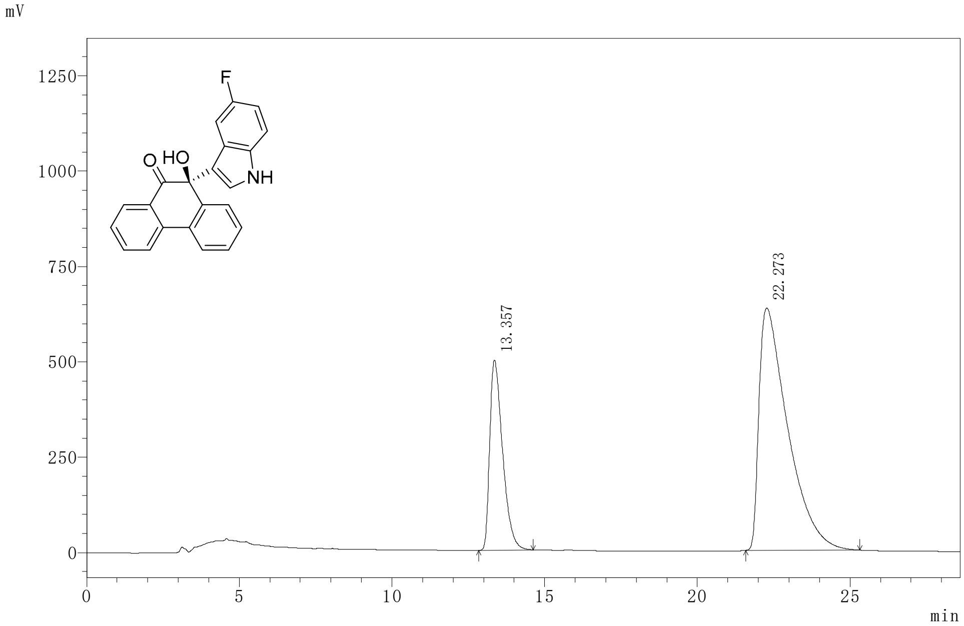

	Retention Time	Area	Height	Area%
1	13.357	14595645	498639	25.979
2	22.273	41587446	635365	74.021
Total		56183091	1134005	100.000

4e

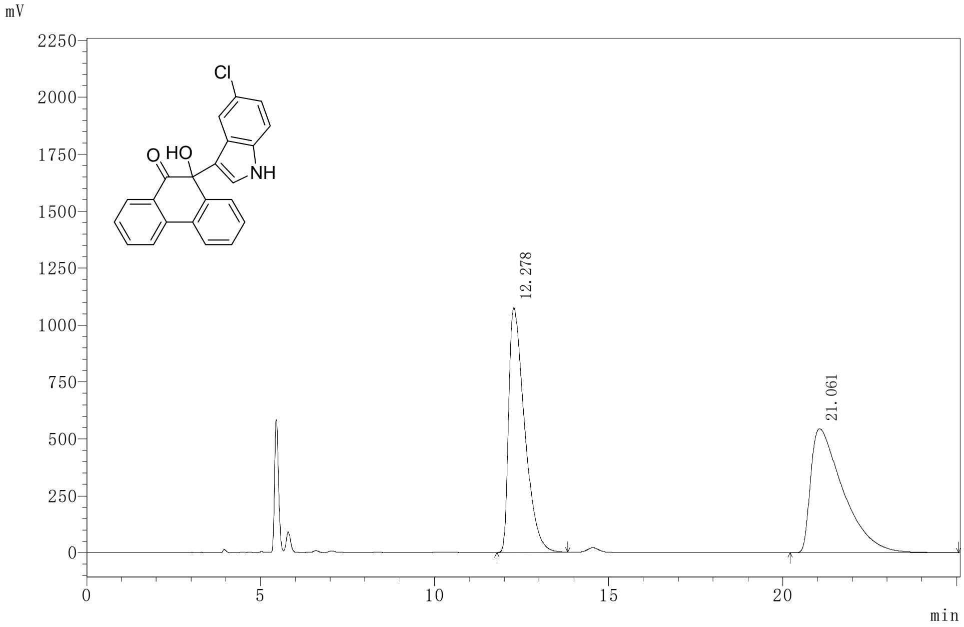

	Retention Time	Area	Height	Area%
1	12.278	34164543	1073707	49.131
2	21.061	35373470	544437	50.869
Total		69538013	1618144	100.000

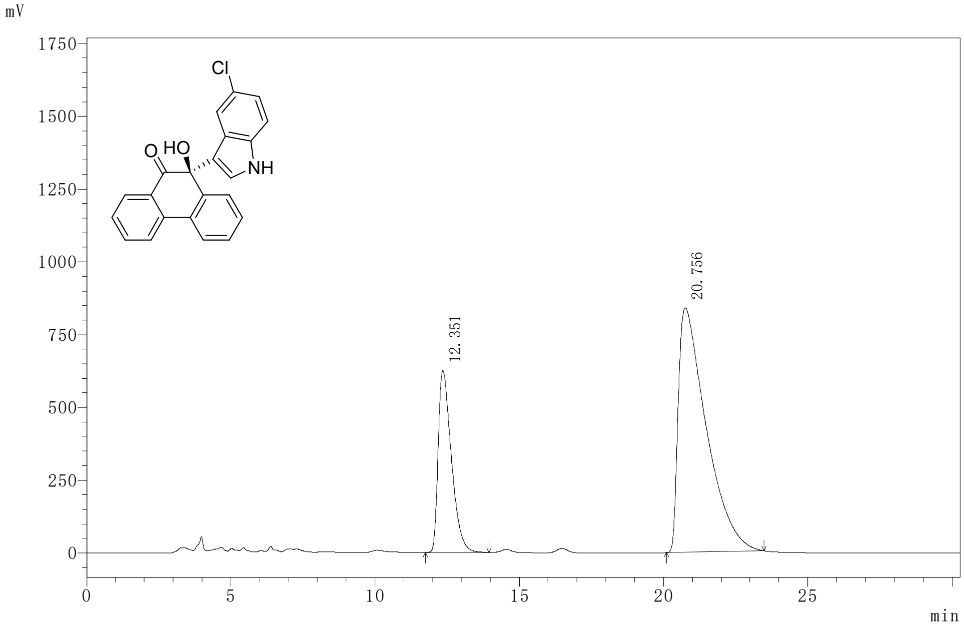

	Retention Time	Area	Height	Area%
1	12.351	19854984	625040	25.958
2	20.756	56633282	839548	74.042
Total		76488265	1464589	100.000

4f

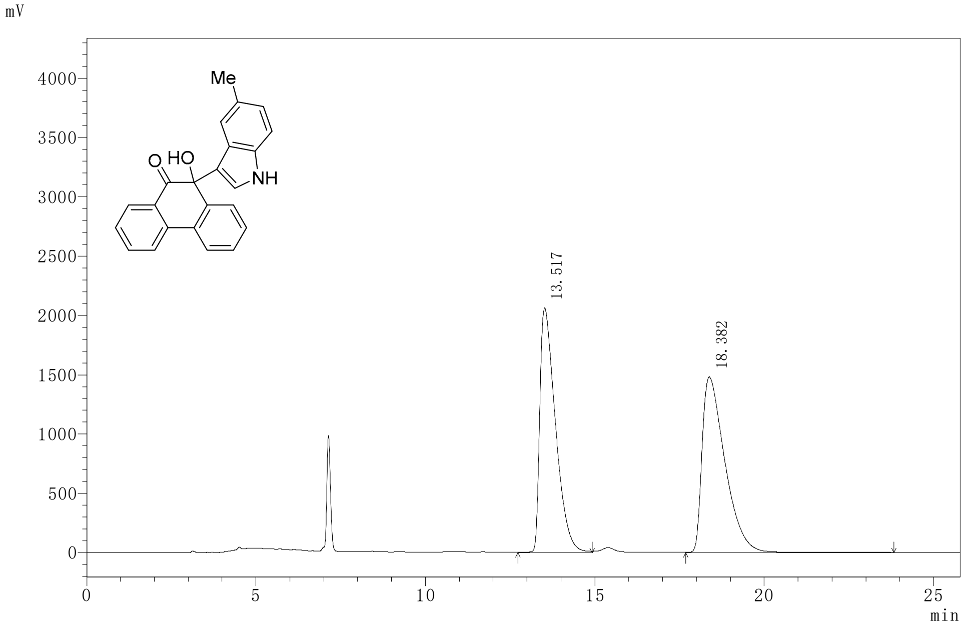

	Retention Time	Area	Height	Area%
1	13.517	66736188	2063217	49.113
2	18.382	69145869	1480559	50.887
Total		135882057	3543776	100.000

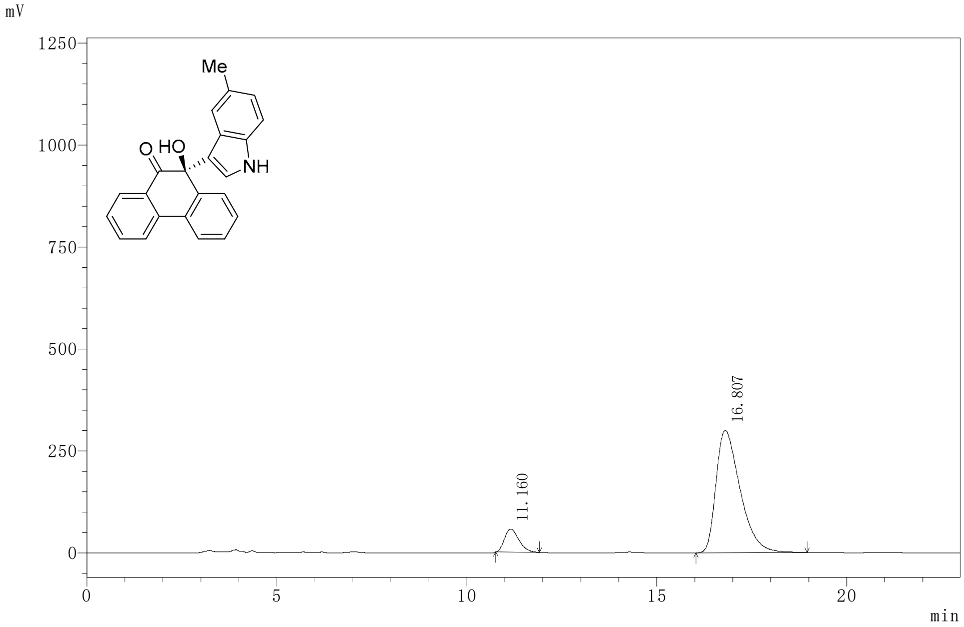

	Retention Time	Area	Height	Area%
1	11.160	1494065	56712	10.127
2	16.807	13259714	299669	89.873
Total		14753779	356381	100.000

4g

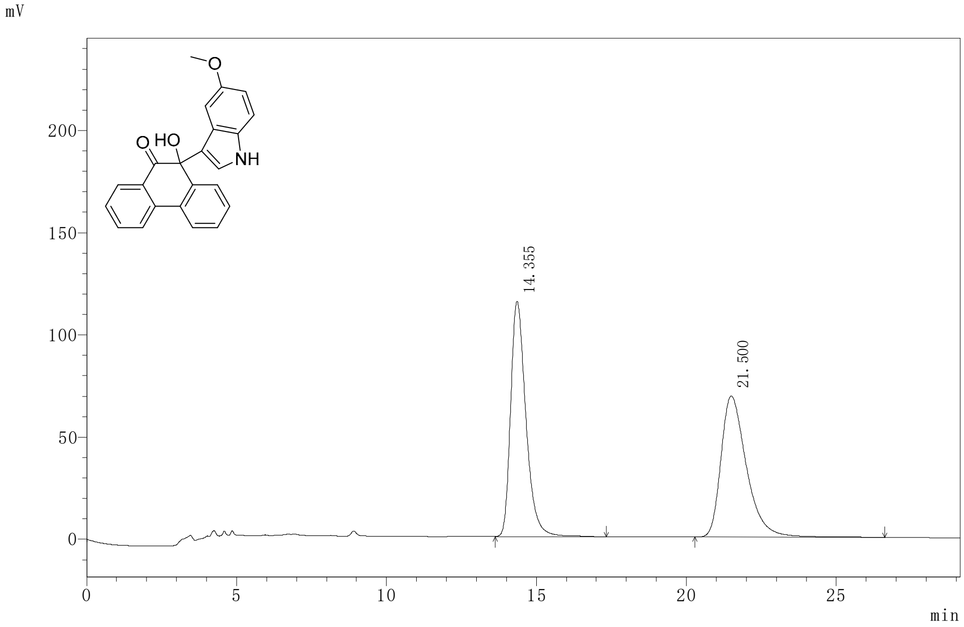

	Retention Time	Area	Height	Area%
1	14.355	4089445	115192	49.956
2	21.500	4096617	69051	50.044
Total		8186062	184243	100.000

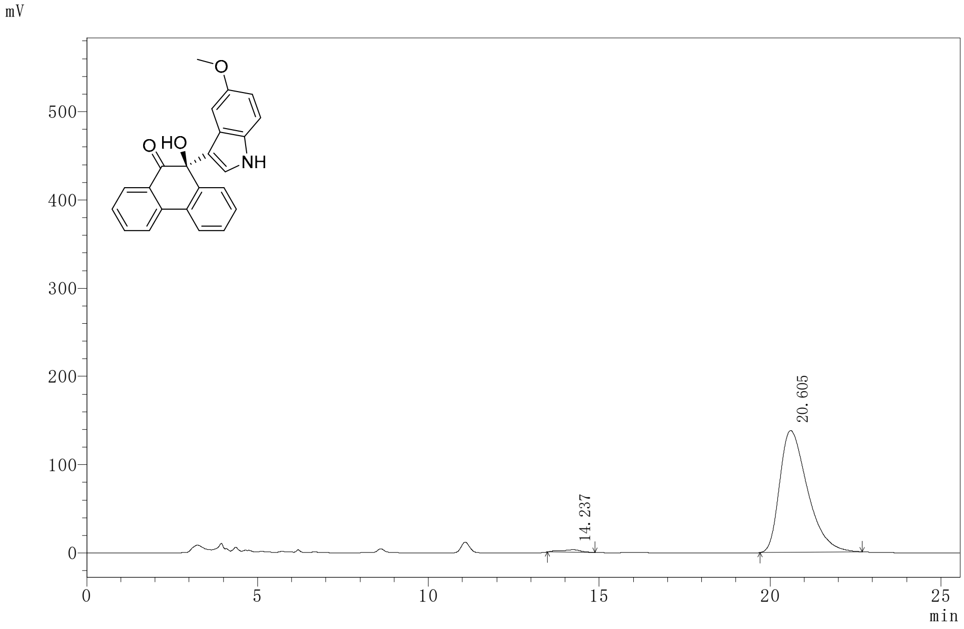

	Retention Time	Area	Height	Area%
1	14.237	120839	2850	1.518
2	20.605	7838358	138012	98.482
Total		7959197	140862	100.000

4i

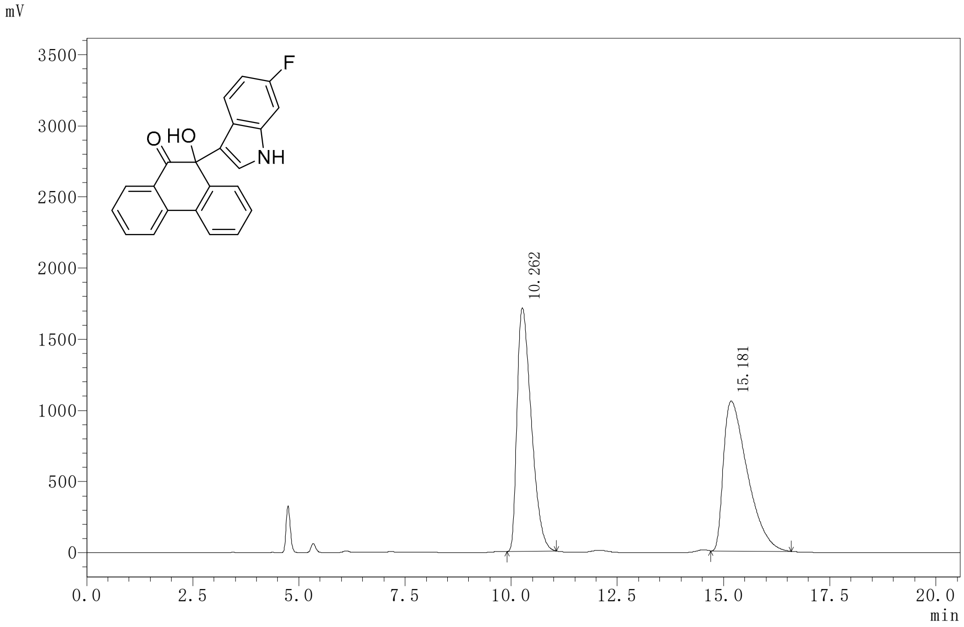

	Retention Time	Area	Height	Area%
1	10.262	40174186	1710809	49.247
2	15.181	41403459	1053178	50.753
Total		81577645	2763986	100.000

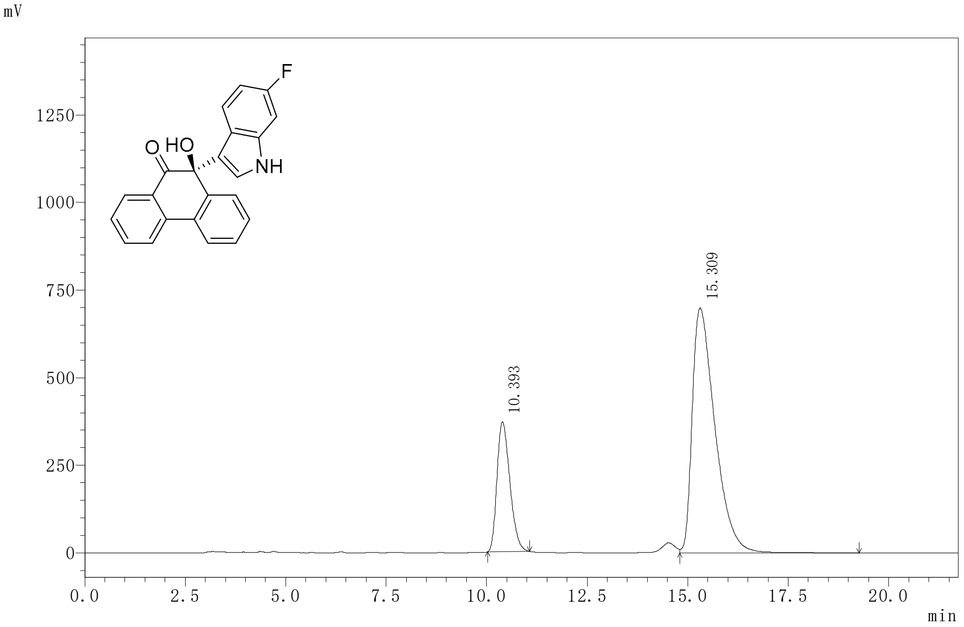

	Retention Time	Area	Height	Area%
1	10.393	8258008	370774	23.069
2	15.309	27539332	699295	76.931
Total		35797340	1070069	100.000

4j

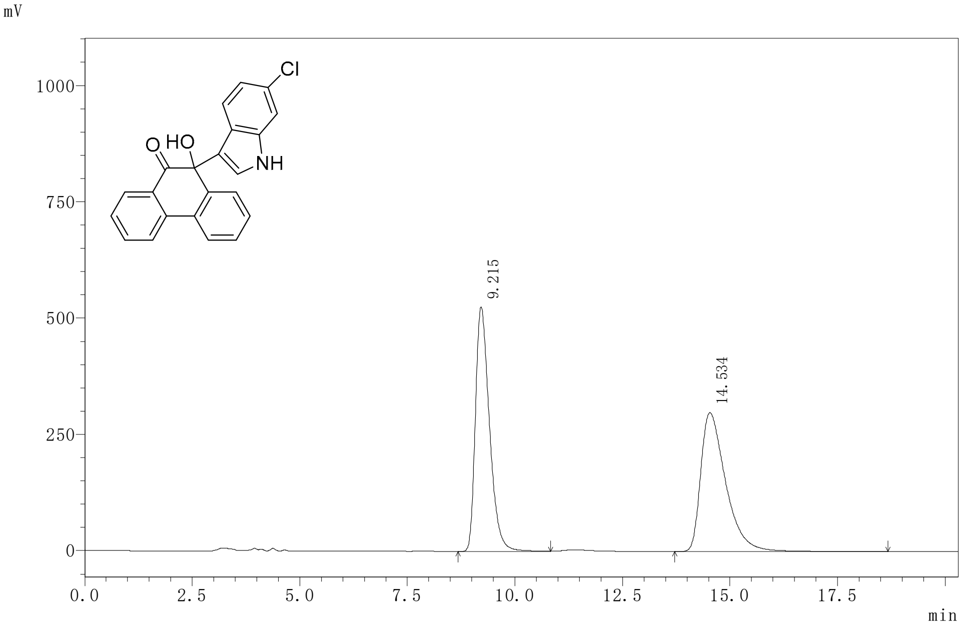

	Retention Time	Area	Height	Area%
1	9.215	11976506	525883	49.769
2	14.534	12087451	298842	50.231
Total		24063957	824725	100.000

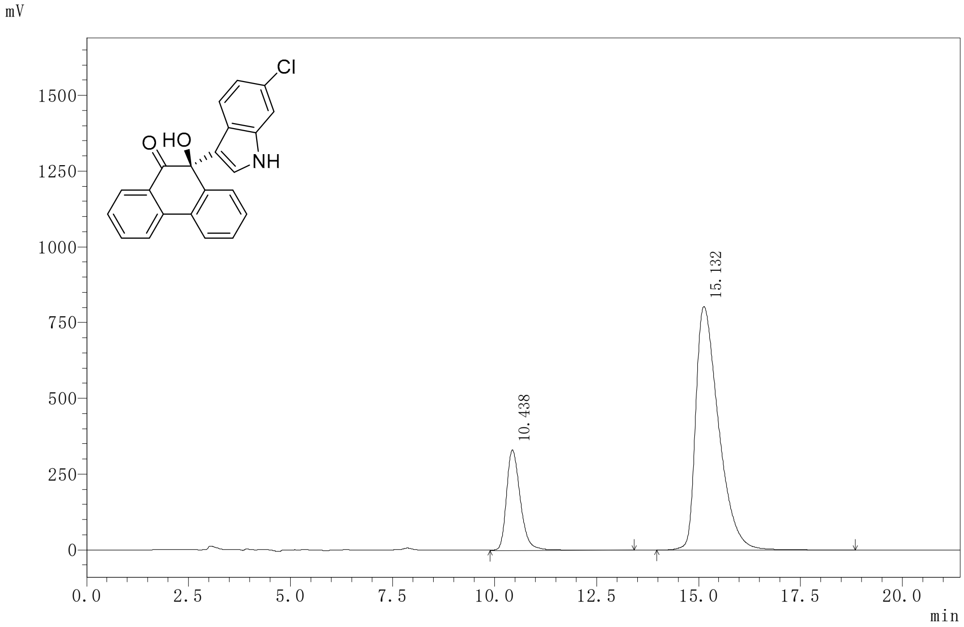

	Retention Time	Area	Height	Area%
1	10.438	7927290	331650	20.134
2	15.132	31444741	803911	79.866
Total		39372031	1135560	100.000

4k

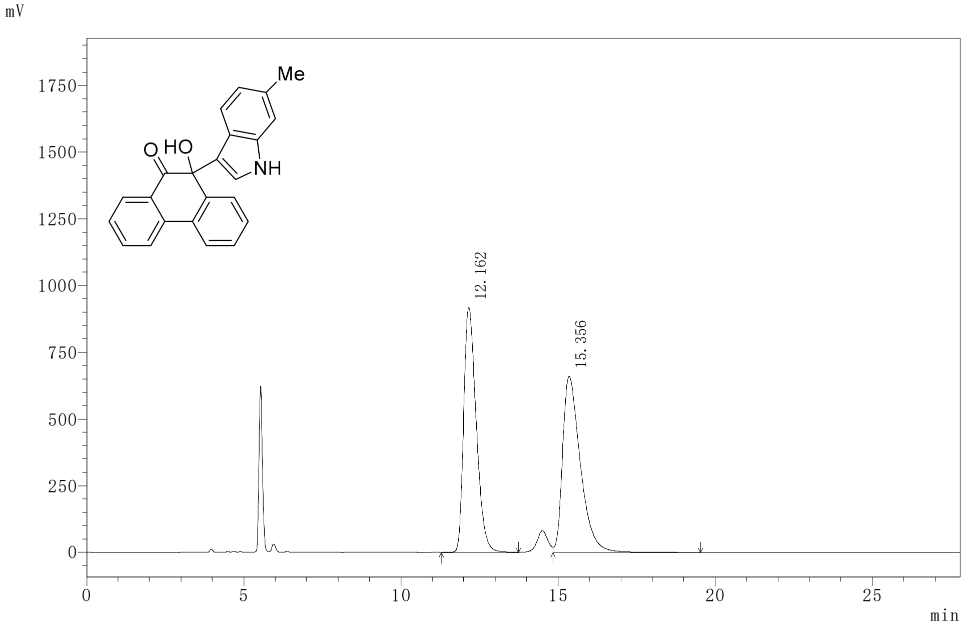

	Retention Time	Area	Height	Area%
1	12.162	25484500	917004	49.500
2	15.356	25999412	660101	50.500
Total		51483913	1577105	100.000

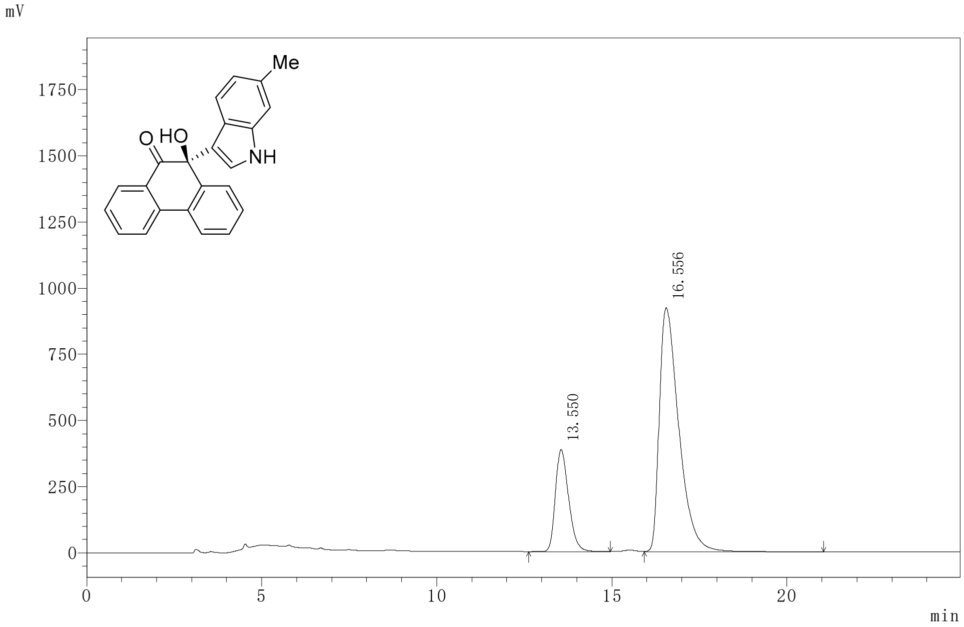

	Retention Time	Area	Height	Area%
1	13.550	10178013	385521	22.150
2	16.556	35772882	921668	77.850
Total		45950895	1307189	100.000

4l

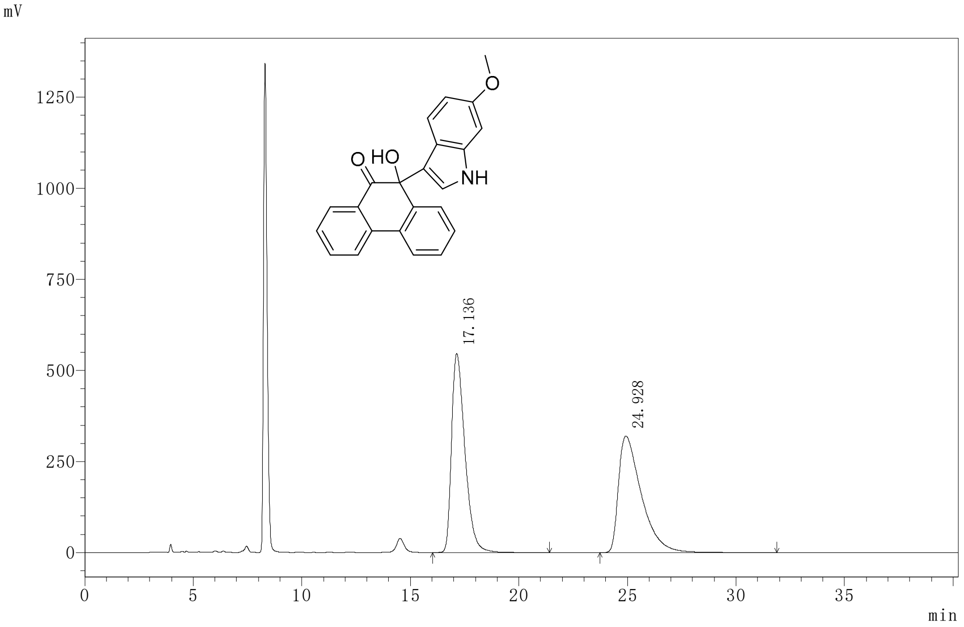

	Retention Time	Area	Height	Area%
1	17.136	23464291	546458	49.866
2	24.928	23590743	320518	50.134
Total		47055034	866976	100.000

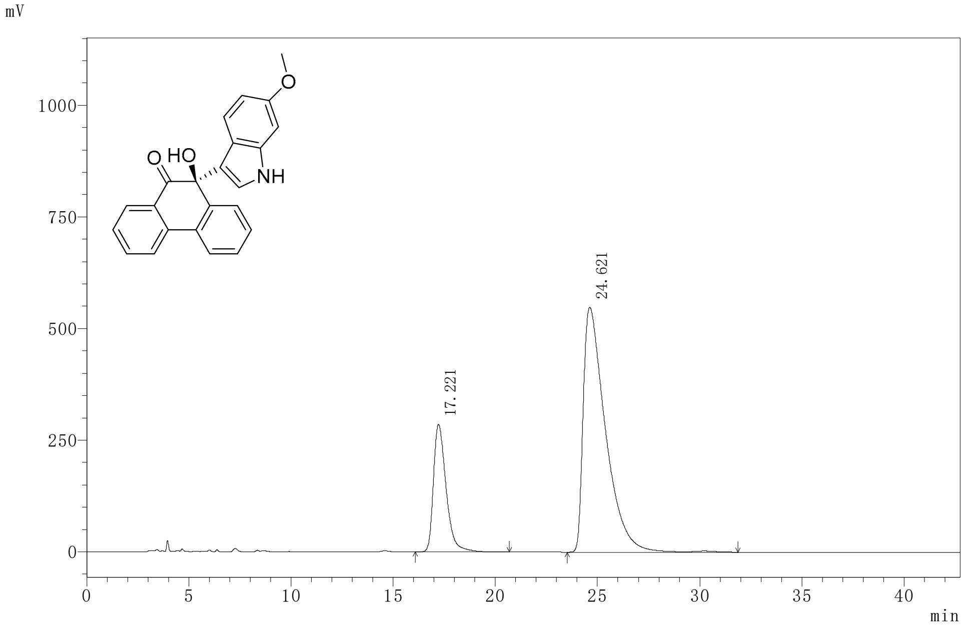

	Retention Time	Area	Height	Area%
1	17.221	12368106	286097	22.899
2	24.621	41642529	548370	77.101
Total		54010635	834467	100.000

4o

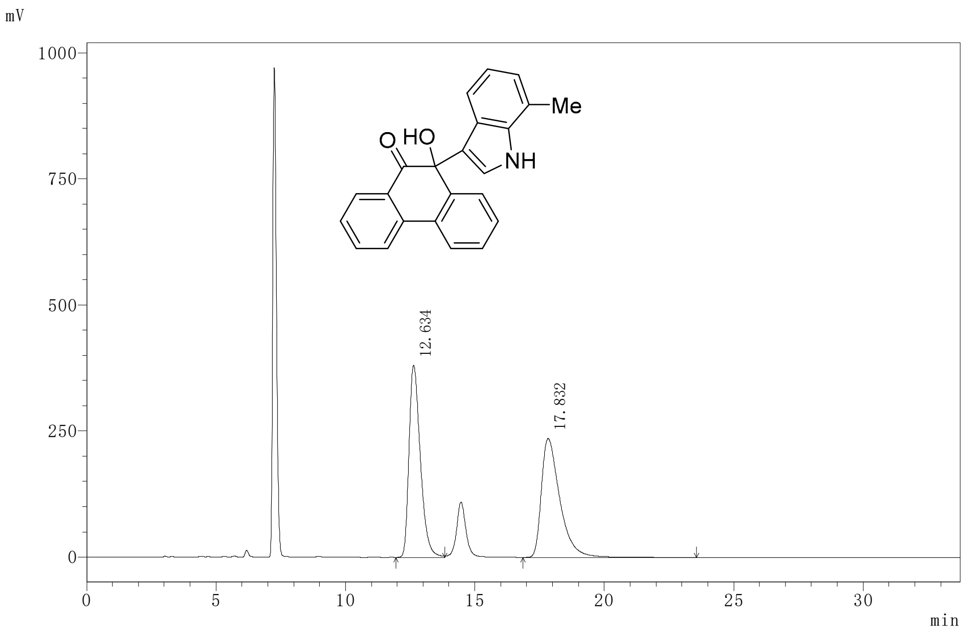

	Retention Time	Area	Height	Area%
1	12.634	11598840	380890	49.714
2	17.832	11732423	236104	50.286
Total		23331263	616994	100.000

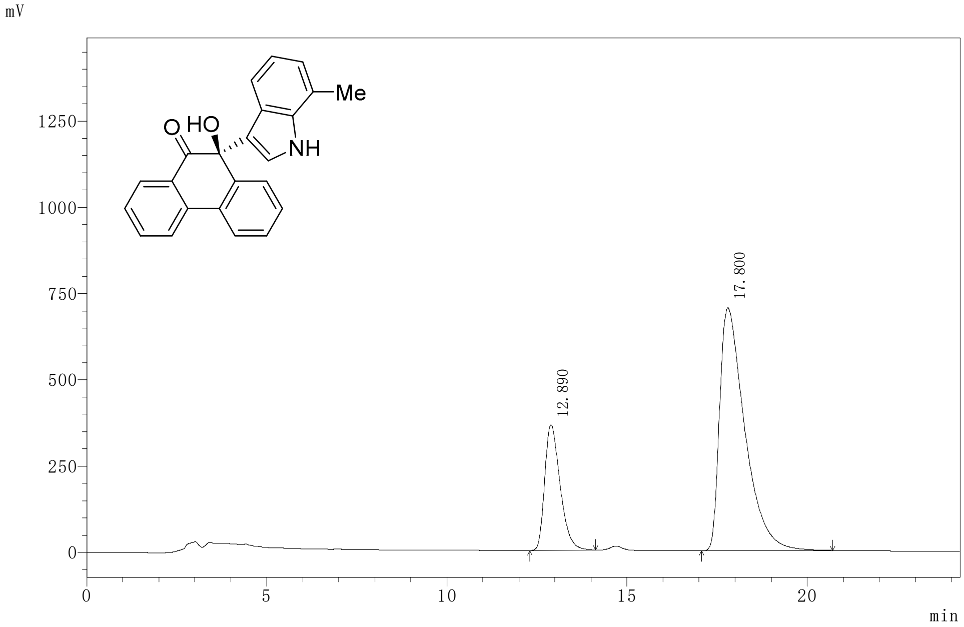

	Retention Time	Area	Height	Area%
1	12.890	10834596	363908	23.992
2	17.800	34324728	704670	76.008
Total		45159324	1068578	100.000

4p

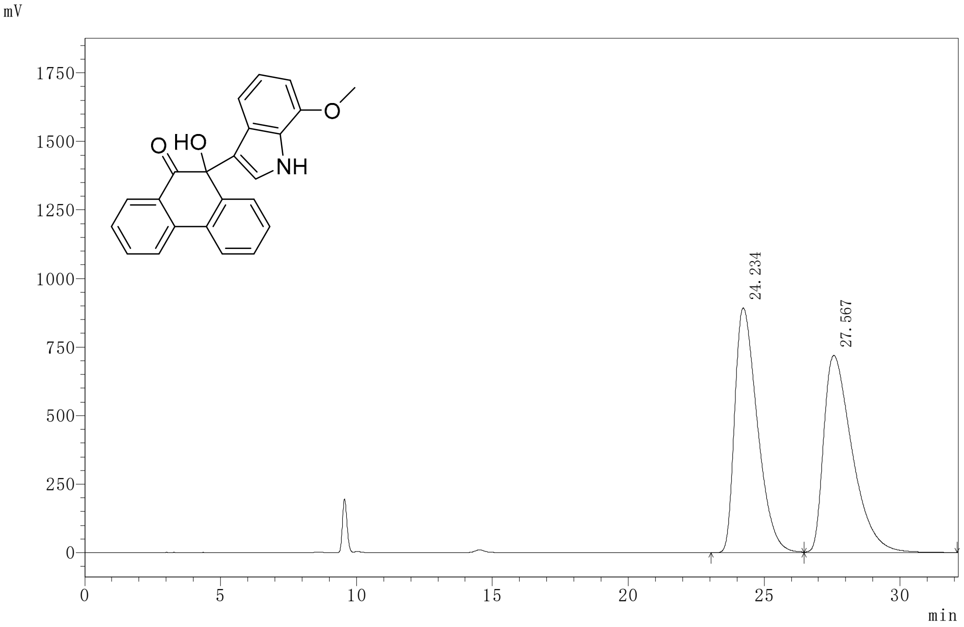

	Retention Time	Area	Height	Area%
1	24.234	51985427	892981	49.664
2	27.567	52689619	719624	50.336
Total		104675046	1612605	100.000

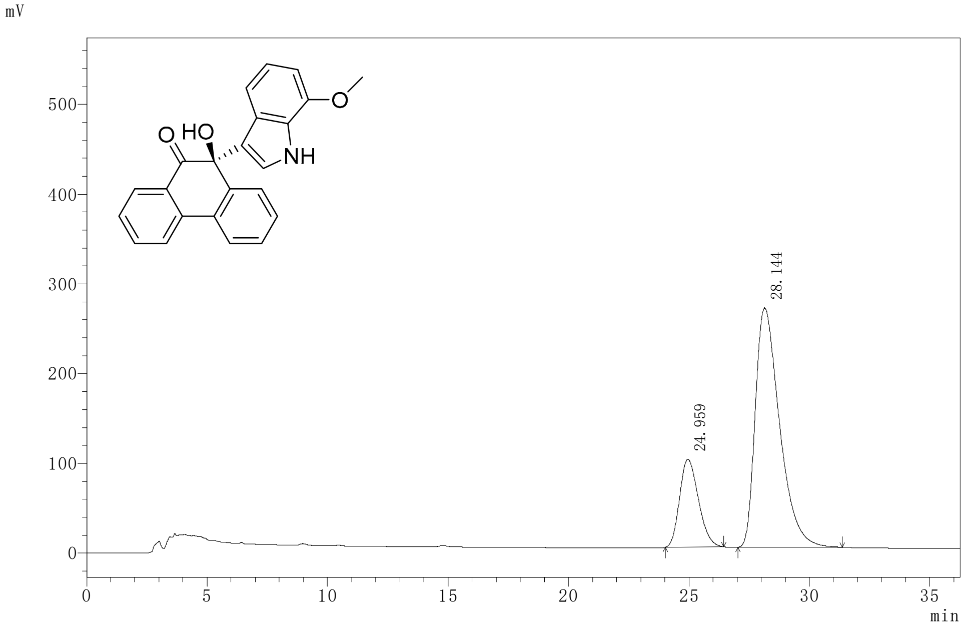

	Retention Time	Area	Height	Area%
1	24.959	5325010	97840	22.128
2	28.144	18740037	266799	77.872
Total		24065047	364639	100.000

4q

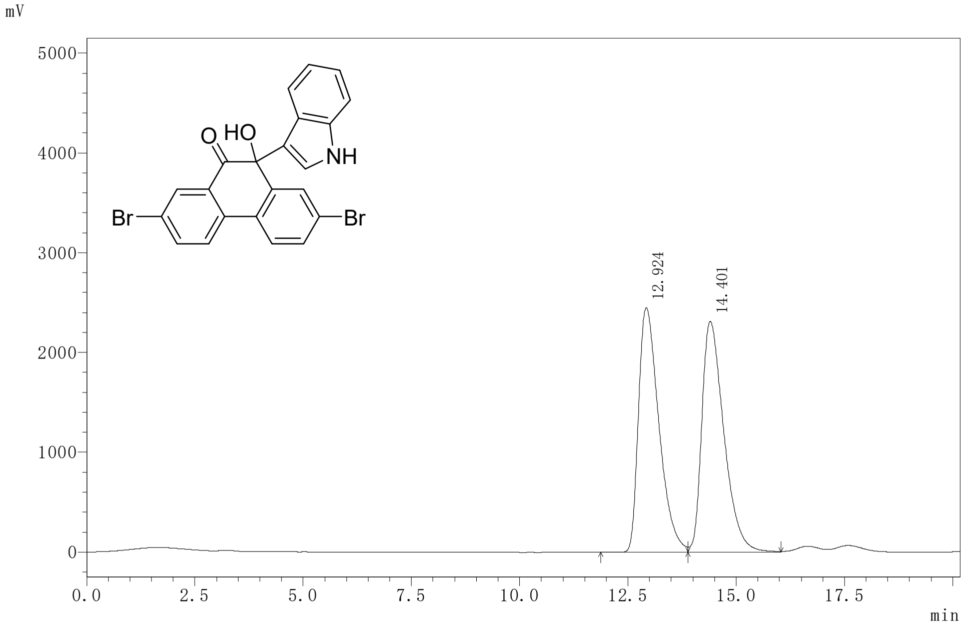

	Retention Time	Area	Height	Area%
1	12.924	80623135	2454243	49.500
2	14.401	82252503	2315735	50.500
Total		162875638	4769977	100.000

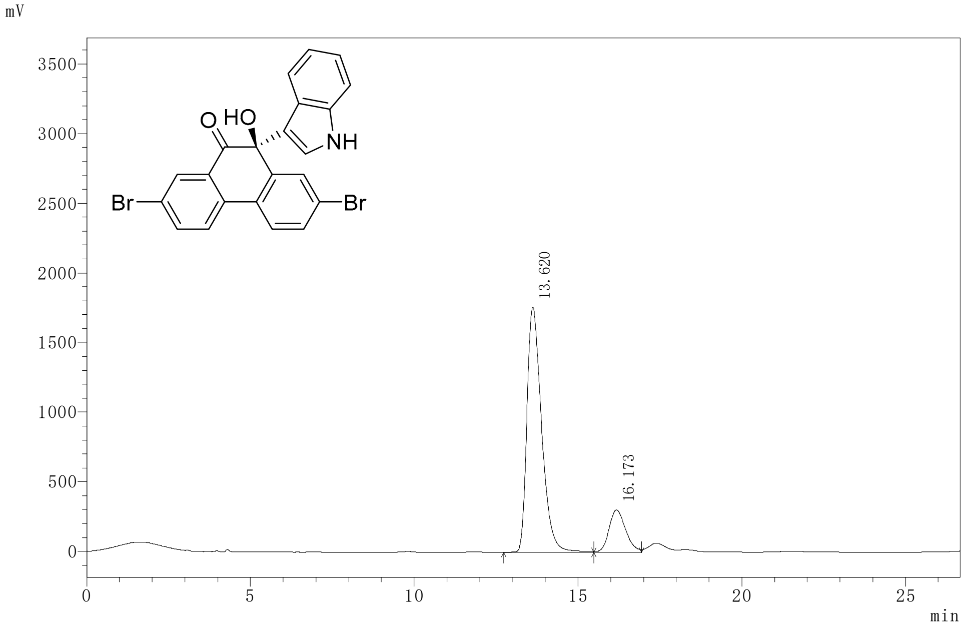

	Retention Time	Area	Height	Area%
1	13.620	54638873	1760656	83.624
2	16.173	10699495	303147	16.376
Total		65338368	2063803	100.000

4r

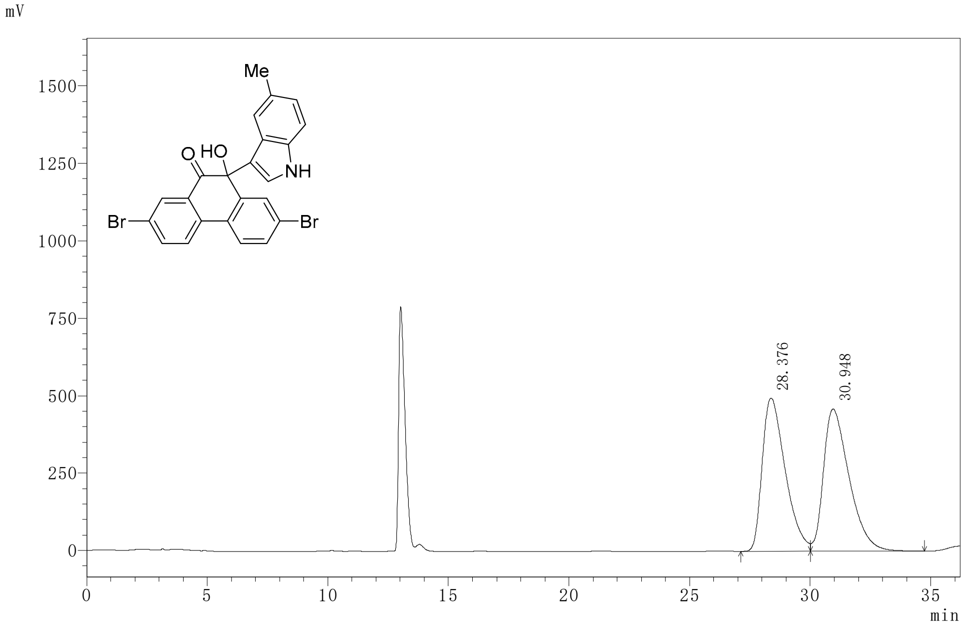

	Retention Time	Area	Height	Area%
1	28.376	32847518	495160	49.129
2	30.948	34011647	459002	50.871
Total		66859164	954161	100.000

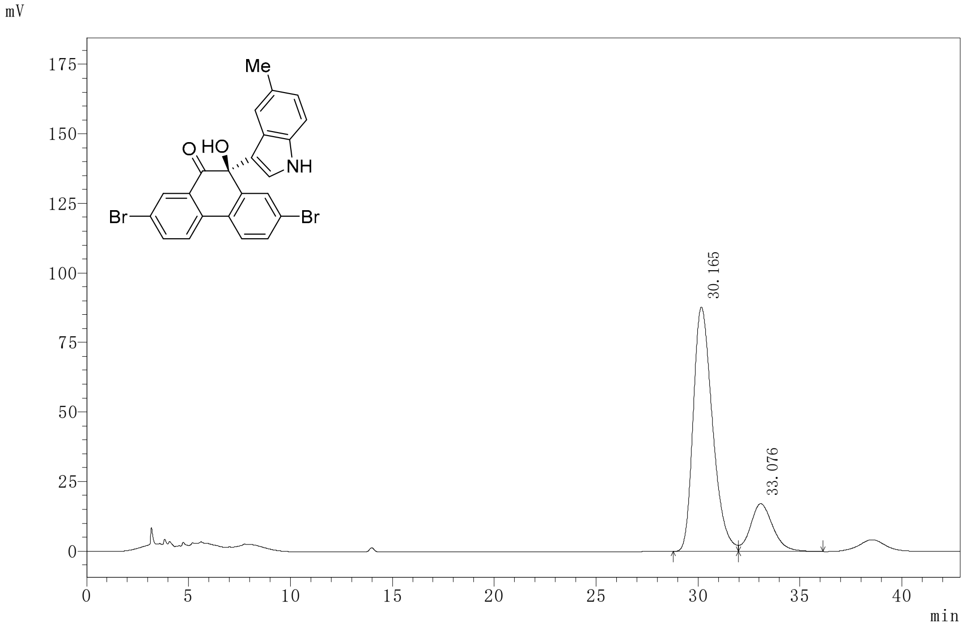

	Retention Time	Area	Height	Area%
1	30.165	5784212	87909	81.495
2	33.076	1313426	17184	18.505
Total		7097638	105093	100.000

4s

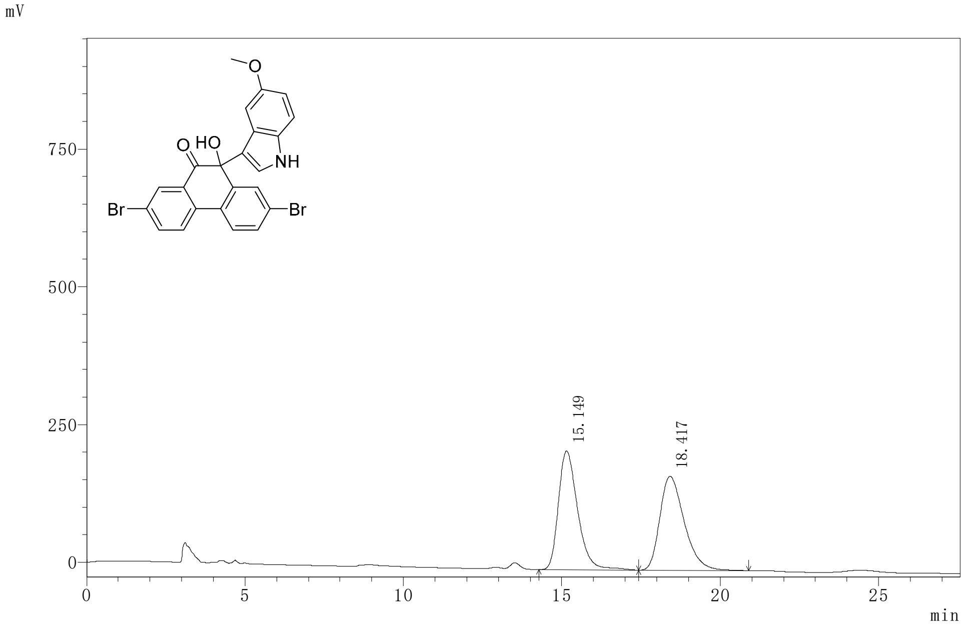

	Retention Time	Area	Height	Area%
1	15.149	9122839	215377	50.013
2	18.417	9118270	170446	49.987
Total		18241109	385823	100.000

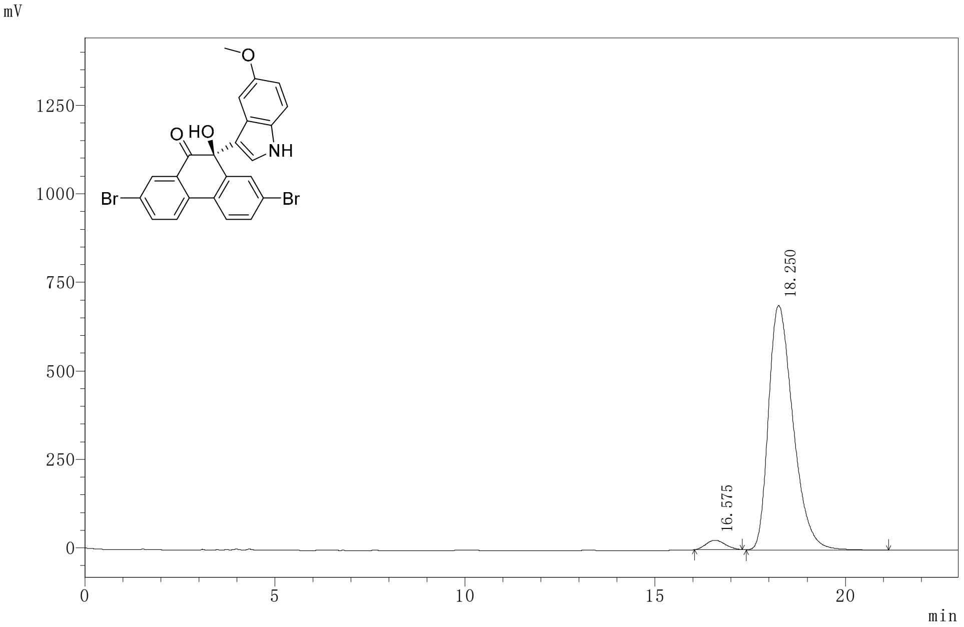

	Retention Time	Area	Height	Area%
1	16.575	936539	26604	2.917
2	18.250	31173389	691506	97.083
Total		32109928	718110	100.000

4t

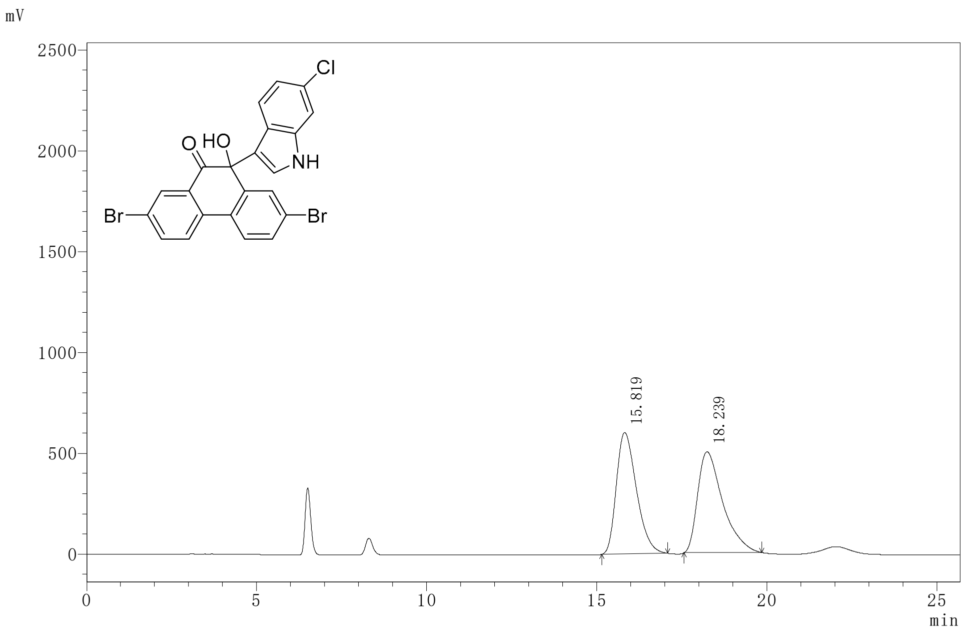

	Retention Time	Area	Height	Area%
1	15.819	24692461	601718	49.073
2	18.239	25624939	500181	50.927
Total		50317400	1101899	100.000

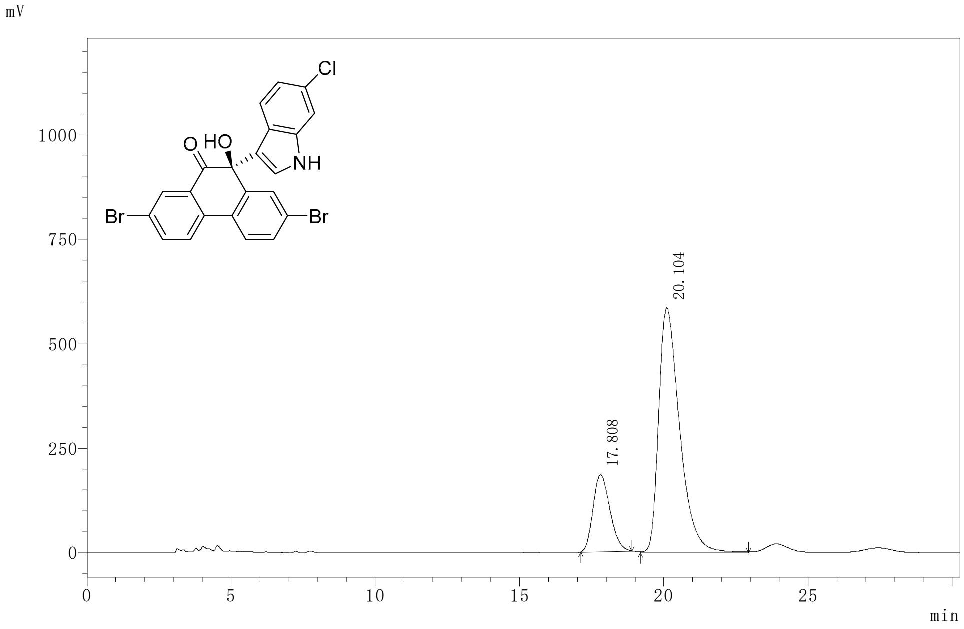

	Retention Time	Area	Height	Area%
1	17.808	7491907	183942	20.107
2	20.104	29769038	585475	79.893
Total		37260945	769418	100.000

4u

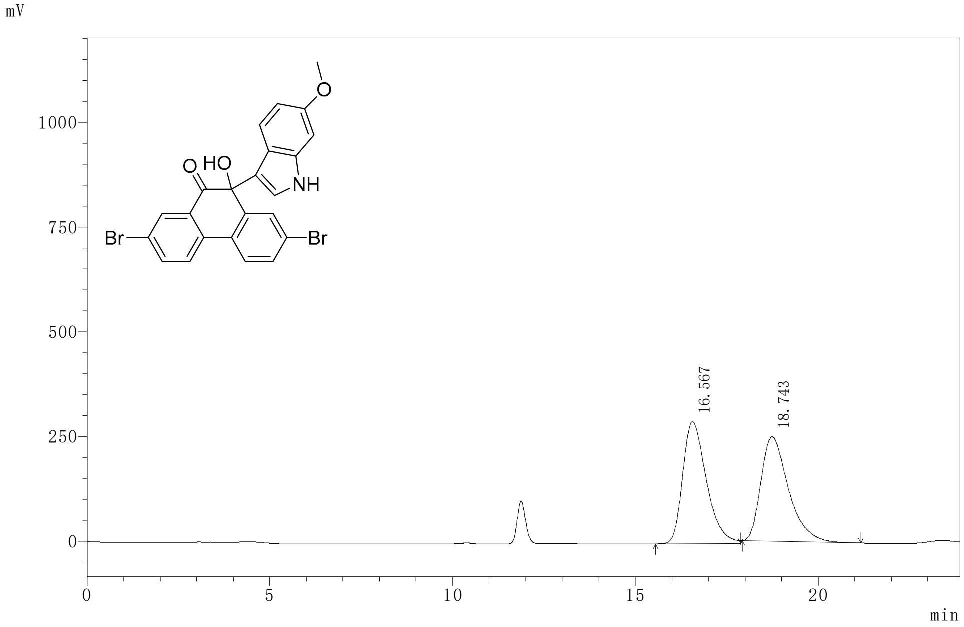

	Retention Time	Area	Height	Area%
1	16.567	13107415	290873	50.044
2	18.743	13084469	249619	49.956
Total		26191885	540492	100.000

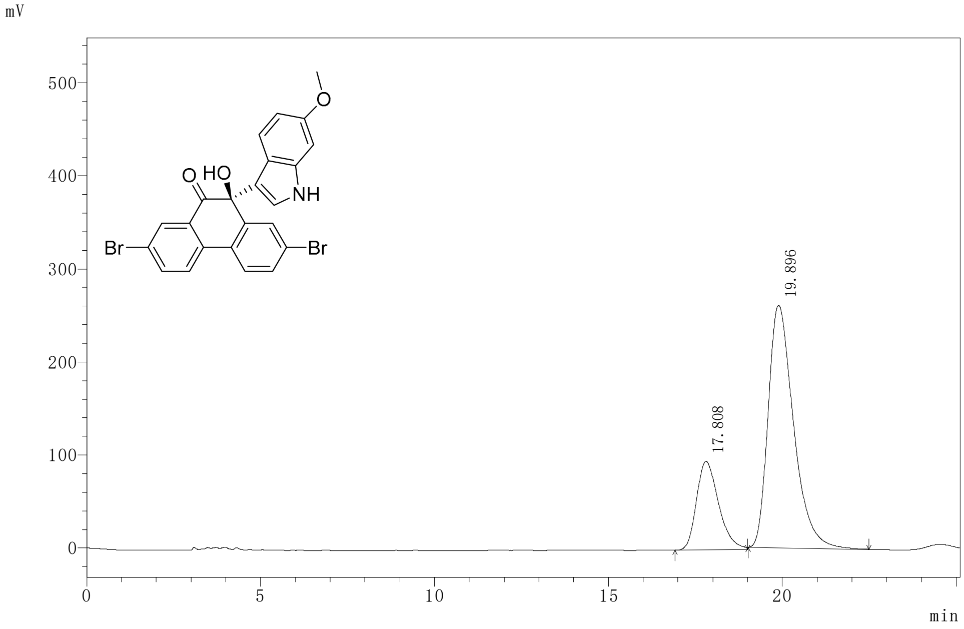

	Retention Time	Area	Height	Area%
1	17.808	4225834	95327	24.193
2	19.896	13241291	260457	75.807
Total		17467124	355784	100.000

4v

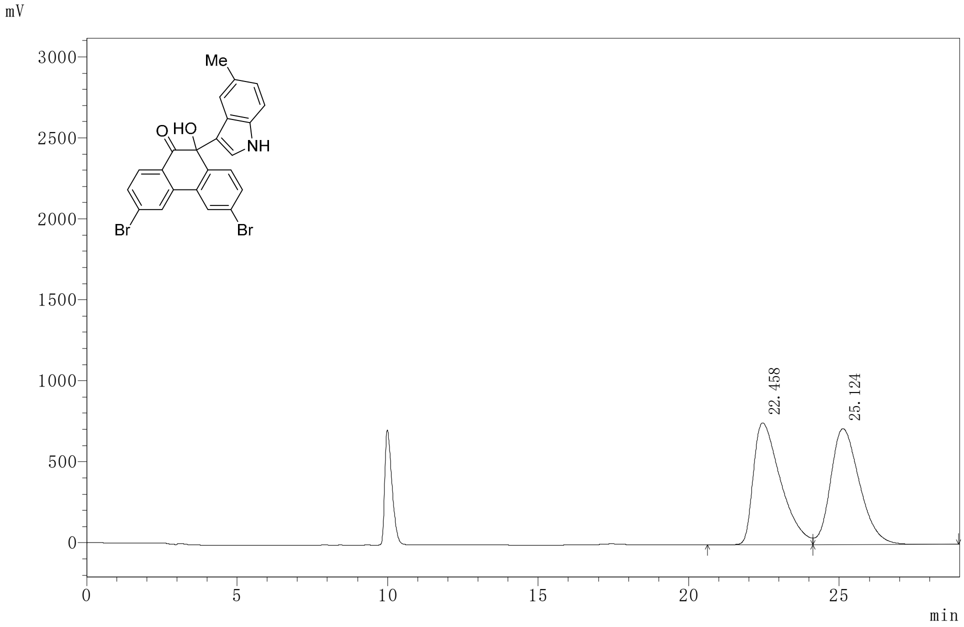

	Retention Time	Area	Height	Area%
1	22.458	47725946	753585	49.826
2	25.124	48059684	717045	50.174
Total		95785630	1470630	100.000

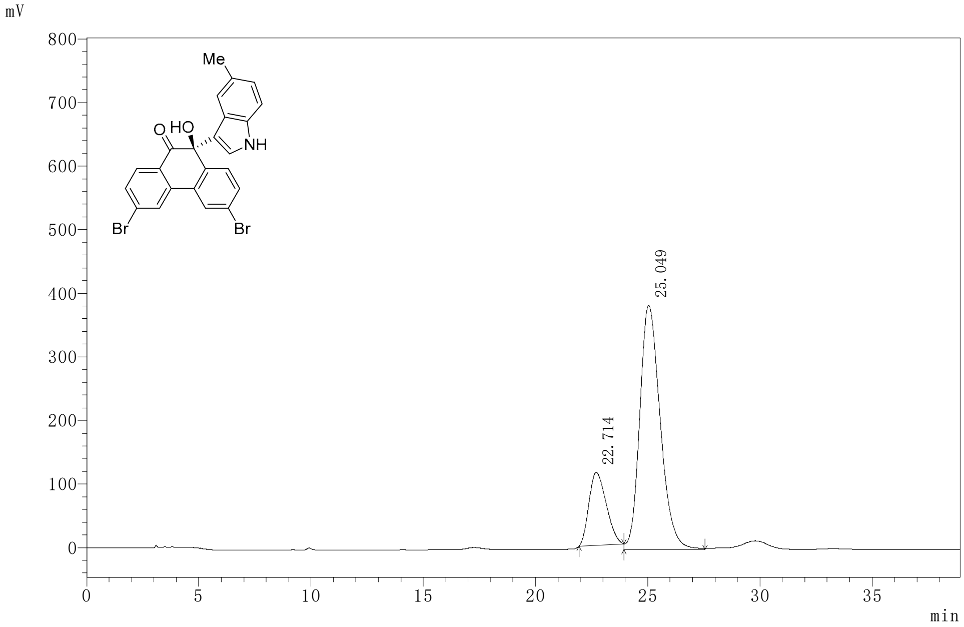

	Retention Time	Area	Height	Area%
1	22.714	6150754	114609	20.118
2	25.049	24422011	384341	79.882
Total		30572765	498950	100.000

4w

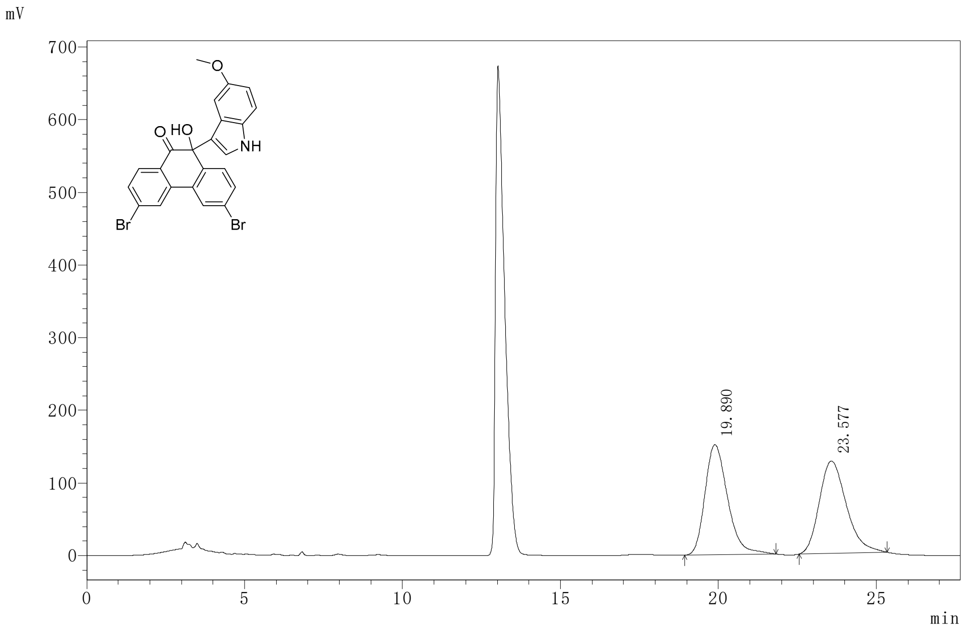

	Retention Time	Area	Height	Area%
1	19.890	7566017	151561	49.984
2	23.577	7570816	127078	50.016
Total		15136832	278640	100.000

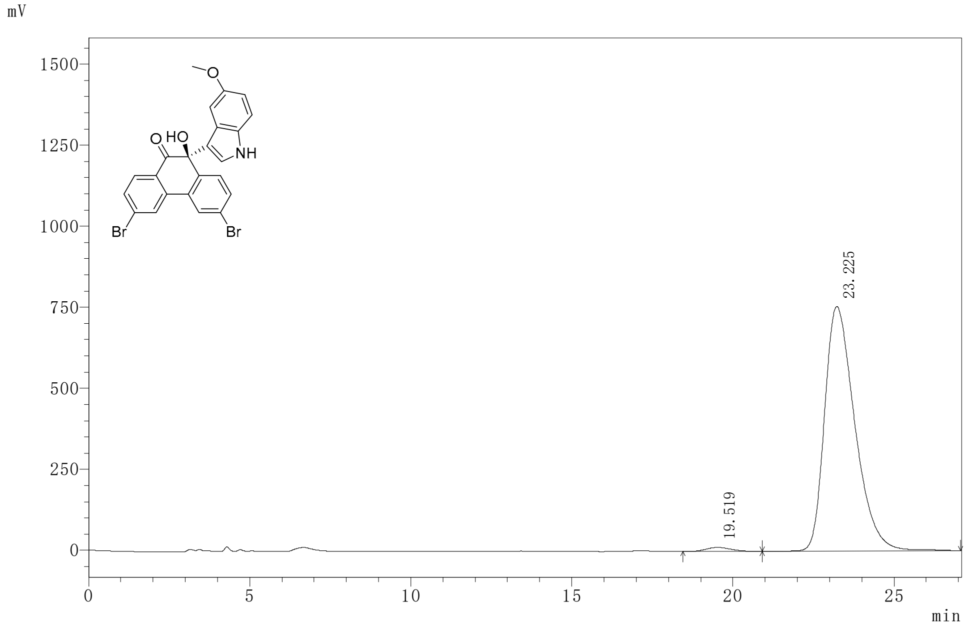

	Retention Time	Area	Height	Area%
1	19.519	662371	12389	1.324
2	23.225	49374684	754508	98.676
Total		50037055	766897	100.000

4x

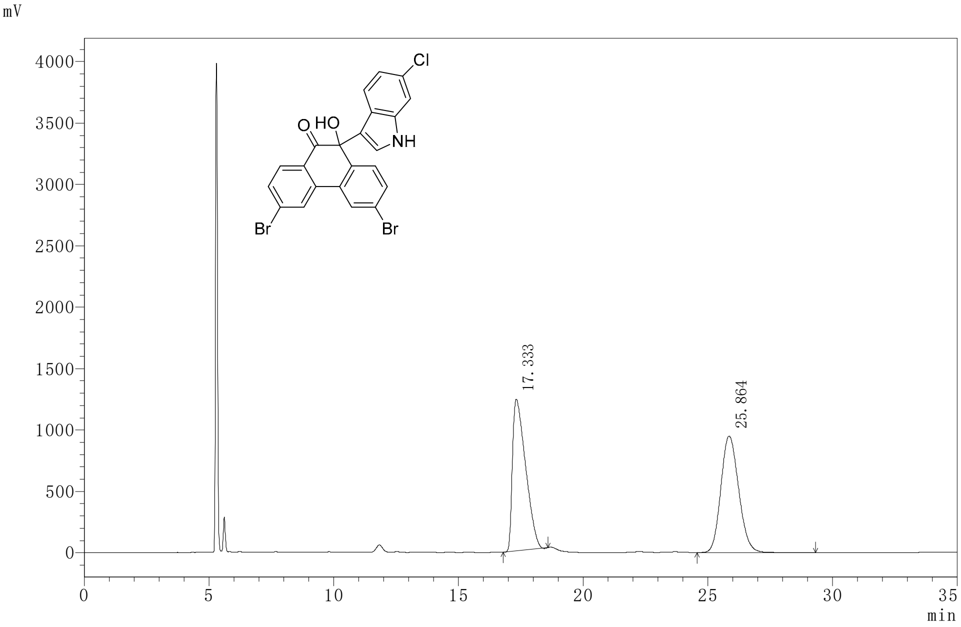

	Retention Time	Area	Height	Area%
1	17.333	45175231	1236687	49.003
2	25.864	47013284	949189	50.997
Total		92188516	2185876	100.000

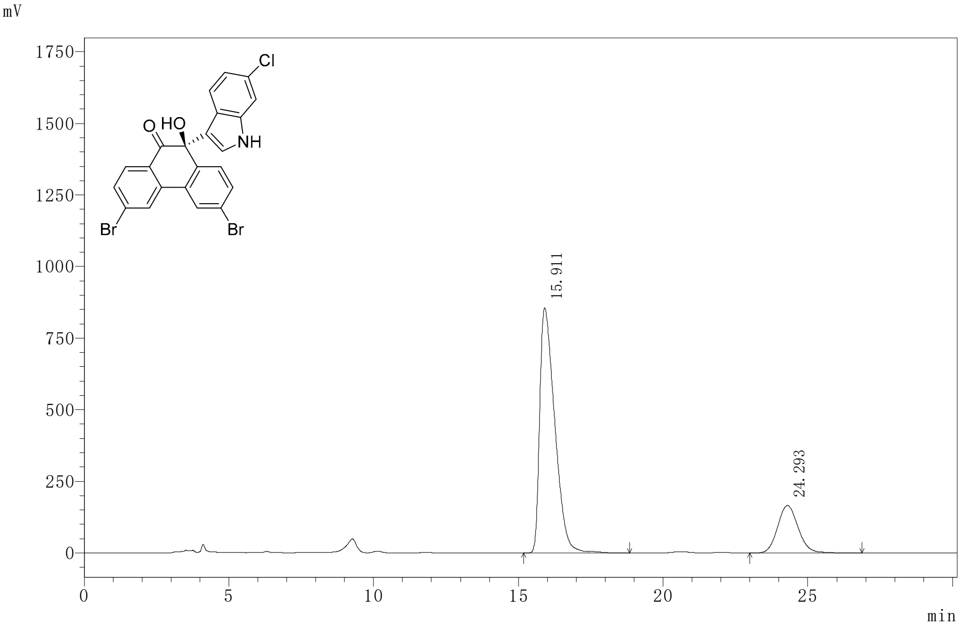

	Retention Time	Area	Height	Area%
1	15.911	30918468	855823	79.054
2	24.293	8191991	165797	20.946
Total		39110459	1021620	100.000

4y

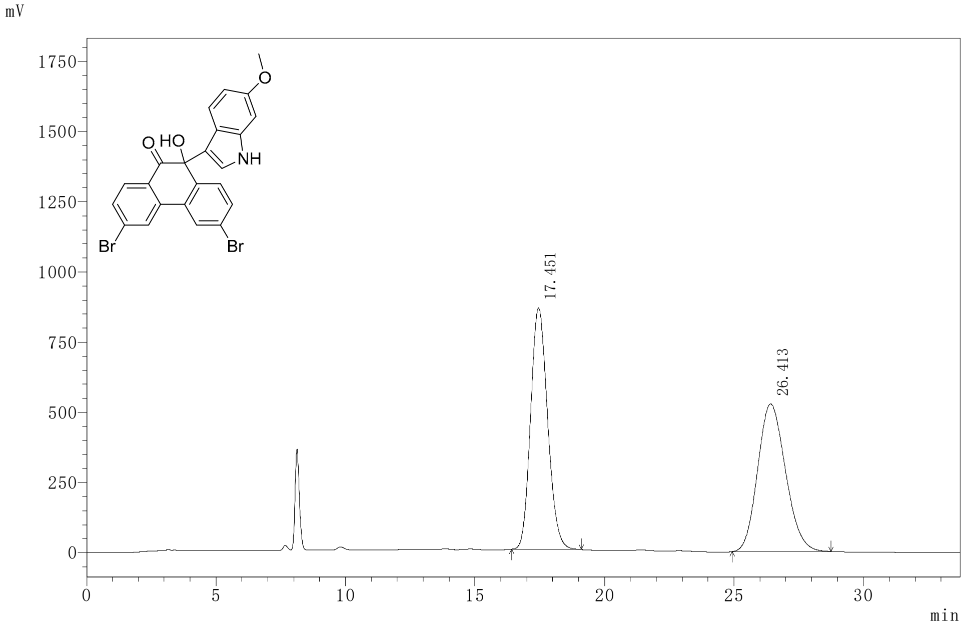

	Retention Time	Area	Height	Area%
1	17.451	39246697	859465	49.934
2	26.413	39350871	525625	50.066
Total		78597568	1385090	100.000

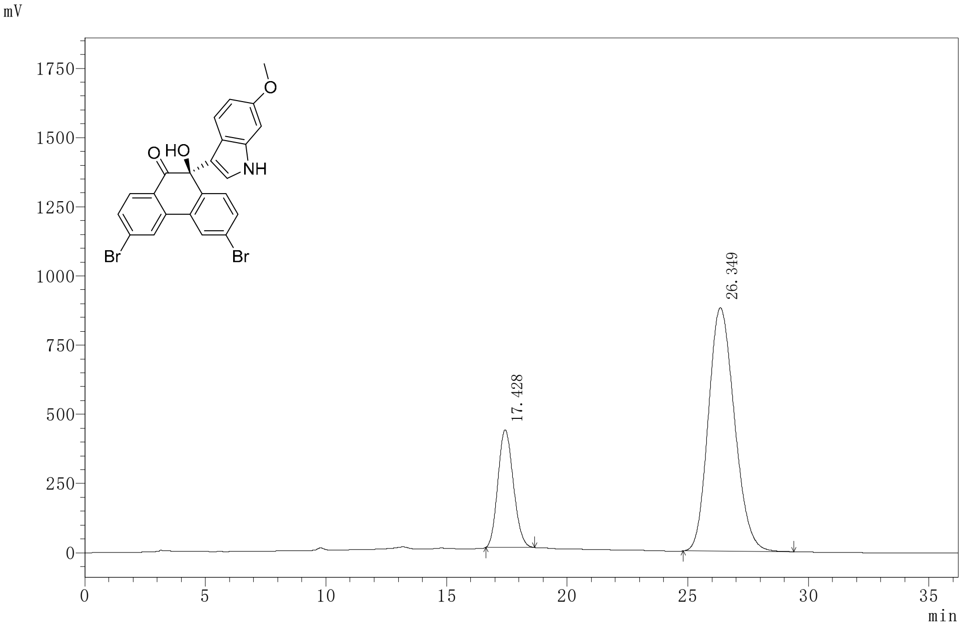

	Retention Time	Area	Height	Area%
1	17.428	18867031	424356	22.174
2	26.349	66219087	879422	77.826
Total		85086119	1303778	100.000

4g

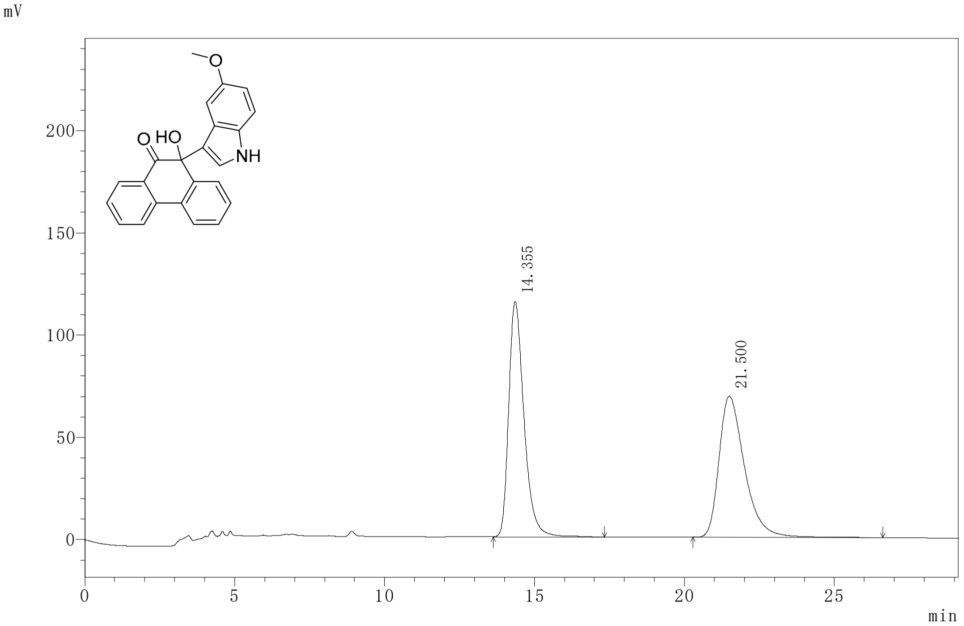

	Retention Time	Area	Height	Area%
1	14.355	4089445	115192	49.956
2	21.500	4096617	69051	50.044
Total		8186062	184243	100.000

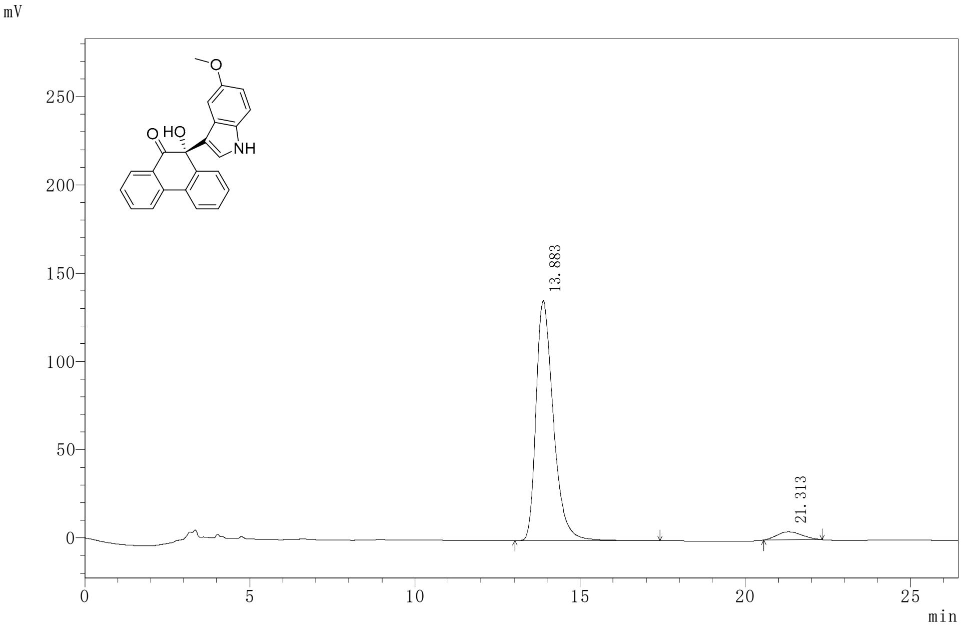

	Retention Time	Area	Height	Area%
1	13.883	4980727	136015	95.313
2	21.313	244931	4604	4.687
Total		5225658	140620	100.000

4w

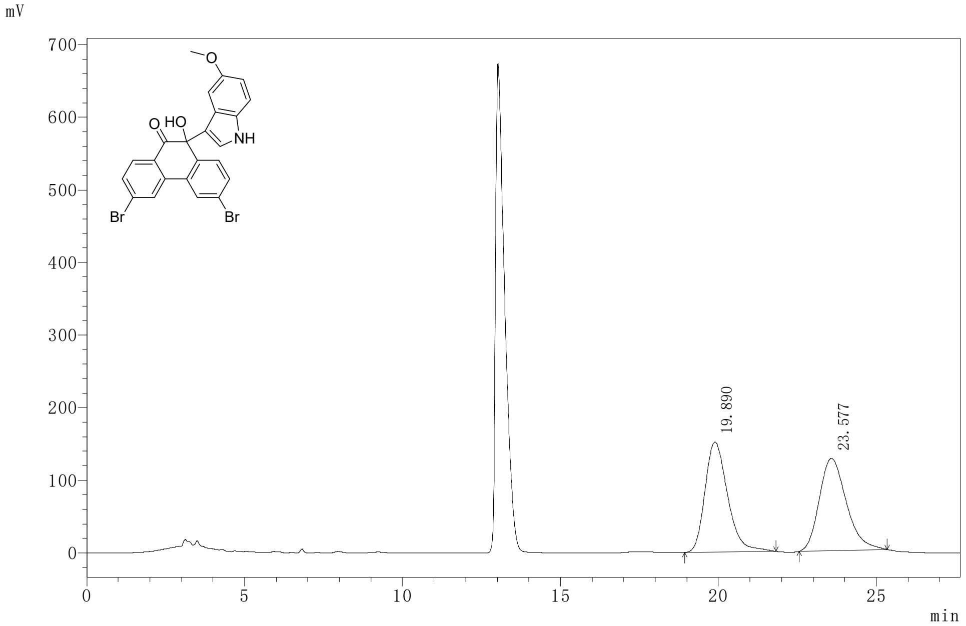

	Retention Time	Area	Height	Area%
1	19.890	7566017	151561	49.984
2	23.577	7570816	127078	50.016
Total		15136832	278640	100.000

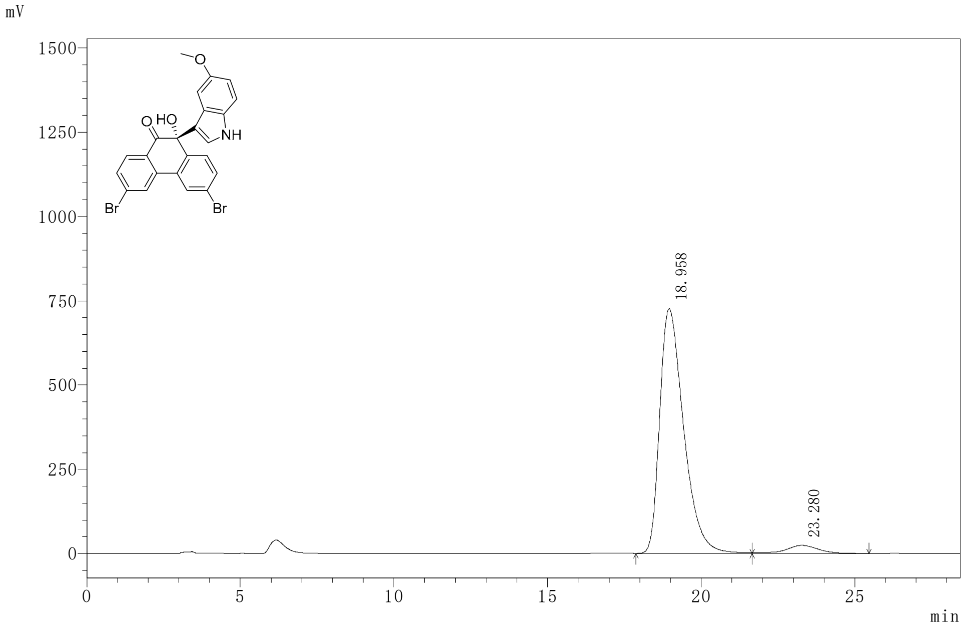

	Retention Time	Area	Height	Area%
1	18.958	39501711	726572	95.354
2	23.280	1924808	24568	4.646
Total		41426519	751140	100.000

4.X-Ray crystal data of comopound 4w

The single-crystal of **4w** were obtained from a solution of ethyl acetate and n-hexane.

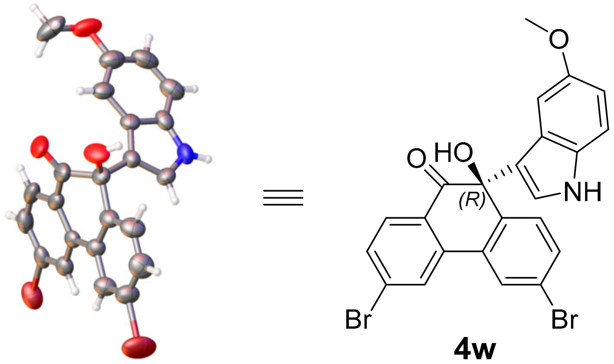

4w

CCDC 2375435

Table S2. Crystal data and structure refinement for 4w.

Identification code	**4w**
Empirical formula	$C_{23}H_{15}Br_2NO_3$
Formula weight	513.18
Temperature/K	223.00
Crystal system	monoclinic
Space group	$P2_1$
a/Å	7.5151(2)
b/Å	23.1572(6)
c/Å	11.2839(3)
α/°	90
β/°	101.525(2)
γ/°	90
Volume/Å^3	1924.13(9)
Z	4
ρ_{calc}g/cm^3	1.772
μ/mm^{-1}	5.564
F(000)	1016.0
Crystal size/mm^3	0.13 × 0.12 × 0.1
Radiation	CuKα (λ = 1.54178)
2Θ range for data collection/°	7.636 to 136.66
Index ranges	$-9 \leq h \leq 8$, $-27 \leq k \leq 27$, $-13 \leq l \leq 13$
Reflections collected	41359
Independent reflections	6990 [R_{int} = 0.0586, R_{sigma} = 0.0362]
Data/restraints/parameters	6990/2/525
Goodness-of-fit on F^2	1.065
Final R indexes [I>=2σ (I)]	R_1= 0.0496, wR_2= 0.1143

Final R indexes [all data]	R_1 = 0.0600, wR_2 = 0.1222
Largest diff. peak/hole / e Å^{-3}	1.34/-1.27
Flack parameter	0.004(9)

5. In vitro cytotoxicity assay

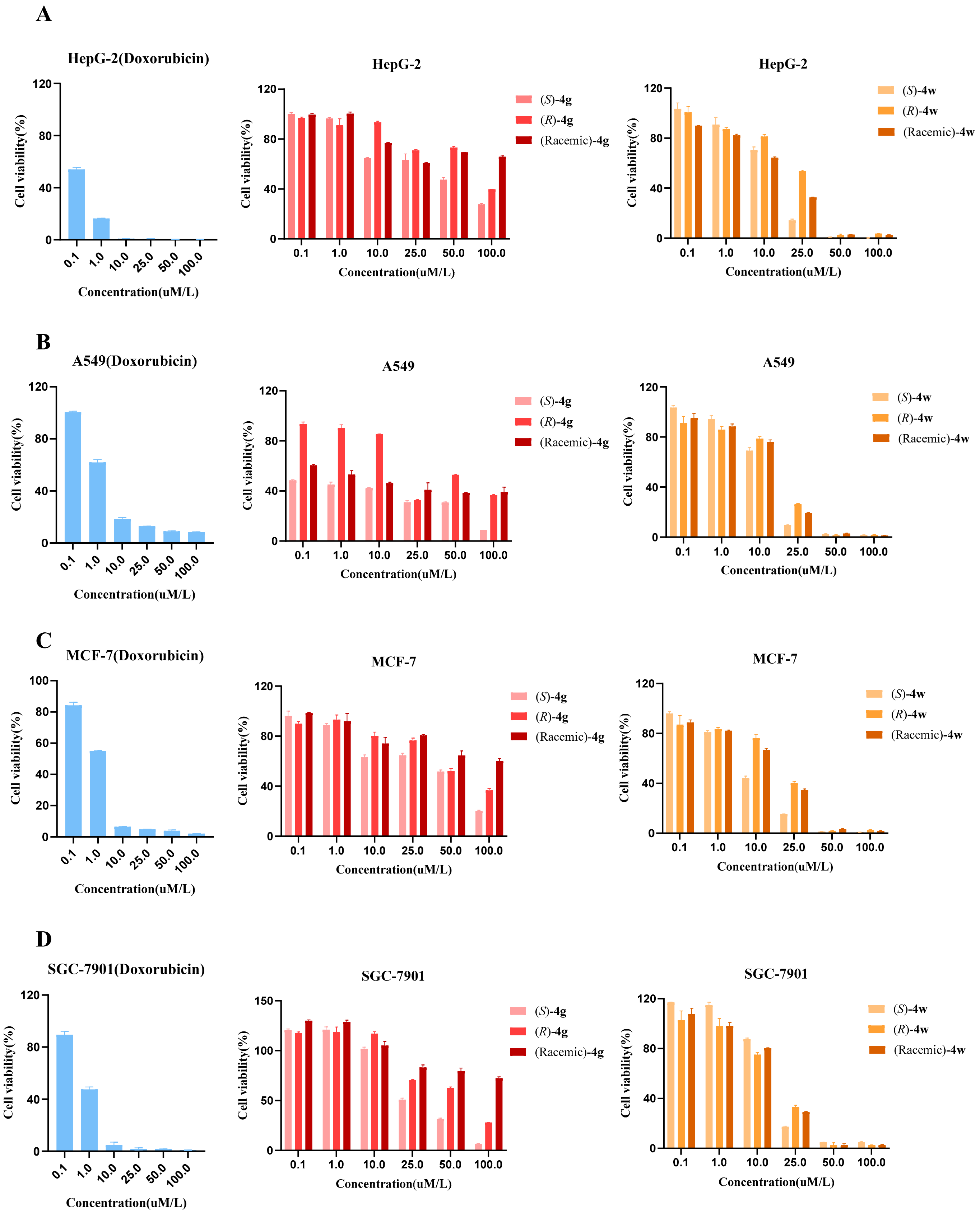

Figure 1. In vitro cytotoxicity assays (A) In vitro cytotoxicity of each group of compounds on

HepG-2 cells after 48 hours of incubation. (B) In vitro cytotoxicity of each group of compounds on A549 cells after 48 hours of incubation. (C) In vitro cytotoxicity of each group of compounds on MCF-7 cells after 48 hours of incubation. (D) In vitro cytotoxicity of each group of compounds on SGC-7901 cells after 48 hours of incubation.

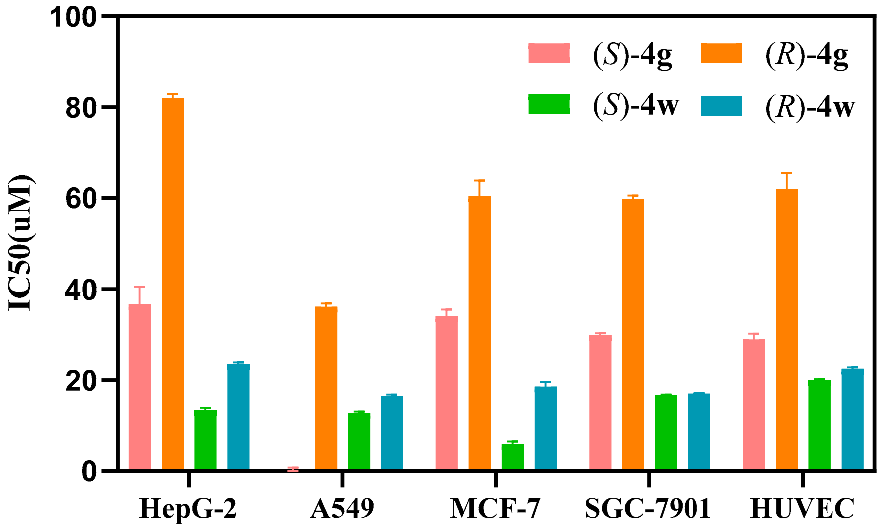

Figure 2. IC50 of **4g** and **4w** on normal cells compared with tumor cell.
